# Was Banfield right? New insights from a nationwide laboratory experiment

**DOI:** 10.1111/jors.12538

**Published:** 2021-06-15

**Authors:** Arnstein Aassve, Pierluigi Conzo, Francesco Mattioli

**Affiliations:** ^1^ Dondena Centre for Research on Social Dynamics and Public Policy Bocconi University Milano Italy; ^2^ Department of Social and Political Sciences Bocconi University Milano Italy; ^3^ University of Turin Torino Italy; ^4^ Collegio Carlo Alberto Torino Italy; ^5^ Department of Economics and Statistics ‘S. Cognetti de Martiis’ University of Turin, Campus Luigi Einaudi Lungodora Siena 100A Torino 10153 Italy

**Keywords:** cooperation, culture, north‐south divide, online experiments, reciprocity, social capital, trust

## Abstract

Since the pioneering study by Banfield, the North‐South gap in Italian social capital has been considered by international scholars as an example of how cultural diversity within a country can generate different developmental outcomes. Most studies, however, suffer from limited external validity and measurement error. This paper exploits a new and representative online lab‐experiment to assess social‐capital patterns in Italy. Unlike previous experiments, we do not inform participants about the geographic origins of their counterparts. This feature allows us to assess the North‐South gap in universal, as opposed to parochial, behavior. Results suggest that Southerners and Northerners do not systematically differ in generalized prosocial preferences. Only trustworthiness is higher among. Northerners, while they are statistically similar to Southerners in many other economic preferences such as cooperation, trust, expected trustworthiness, altruism, and risk tolerance. We also show that the gap in trustworthiness stems from the lower reciprocity of Southerners in response to large transfers, and it is characterized by the intergenerational transmission of norms. Possible policy implications are discussed.

## 1 INTRODUCTION

Trust is a key predictor of financial and economic success (e.g., Algan & Cahuc, 2010; Guiso et al., 2004; Knack & Keefer, 1997). Whereas trust is often discussed in terms of country differences, recent studies suggest that heterogeneity in economic preferences across countries is exceeded by within‐country heterogeneity (Falk et al., 2018). Indeed, Italy is an emblematic example where regional differences are stark. According to the latest European Values Study (EVS) data, in 2017, only 21.6% of Southern population declared to trust others as opposed to 27.5% in the North‐west and to 33.3% in the North‐east. Standard economic indicators follow a rather similar pattern. As a result, the North‐South divide in Italian social capital has attracted a great deal of attention. Over the last 60 years, a prominent literature has documented that social capital is scarce in the South (e.g., Banfield, 1958; Bigoni et al., 2019, 2016; Putnam et al., 1993), with trust, cooperation and civic engagement appearing lower compared to the North. These patterns are strongly correlated with the poor economic and institutional performance of the South (De Blasio & Nuzzo, 2010; Guiso et al., 2004; Helliwell & Putnam, 1995; Nannicini et al., 2013). The growth paths of the North and the South already diverged back in the 19th century (Daniele & Malanima, 2014; Federico et al., 2019; Felice, 2011), a divide that has persisted to date. Currently the unemployment rate in the South stands at 17.8% compared to 6.% in the North (source: Istat, 2018), *per capita* gross domestic product (GDP) differs from €19,347 to €35,811 (source: Eurostat, 2018) and added value *per* capita is 17,391 versus 32,110 (source: Istat, 2018).

Given shared religion, language and formal institutions, the Italian North‐South divide in social capital is indeed a conundrum. Influential studies suggest that the roots of the Italian dualism stem from cultural flaws of Southerners, who, for historical reasons, ended up with low levels of civicness. This literature, in particular, argues that institutional arrangements in the Middle Ages persistently affected the formation of self‐efficacy beliefs and entrepreneurial spirit, which are at the base of a culture of cooperation and, hence, economic growth (e.g., Banfield, 1958; De Blasio & Nuzzo, 2010; Guiso et al., 2008, 2016; Putnam et al., 1993). Other studies have shown evidence of the North‐South gap in social capital by means of money‐incentivized games conducted in the field (Bigoni et al., 2016) or in the lab (Bigoni et al., 2019), whereby—in both cases—participants received clues on their counterparts' origin. Experiments in the field carried out in four Italian cities, suggest lower *in‐group* trust and cooperation in the two cities located in the South; meanwhile, no differences in behavior, but only in beliefs about Southerners' cooperation emerged in lab experiments carried out with college students.

This paper revisits these issues by reporting results from a new nationwide representative laboratory experiment, from which both attitudinal and behavioral measures of social capital are elicited and compared across the five Italian macro‐areas. This Italian sample comes from the Trustlab project, which was started in 2016 by the OECD with the aim of acquiring internationally comparable and nationally representative data on social preferences through survey and experimental games (Murtin et al., 2018). Data collection occurred in October and November 2017 in Italy. The Italian sample of Trustlab also contains measures of the Big Five personality traits. This allows us to check whether personality differences (along with other socio‐demographic and economic characteristics) explain the geographical variation in social capital. Unlike previous experiments most similar to ours, Trustlab respondents do not receive any information on the geographic origins of the other participants. This feature allows us to focus on *universal*, as opposed to *parochial* social preferences as measured in the experimental games. A follow‐up survey administered in May and June 2018 provides additional information on residential history, the strength of family ties and betrayal aversion, which can potentially explain the results.

A representative survey of this kind has nonnegligible advantages over the previous studies. Banfield's ethnographic study is of little help for comparative purposes (Banfield, 1958). Others, though highly influential, were based on nonincentivized measures of social or civic capital (e.g., Guiso et al., 2004; Putnam et al., 1993). The few studies using incentivized designs emphasize in‐group preferences (Bigoni et al., 2016) or stereotyped group dimensions (Bigoni et al., 2019). With a fully representative sample where subjects provide answers both to the standard survey questions and participate in incentivized games, we take a step back and ask a set of questions of a more fundamental nature: Does a North‐South gap in social capital actually exist? Does it reflect a “national syndrome,” where Italians from the North and the South are characterized by a substantial diversity in terms of *generalized* prosocial preferences? And, finally, with different dimensions of social capital, which ones (if any) vary significantly across the country? With this survey, we are able to assess to what extent there is external validity to the North‐South social‐capital gap and the type of preferences that, keeping incentives constant, have been argued to differ through Italy.

Our study produces three novel results. First, a gap between the South and the rest of Italy emerges *only* in experimental trustworthiness, while no systematic differences are found in the vast majority of the other social‐capital dimensions that we test. These include engagement in voluntary work activities, unconditional and conditional cooperation in group‐interactions, expectations about others' trustworthiness, altruism, and risk aversion. Second, the North‐South gap in trustworthiness widens when the amount at stake is high, that is, when the opportunity cost associated to returning money (i.e., the trustor's transfer) increases Southerners reciprocate less than Northerners. While such inferior reciprocity is well anticipated by South‐Italian trustors, in the non‐Southern areas trust is, on average, below the optimal level as computed on the basis of the empirical distribution of trustworthiness. Third, the observed gap in trustworthiness is not accounted for by the endogenous migration from the South to the North, or by differences in betrayal aversion and in the strength of family ties. Our data show, instead, that it is inherited from parents: having a parent from the South is associated with lower trustworthiness, yet this effect is moderated by residing in the North.

Overall, this paper provides rather different results from those in previous studies, which have found a resilient North‐South divide in social capital. However, our findings based on generalized prosociality complement those of previous experiments that focus on local prosociality, that is, where the identity of narrowly defined groups (county or region) is made salient (Bigoni et al., 2019, 2016). In our experiment, Italians living in different macroareas do not seem to react in a systematically different way to the same incentives. If anything, they show a different behavior *only* in one specific dimension of social capital, namely reciprocity, and *only* under specific circumstances, that is when the uncooperative strategy becomes more tempting. Suggesting that North‐ and South‐Italians share a similar, universal, propensity to trust, donate and cooperate, these results may offer new insights into the historical debate about the existence and the economic effects of social‐capital differences across the Italian regions. They might also prove paradigmatic for other European countries facing economic disparities across their macroareas. Policies aimed at equalizing regional outcomes by increasing social capital should perhaps promote activities that build broader identities than one's own county or region. Moreover, they should target a specific component of social capital, namely reciprocity, while perhaps also addressing more compelling gaps elsewhere such as human‐capital differentials.

The remainder of the paper is organized as follows: In the next section we review relevant studies, and in Section 3 we discuss the Trustlab experiments. Then, descriptive results are presented. In Section 5 we report our econometric results, and in Section 6 we give in‐depth descriptions of the trustees' behavior. In Section 7 we assess additional explanations for the North‐South gap in trustworthiness. Then, in the final section we summarize our findings and offer conclusions.

## 2 BACKGROUND

The literature on the Italian North‐South gap in social capital dates back to *The Moral Basis of a Backward Society* by Banfield (1958), who made the first theoretical and empirical connection between culture and economic outcomes. On the basis of direct observations and interviews in a single Southern Italian town (which he gave the fictional name of “Montegrano”), Banfield concluded that a possible root of underdevelopment of the (entire) South could be explained by a cultural trait of Southerners, that is, the inability to cooperate with (and trust) nonfamily members. This inability would result from “amoral familism,” a social norm prescribing that societal welfare is subordinated to the interests of the individual and to those of the nuclear family. While certainly pioneering for that time, the research design obviously makes it difficult to generalize his findings.

Still, Banfield's study sparked considerable interest. Putnam et al. (1993) extended Banfield's analysis to the entire peninsula showing that regional differences in association density—a proxy for social capital—predict the North‐South gap in government functioning. Later, Guiso et al. (2004) show that self‐reported trust, political participation, and blood donation—which are typically higher in the Northern regions of Italy—can lead to larger investments in stocks, broader access to institutional credit, and less reliance on informal credit. Similarly, Nannicini et al. (2013) documented that the regions in Italy where social capital is scarce and cooperation is undervalued tend to be affected by poor institutional performance. In those regions, the authors argued, candidates are elected on the basis of citizens' personal interest rather than social welfare.

Most of these studies measure social capital directly, through nonincentivized trust questions, or indirectly, through data on socio‐political participation (e.g., blood donation and voting turnout). However, the question of what type of preferences and beliefs underlie the chosen measures of social capital, and how they are distributed geographically, remain open issues (Bowles & Gintis, 2002; Delhey et al., 2011; Glaeser et al., 2000).

A noteworthy contribution to the debate on this Italian dualism was recently made by Bigoni et al. (2016), who examine, through a lab‐in‐the field experiment, the North‐South gap in social preferences. Their results document that, when given the same incentives, Italians display different in‐group preferences: respondents from the North are more trusting and willing to cooperate with participants from their own province than respondents from the South. In a later experiment, they show that this gap in cooperation is not due to underlying differences in prosocial preferences. Rather, it originates from Southerners' higher levels of aversion to social risk, and from their pessimistic expectations about others' cooperativeness (Bigoni et al., 2019). These two studies rely on money‐incentivized measures, yet their samples are not representative at the national level: again, the study's generalizability is problematic.

The first study carried out in‐field experimental games on a representative sample, though only in four Italian cities (two in the North and two in the South), where subjects are primed about the geographical origin of their counterparts.^1^ Unless one assumes that social preferences in these cities represent those of *all* the other residents in Southern or Northern cities, the authors' inferences about the nonsampled areas remain questionable. External validity represents a more serious concern in the second study, where the experiments are conducted with university students in Bologna. As the authors acknowledge, this sample is not representative, and results might be affected by self‐selection due to the South‐North migration of students.

Moreover, even when lab‐in‐the‐field experiments were performed on a representative population at the county level as in Bigoni et al. (2016), anonymity was not fully satisfied. Indeed, participants could infer their counterpart's characteristics by chatting in the waiting room before the experiment; and they were, in any case, told about the geographical origin of the counterpart in the instructions (Bigoni et al., 2019, 2016). In these cases, rather than preferences for trust in and cooperation with an unknown, generalized other, the observed behavior can be interpreted as *in‐group* trust and cooperation. Hence, the documented North‐South difference in such *parochial* preferences may not mirror the underlying distribution of *generalized* social preferences and individual intrinsic propensities to behave prosocially across Italian macroareas.

## 3 TRUSTLAB: CONCEPTS AND MEASUREMENT

In contrast to the studies reviewed above, we use a new representative sample of the general population in terms of age, gender, and income. The sample counts over 1000 participants distributed across the Italian macroareas in proportion to the actual distribution of population. Participants take part in the study on an online platform articulated in two main sections, namely an experimental part and a survey part.^2^ The Italian sample is part of a collaborative effort with the OECD and other research institutions and governments under what is known as the Trustlab project (Murtin et al., 2018).^3^ The main aim of Trustlab is to analyze social and institutional trust through cutting‐edge methodological approaches.

### 3.1 The trust game (*TG*)

In the first section, people take part in a series of experimental games. In the *TG* (Berg et al., 1995) each respondent is given €10. They, then, play both the role of “sender” and “receiver” (Figure A1 in Appendix A). First, the sender decides whether to transfer money from his or her endowment to another participant s/he is randomly coupled with. Transferred money is, next, tripled and added to the receiver's endowment, who will finally decide whether to transfer money back to the sender. Hence, in the role of sender each respondent chooses whether and to what extent to trust an unknown person, whereas as receiver each respondent reveals his or her degree of trustworthiness and reciprocity by transferring back money for each hypothetical transfer of the trustor (strategy method). In other words, when playing as receivers, respondents report how much they would transfer back for each possible amount the trustor could send (from €0 to €10).^4^

Moreover, in a hypothetical scenario participants state how much they expect a trustor sending €5 will be reciprocated by a random partner who receives €15 (first order beliefs).

As in Bigoni et al. (2018), the TG is characterized by the reversal of roles in a sequential order. Each participant acts first as trustor and then as trustee.^5^ However, we can confidently exclude carry‐over effects. Feedback is provided to Trustlab participants only at the end of the survey. Respondents are informed that the experimental task to compute their final payment and the partner(s) they are matched with are determined randomly within 48 h of the completion of the survey.

### 3.2 The public goods game (*PGG*)

The *PGG* (Fehr & Gächter, 2000) provides a framework for assessing people's level of unconditional and conditional cooperation in group‐interactions (Figure A2 in Appendix A). Participants are randomly sorted into groups of four and decide whether to devote any part of their own endowment (€10) to a common project. Contributions by all group members go into a common pool of resources and get multiplied by a factor of 1.6. The resources “generated” through the common project are split equally among the four group members, irrespective of their contributions. A participant's payoff is equal to the part of her endowment not offered to the project, supplemented by a quarter of total contributions collected within his or her group.

In the first version of the game, Trustlab participants decide first how much to contribute to the common project at the same time as other group members, thus revealing “unconditional cooperation.” In a second version of this game, they are also told what the average contribution of members is; this allows for an understanding of whether (and in what direction) they condition their own contribution to that of the others (“conditional cooperation”).

### 3.3 The dictator game (DG) and the risky decision game

Participants are also paired in the DG (Kahneman et al., 1986). Each respondent (sender) decides whether to transfer any part of his/her endowment of €10 to his/her partner (receiver), knowing that there is not going to be a second step (i.e., the receivers do nothing). The money transfer is intended to measure participants' unconditional altruism.

The behavioral section in Trustlab ends with a lottery choice to assess attitudes towards risk (Eckel & Grossman, 2002). Participants choose one out of six possible lotteries, distinguished by an increasing differential in payoffs in the case of success and failure (occurring with equal odds). The higher the payoffs differential, the more pronounced the participants' risk‐taking (see experimental instructions in the Supporting Information Materials in Appendix C).

Notice that, unlike previous experiments (e.g., Bigoni et al., 2019, 2016), in our study a random sample of Italians were playing money‐incentivized games in front of a computer. Thus, they could not physically see their counterparts. Moreover, no information about their counterparts' geographical origin was provided. This approach allows us to capture Italians' *generalized* as opposed to *parochial* preference for trust and cooperation. Generalized trust and cooperation, that is, the tendency to trust and cooperate with a generalized, nonidentifiable other, pertain more to the “bridging” social capital, which is based on bonds formed across diverse social groups, than to the “bonding” social capital, which cements only homogenous groups (e.g., Putnam, 2000). Since the former type of social capital has been associated with a well‐functioning society (Putnam, 2000),^6^ our experimental results may well fit into the debate about the cultural roots of underdevelopment.

### 3.4 The survey

**Figure 1:**
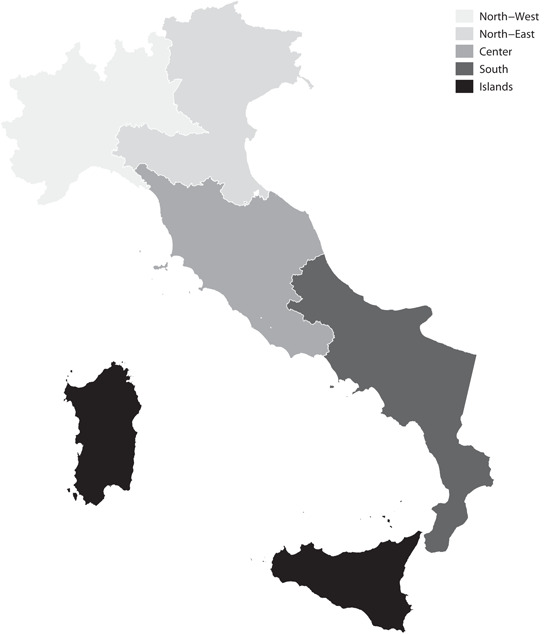
The Italian macroareas. Nomenclature of Territorial Units for Statistics 1 classification of Italian territorial units according to EUROSTAT

The second section of Trustlab is a standard survey with numerous modules (see Appendix C to explore all the questions included in the survey). Respondents self‐report their level of trust in other people (Generalized Trust Question [*GTQ*]^7^) and institutions as well as other attitudes, such as the frequency of voluntary work and of encounters with friends,^8^ intrinsically linked to social capital (Sampson, 1988; Welzel et al., 2005). They also provide information on their own socio‐demographic and economic characteristics. The survey records geographical information down to the municipality level. But the geographical level of interest for assessing the existence of a North‐South divide is that of macroareas: Nomenclature of Territorial Units for Statistics (NUTS)‐1 areas in the Eurostat nomenclature. Italy has, as Figure 1 shows, five different macroareas.

A distinctive feature of the Italian Trustlab sample is a battery of questions investigating respondents' personality traits. The survey includes a reduced 15‐item version of the original Big Five Inventory (BFI) by John et al. (1991), already used in well‐known surveys such as the GSOEP, the BHPS, the UKHLS and the HILDA. Trustlab is, to the best of our knowledge, the first survey where this short BFI is administered on a representative Italian sample: questions have been translated from English, with adjustments to an Italian translation provided in Ubbiali et al. (2013). Factorial analyses have been conducted on the personality data to check the internal consistency of the short Italian BFI, the validity of which is also assessed through convergent and discriminant analyses with comparable BFIs from other surveys (Aassve et al., 2018). To be included in the empirical analysis, each personality trait (agreeableness, conscientiousness, neuroticism, extraversion, and openness) is determined by averaging the answers to the three items capturing the respective personality dimension. These are appropriately recoded whenever the questions were negatively‐worded to reduce acquiescence bias.

## 4 DESCRIPTIVE STATISTICS

The Trustlab sample is representative of the Italian population in terms of gender, age, income and geographical distribution. Table 1 summarizes the composition of the sample by main socio‐demographic and economic characteristics. A third of the sample completed tertiary education, while slightly more than half the sample holds a high school or a lower level diploma as their highest educational attainment. The remaining 17% of the sample is composed of university dropouts or people with a nontertiary diploma. Concerning the education of respondents' parents, fathers are moderately more educated than mothers. One out of five respondents is out of the labor force; a little less than a fifth of those who are in the labor force are unemployed, while working people are divided between employed and self‐employed with a ratio of 5:1. Looking at marital status, the majority of the sample is married, while 36.5% is not and the residual 10th is widowed, divorced or separated. Fourteen percent of respondents live in a rural area, three percentage points more than those who live in large metropolitan areas; almost two thirds of the sample live in towns or villages, whereas the remaining 14.5% lives in small to medium densely populated residential areas. The mean yearly income in the sample is as high as €16,000 but a standard deviation of more than €20,000 suggests the existence of a great deal of heterogeneity. In particular, there are a number of outliers in the upper part of the income distribution, as the density is heavily skewed to the right. On average, the households of respondents in Trustlab include two other people.

**Table 1:** Socio‐demographic composition of the sample

Variable	Observations	Share (%)
Place of residence		
North‐West	299	29.5
North‐East	176	17.3
Center	211	20.8
South	223	22.0
Islands	106	10.4
Gender		
Male	500	49.3
Female	515	50.7
Age		
18–24	119	11.7
25–34	199	19.6
35–44	236	23.3
45–54	264	26.0
55–64	197	19.4
Education		
High school or less	516	50.8
Some college or other nontertiary	173	17.0
Tertiary diploma	326	32.1
People in household		
One	115	11.3
Two	223	22.0
Three	282	27.8
Four	309	30.4
Five or more	86	8.5
Income		
0–350	248	24.4
400–7000	158	15.6
7600–20,000	241	23.7
20,500–28,000	172	16.9
29,000–350,000,000	196	19.3
Employment status		
Employed	565	55.7
Self‐employed	112	11.0
Unemployed	134	13.2
Inactive	204	20.1
Marital status		
Single	370	36.5
Married	551	54.3
Other	94	9.3
Urbanization of residence area		
Rural area	146	14.4
Village	375	36.9
Town	229	22.6
Small/medium metropolitan area	147	14.5
Large metropolitan area	118	11.6
Father's education		
Less than secondary	545	53.7
Secondary or tertiary	470	46.3
Mother's education		
Less than secondary	592	58.3
Secondary or tertiary	423	41.7

Comparison of the distribution of respondents by socio‐demographic and economic characteristics and by NUTS level between the Trustlab sample and the actual Italian population as of 2017 suggests that the sample is close to representative. Table 2 shows the share of Italian population by macroareas and by the main socio‐demographic characteristics retrieved from official national statistics as of 2017 or the closest period (Istat, Bank of Italy), and the same shares occurring in the Trustlab sample employed for estimations. While the Trustlab sample reflects most characteristics of the Italian population including income, it over‐represents highly educated citizens from all macroareas and employed citizens from the South. This could be due to the nature of the on‐line experiment, which requires a computer/tablet with internet connection for participation. Hence a certain degree of self‐selection into the experiment is expected, an issue that we deal with in Section 5 and Appendix B.

**Table 2:** Sample representativeness

	Official statistics						Trustlab sample					
		North			South and Islands			North			South and Islands	
Variables	Italy	North‐West	North‐East	Center	South	Islands	Italy	North‐West	North‐East	Center	South	Islands
Gender												
Male	48.7	48.7	48.7	48.3	48.8	48.8	49.3	47.2	47.7	49.8	53.8	47.2
Female	51.3	51.3	51.3	51.7	51.2	51.2	50.7	52.8	52.3	50.2	46.2	52.8
Age												
18–24	11.2	10.6	10.7	10.5	12.6	12.1	11.7	10.7	13.6	9	15.3	9.4
25–34	17.9	17.1	16.9	17.4	19.5	19.2	19.6	19.4	22.2	18	19.3	19.8
35–44	22.6	22.8	22.8	23.2	22	22.1	23.3	23.1	20.5	24.6	23.8	24.5
45–54	26.4	27.5	27.5	27	24.7	24.8	26	27.1	26.1	27.5	20.6	31.1
55–64	21.8	22.1	22.1	22	21.2	21.8	19.4	19.7	17.6	20.9	21.1	15.1
Education												
Less than high school	41.2	39	36.7	35.3	48.7		9.4	8.7	15.3	5.7	9.1	
High school diploma	42.3	43.3	46	44.5	38.2		58.5	60.5	55.1	56.4	59.9	
Tertiary diploma	16.5	17.7	17.3	20.3	13.1		32.1	30.8	29.6	37.9	31	
Employment status												
Employed and Self‐emp.	58	66.2	67.4	62.8	44		66.7	67.9	72.2	69.2	61.1	
Unemployed	7.5	5.4	4.6	7.1	10.8		13.2	11.4	12.5	10.9	16.7	
Inactive	34.6	28.3	28	30.1	45.2		20.1	20.7	15.3	19.9	22.2	
Household Income												
First quintile	20	20.4		15	22.6		20.3	13.9		20.9	29.2	
Second quintile	20	17.2		17	26		20	17.1		18	25.5	
Third quintile	20	17.5		20.6	23.4		21.2	22.7		23.2	17.6	
Fourth quintile	20	20.6		22.9	17.3		18.7	21.7		19	14.3	
Fifth quintile	20	24.3		24.4	10.6		19.8	24.6		19	13.4	

*Note*: Official statistics on the Italian population's gender, age, education and employment status retrieved from Istat data warehouse (as of 2017 or closest date available); on household income from Bank of Italy's *Survey on Household Income and Wealth* (2016).

Trustlab collects several experimental measures of trust‐related concepts and other social preferences. To operationalize such concepts we consider in this study: (i) the amount sent by the sender in the first step of the TG as a measure of trust (*trust*); (ii) the amount sent back by the receiver in the TG—averaged over the eleven hypothetical transfers of the sender—as a measure of trustworthiness (*trustworthiness*); (iii) the amount expected back from the receiver in the case of a €5 transfer as a measure of expected trustworthiness (*expected trustworthiness*); (iv) the amount contributed to the common project in the PGG as a measure of cooperation (*cooperation*); (v) an index of conditional contributions as a measure of reciprocity in the PGG (*conditional cooperation*)^9^; (vi) the amount sent in the DG as a measure of altruism (*altruism*); and (vii) the lottery chosen as a measure of experimental risk attitudes^10^ (*risk propensity*), with later lotteries implying high‐risk tolerance.^11^

Table 3 reports the descriptive statistics of the aforementioned experimental outcomes and of the main survey measures used in this paper. Experimental and self‐reported trust have approximately the same mean, though experimental trust appears to be more dispersed around the mean. On average, trustworthiness is slightly below expected trustworthiness, with similar distributions in terms of variability, too. Whereas respondents expect on average that the amount returned by trustees is 39.6% of the endowment, the actual trustworthiness they show as trustees amounts to some 35.5%. The average respondent is highly altruistic since s/he tends to split his/her endowment equally with an unknown Italian. Also, Italian respondents show a preference for cooperation since they contribute an average of three fifths of their endowments to public goods; they also appear to be conditional reciprocators, meaning that they are willing to contribute more if people around them contribute at the same levels. There is a general prevalence of risk aversion, as showed by preference for safe rather than risky lotteries, although the dispersion around the mean suggests a great deal of heterogeneity. Self‐reported measures of social capital suggest that Italian respondents are only rarely involved in voluntary activities, while they get together with friends quite often during the week. Table 4 reports pairwise correlations between main experimental and survey outcomes.

**Table 3:** Descriptive statistics of main dependent variables

Variables	Obs	Mean	*SD*	Min	Max
Trust	1015	6.00	2.93	0	10
Trustworthiness	1015	9.44	5.18	0	25
Expected trustworthiness	1015	9.89	5.51	0	25
Altruism	1015	4.32	2.29	0	10
Cooperation	1015	6.00	2.87	0	10
Conditional cooperation	1015	0.63	0.42	−1	1.18
Risk propensity	1015	2.84	1.59	1	6
Generalized trust question	1010	6.08	2.11	0	10
Frequency of voluntary works	1015	0.75	0.90	0	4
Frequency of encounters with friends	1015	2.14	0.93	0	4

**Table 4:** Correlation matrix of main outcomes

	Trust	Trustw.	Expec. trustw.	Altr.	Coop.	Cond. coop.	Risk prop.	GTQ	Volun. works
Trustworthiness	0.367								
Expected trustw.	0.265	0.469							
Altruism	0.369	0.334	0.324						
Cooperation	0.444	0.284	0.213	0.362					
Cond. coop.	0.002	0.034	−0.061	−0.084	0.048				
Risk propensity	0.067	0.014	0.066	0.087	0.092	−0.094			
GTQ	0.09	0.091	0.147	0.142	0.096	−0.015	0.003		
Voluntary works	0.019	0.055	0.091	0.082	0.032	−0.05	0.027	0.192	
Enc. with friends	0.015	0.038	0.024	0.052	0.06	−0.005	0.082	0.138	0.214

Regarding personality, the average Italian respondent in Trustlab shows higher degrees of agreeableness and conscientiousness than the other traits, as shown in Table 5. Medium to high openness also characterizes most respondents, while they appear to be extraverted and neurotic to a lower extent, although the distributions of the latter personality traits are more dispersed.

**Table 5:** Descriptive statistics of personality traits

Personality trait	Obs	Mean	*SD*	Min	Max
Openness	1001	3.62	0.76	1	5
Conscientiousness	1001	3.81	0.76	1	5
Extraversion	1011	2.94	0.80	1	5
Agreeableness	1003	3.90	0.68	1.33	5
Neuroticism	1012	3.08	0.84	1	5

**Figure 2:**
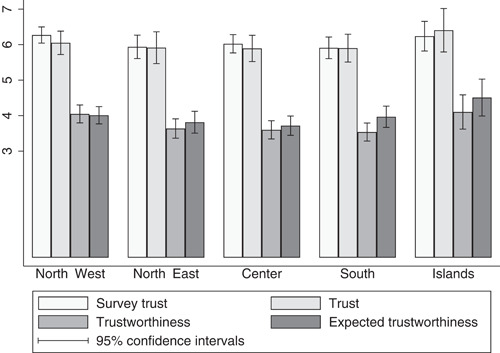
Self‐reported trust and trust games, outcomes across the Italian macroareas. Variables' means and 95% confidence intervals by Italian macroareas. Variation range of all variables rescaled to vary between 0 and 10. The *generalized trust question* asks respondents “In general, how much do you trust most people?”. *Trust* is the amount of money sent by trustors to trustees in the trust game. *Trustworthiness* is the amount of money returned by trustees to trustors in the trust game. *Expected trustworthiness* is the amount of money trustors expect to be returned by trustees in the trust game

Figure 2 shows the average levels of self‐reported and experimental trust, expected trustworthiness and trustworthiness across the five Italian macroareas. While there are nondramatic differences in self‐reported and experimental trust, the South ranks the lowest in trustworthiness, while insular Italy (Sardinia and Sicily) scores remarkably high in expected trustworthiness.^12^

**Figure 3:**
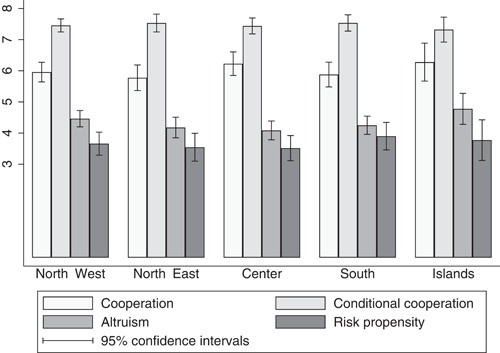
Cooperation, conditional cooperation, altruism and risk propensity across the Italian macroareas. Variables' means and 95% confidence intervals by Italian macroareas. Variation range of all variables rescaled to vary between 0 and 10. *Cooperation* is the amount of money contributed by participants to the common project in the public goods game, unconditional on other participants' contributions. *Conditional cooperation* is the amount of money contributed by participants to the common project in the public goods game, conditional on other participants' contributions. *Altruism* is the amount of money sent by dictators to receivers in the dictator game. *Risk propensity* is the lottery chosen by participants in the risk game (the stronger risk aversion, the lower the measure)

**Figure 4:**
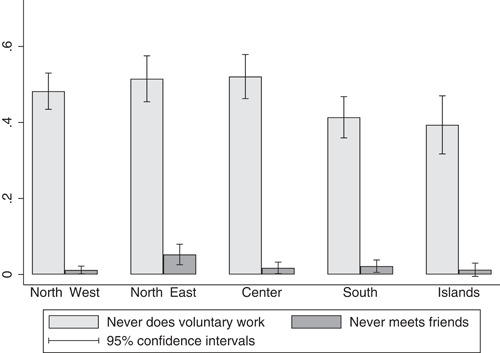
Social engagement and voluntary work across the Italian macroareas

When looking at the within‐country distribution for other preferences, we find no evidence of an inferior level of cooperation (conditional or unconditional) and altruism in the South, nor, indeed, significant differences in risk propensity (Figure 3).^13^ In addition, the South does not rank lower than the North in terms of voluntary work and social interactions, which can be considered as other proxies for social capital. Interestingly, the share of respondents who are not involved in social interactions and voluntary work is lower in the South than in the North (Figure 4).^14^

Other significant differences are found in terms of personality. Each personality trait is computed as a simple mean of the three respective items asked in the survey, with harmonized answer ranges. We exclude from estimation respondents with missing information on either of the three personality items measuring each trait.^15^ Figure A3 in Appendix A plots the coefficients of the macroarea dummies (the North‐west being the reference category) from a regression of personality traits on socio‐demographic characteristics. Interestingly, the South scores higher in agreeableness, openness, and conscientiousness. While the first two are shown to be positively correlated with trust (Dohmen et al., 2008; Freitag & Bauer, 2016; McCarthy et al., 2017), there is less consensus about whether conscientiousness spurs (Freitag & Bauer, 2016; McCarthy et al., 2017) or hampers (Dohmen et al., 2008) trust.

Summarizing this descriptive analysis, we do not observe a significant gap in social capital between the South of Italy and the other Italian macroareas. Apart from lower levels of trustworthiness, Southerners display on average similar (or in some cases higher) generalized prosocial preferences than Northerners.

## 5 ECONOMETRIC FINDINGS

To control for potential confounders, as well as to check for the mediating role of the Big Five personality traits and other preferences, we run ordinary least square (OLS) regressions.^16^ Our main model is expressed as in Equation (1):

(1) $$Y_{ij}=\alpha_{0}+\underset{j}{\sum }\alpha_{j}M_{j}+\underset{k}{\sum }\beta_{k}X_{ik}+\epsilon_{ij}$$

where $Y_{ij}$ is the experimental or survey measure of the social capital of individual i living in the macroarea j, and $M_{j}$ are four dummies taking value one if respondent i belongs to macroarea j (i.e., South, Insular, Central, and North‐east—the reference category is the North‐west), and zero otherwise. In an alternative specification we replace macroarea dummies with a single dummy variable (*South*) equal to one for respondents living in the South (the omitted category being the rest of Italy). We control for a set of k socio‐demographic variables ($X_{ik}$) including gender, age, household size, education, income, job status, marital status, and the size of residential area, which would capture differences in the size (and the type of) social networks. Since parents' level of education has been shown to predict children's prosociality (e.g., Dohmen et al., 2011; Lundborg et al., 2014; Pishghadam & Zabihi, 2011), we control as well for the level of education of the respondent's mother and father. $\alpha_{0}$ is a constant term, while $\epsilon_{ij}$ is an error term. In alternative specifications we also include the Big Five personality traits and other experimental measures of social capital different from the outcome at stake.^17^

In Table 6 we report estimation results with survey trust, trust, trustworthiness, and expected trustworthiness as dependent variables. Results show that Southerners display lower levels of self‐reported trust (Columns 1–2) and trustworthiness (Columns 5–6), while no significant differences are found in the average amount sent (Columns 3–4) or expected in the TG (Columns 7–8). The inclusion of personality traits does not change the main findings, thereby suggesting that the differences in personality shown in Figure A3 do not explain the North‐South gap in terms of the generalized trust and trustworthiness we observe in the data.^18^ These results are confirmed when moving to a more parsimonious model in which the four macroareas dummies are replaced by the South indicator (Table 7), though the results remain statistically significant only for trustworthiness.

**Table 6:** Macroareas differences in generalized trust, experimental trust, trustworthiness and expected trustworthiness

	GTQ		Trust		Trustworthiness		Expected trustworthiness	
	(1)	(2)	(3)	(4)	(5)	(6)	(7)	(8)
North‐east	−0.330	0.276	−0.190	−0.211	−1.058**	−1.135**	−0.522	−0.483
	(0.205)	(0.194)	(0.284)	(0.288)	(0.484)	(0.489)	(0.514)	(0.520)
Center	−0.296	−0.264	−0.135	−0.192	−1.154**	−1.133**	−0.591	−0.596
	(0.181)	(0.175)	(0.267)	(0.273)	(0.484)	(0.492)	(0.487)	(0.499)
South	−0.375*	−0.394*	−0.124	−0.0706	−1.276***	−1.407***	0.0577	−0.123
	(0.202)	(0.199)	(0.266)	(0.271)	(0.468)	(0.469)	(0.506)	(0.509)
Islands	0.0433	−0.0268	0.404	0.510	0.234	0.418	1.349*	1.303*
	(0.242)	(0.236)	(0.353)	(0.351)	(0.696)	(0.713)	(0.733)	(0.732)
Openness		−0.0823		−0.212		−0.389		−0.437*
		(0.0950)		(0.133)		(0.239)		(0.250)
Conscientiousness		−0.211*		−0.0661		−0.174		−0.0581
		(0.114)		(0.156)		(0.286)		(0.301)
Extraversion		0.480***		−0.159		−0.0281		0.615***
		(0.0891)		(0.127)		(0.205)		(0.213)
Agreeableness		0.241**		0.123		0.330		0.332
		(0.117)		(0.151)		(0.282)		(0.297)
Neuroticism		−0.447***		−0.243**		−0.216		−0.0860
		(0.0866)		(0.115)		(0.198)		(0.212)
Female	−0.0551	−0.0160	−0.611***	−0.455**	−0.416	−0.303	0.0386	−0.0877
	(0.146)	(0.146)	(0.196)	(0.205)	(0.346)	(0.365)	(0.361)	(0.377)
Age								
25–34	0.0130	−0.0884	0.361	0.396	0.691	0.474	1.146	1.147
	(0.271)	(0.277)	(0.381)	(0.396)	(0.694)	(0.717)	(0.698)	(0.717)
35–44	0.266	0.192	−0.0722	−0.110	−0.160	−0.447	0.915	0.817
	(0.289)	(0.296)	(0.411)	(0.425)	(0.695)	(0.721)	(0.743)	(0.765)
45–54	0.0852	0.0521	−0.206	−0.264	−0.0212	−0.150	0.969	1.034
	(0.289)	(0.298)	(0.411)	(0.423)	(0.719)	(0.753)	(0.762)	(0.780)
55–64	0.244	0.267	−0.344	−0.376	−0.0192	−0.130	1.736**	1.805**
	(0.305)	(0.314)	(0.427)	(0.437)	(0.736)	(0.770)	(0.781)	(0.799)
People in household	0.0688	0.0713	−0.0785	−0.0643	0.0475	0.0485	0.0224	0.0520
	(0.0614)	(0.0599)	(0.0858)	(0.0866)	(0.151)	(0.155)	(0.168)	(0.174)
Some college/nontertiary	−0.0815	−0.00720	0.370	0.457*	0.0714	0.0500	−0.671	−0.650
	(0.203)	(0.195)	(0.265)	(0.268)	(0.441)	(0.433)	(0.490)	(0.478)
Tertiary education	0.0622	0.120	0.00929	0.0278	0.158	0.261	−0.633	−0.516
	(0.164)	(0.161)	(0.218)	(0.223)	(0.400)	(0.411)	(0.431)	(0.445)
Personal income (log)	0.0456	0.0236	0.0792**	0.0608	0.0649	0.0144	0.0387	0.0232
	(0.0303)	(0.0289)	(0.0380)	(0.0394)	(0.0686)	(0.0690)	(0.0749)	(0.0770)
Self‐employed	−0.472**	−0.455**	0.412	0.390	−0.544	−0.409	−0.889	−0.772
	(0.226)	(0.213)	(0.319)	(0.319)	(0.468)	(0.476)	(0.558)	(0.556)
Unemployed	−0.107	−0.165	0.216	0.127	−0.134	−0.463	−0.885	−0.962
	(0.223)	(0.221)	(0.344)	(0.356)	(0.617)	(0.627)	(0.608)	(0.628)
Inactive	−0.231	−0.328	0.145	−0.0422	−0.207	−0.657	−0.806	−0.921
	(0.239)	(0.235)	(0.326)	(0.338)	(0.591)	(0.599)	(0.617)	(0.637)
Married	0.433**	0.300*	0.0730	−0.0379	−0.567	−0.632	−0.471	−0.701
	(0.178)	(0.175)	(0.256)	(0.261)	(0.452)	(0.470)	(0.506)	(0.519)
Other	0.0150	−0.318	−0.128	−0.392	−0.353	−0.581	−1.080	−1.285*
	(0.284)	(0.287)	(0.372)	(0.379)	(0.656)	(0.679)	(0.702)	(0.738)
Rural area	0.442**	0.336*	0.316	0.293	−0.213	−0.182	0.474	0.444
	(0.200)	(0.196)	(0.293)	(0.300)	(0.496)	(0.506)	(0.540)	(0.543)
Town	0.292	0.263	0.310	0.345	−0.0413	0.0503	0.0421	0.142
	(0.178)	(0.178)	(0.248)	(0.251)	(0.433)	(0.441)	(0.477)	(0.484)
Small/medium metro. area	−0.0923	−0.0427	0.250	0.0968	−0.0244	−0.0368	0.466	0.157
	(0.219)	(0.205)	(0.292)	(0.296)	(0.525)	(0.527)	(0.568)	(0.568)
Large metropolitan area	0.311	0.221	0.220	0.253	0.0855	0.210	0.0361	0.00883
	(0.232)	(0.221)	(0.327)	(0.333)	(0.654)	(0.670)	(0.621)	(0.639)
Educated father	−0.0409	−0.0578	−0.214	−0.211	−0.201	−0.119	−0.186	−0.236
	(0.162)	(0.156)	(0.226)	(0.229)	(0.384)	(0.389)	(0.407)	(0.406)
Educated mother	−0.0383	−0.0121	0.00467	−0.0809	0.392	0.362	0.143	0.343
	(0.166)	(0.162)	(0.234)	(0.237)	(0.394)	(0.403)	(0.433)	(0.437)
Observations	1010	975	1015	979	1015	979	1015	979
*R* ^2^	0.045	0.122	0.034	0.047	0.030	0.040	0.031	0.043

*Note*: Robust standard errors in parentheses.

* *p* < 0.10.

** *p* < 0.05.

*** *p* < 0.01.

**Table 7:** Generalized trust, experimental trust, trustworthiness and expected trustworthiness (South vs. rest of Italy)

	GTQ		Trust		Trustworthiness		Expected trustworthiness	
	(1)	(2)	(3)	(4)	(5)	(6)	(7)	(8)
South	−0.230	−0.260	−0.107	−0.0501	−0.776**	−0.924**	0.126	−0.0571
	(0.178)	(0.177)	(0.232)	(0.238)	(0.391)	(0.394)	(0.448)	(0.453)
Openness		−0.0819		−0.210		−0.385		−0.428*
		(0.0949)		(0.133)		(0.242)		(0.252)
Conscientiousness		−0.211*		−0.0585		−0.169		−0.0417
		(0.113)		(0.155)		(0.288)		(0.304)
Extraversion		0.487***		−0.154		0.00200		0.630***
		(0.0892)		(0.127)		(0.207)		(0.215)
Agreeableness		0.232**		0.113		0.291		0.308
		(0.118)		(0.152)		(0.283)		(0.300)
Neuroticism		−0.451***		−0.261**		−0.246		−0.131
		(0.0863)		(0.114)		(0.196)		(0.210)
Controls	Yes	Yes	Yes	Yes	Yes	Yes	Yes	Yes
Observations	1010	975	1015	979	1015	979	1015	979
*R* ^2^	0.040	0.119	0.031	0.043	0.020	0.029	0.021	0.034

*Note*: Robust standard errors in parentheses.

* *p* < 0.10.

** *p* < 0.05.

*** *p* < 0.01.

With respect to the other preferences, we do not find any significant difference across macroareas (Table 8) or between the South and the rest of Italy (Table 9) in terms of altruism (Columns 1–2), cooperation (3–4), conditional cooperation (5–6), and risk propensity (7–8).

**Table 8:** Differences in altruism, cooperation, conditional cooperation and risk propensity by macroarea

	Altruism		Cooperation		Cond. cooperation		Risk propensity	
	(1)	(2)	(3)	(4)	(5)	(6)	(7)	(8)
North‐east	−0.247	−0.282	−0.246	−0.250	−0.000117	−0.0180	−0.0559	−0.107
	(0.215)	(0.213)	(0.268)	(0.269)	(0.0390)	(0.0397)	(0.150)	(0.151)
Center	−0.395*	−0.343	0.341	0.279	0.00601	0.0156	−0.0868	−0.0836
	(0.217)	(0.220)	(0.261)	(0.264)	(0.0380)	(0.0384)	(0.148)	(0.151)
South	−0.110	−0.0984	−0.0428	−0.0613	−0.00652	−0.0146	0.132	0.123
	(0.208)	(0.214)	(0.263)	(0.268)	(0.0370)	(0.0376)	(0.151)	(0.155)
Islands	0.359	0.408	0.362	0.355	−0.0130	−0.000382	0.0818	0.0320
	(0.290)	(0.291)	(0.343)	(0.347)	(0.0494)	(0.0496)	(0.192)	(0.193)
Openness		0.0561		0.0905		0.0147		0.0906
		(0.104)		(0.126)		(0.0178)		(0.0742)
Conscientiousness		−0.205*		0.219		−0.00347		−0.0925
		(0.122)		(0.153)		(0.0235)		(0.0856)
Extraversion		0.151		−0.127		−0.0197		0.0678
		(0.0972)		(0.127)		(0.0168)		(0.0690)
Agreeableness		−0.111		0.0301		0.0291		0.0930
		(0.115)		(0.146)		(0.0206)		(0.0861)
Neuroticism		−0.0270		−0.129		−0.0275*		−0.0267
		(0.0906)		(0.113)		(0.0161)		(0.0641)
Controls	Yes	Yes	Yes	Yes	Yes	Yes	Yes	Yes
Observations	1015	979	1015	979	1015	979	1015	979
*R* ^2^	0.033	0.039	0.047	0.059	0.067	0.075	0.027	0.035

*Note*: Robust standard errors in parentheses.

* *p* < 0.1.

** *p* < 0.05.

*** *p* < 0.01.

**Table 9:** Altruism, cooperation, conditional cooperation and risk propensity (South vs. rest of Italy)

	Altruism		Cooperation		Cond. cooperation		Risk propensity	
	(1)	(2)	(3)	(4)	(5)	(6)	(7)	(8)
South	−0.00621	−0.00821	−0.128	−0.129	−0.00610	−0.0144	0.155	0.164
	(0.178)	(0.185)	(0.228)	(0.234)	(0.0327)	(0.0335)	(0.129)	(0.132)
Openness		0.0596		0.0833		0.0141		0.0906
		(0.105)		(0.125)		(0.0178)		(0.0740)
Conscientiousness		−0.201		0.235		−0.00284		−0.0917
		(0.122)		(0.153)		(0.0235)		(0.0858)
Extraversion		0.160		−0.130		−0.0198		0.0702
		(0.0978)		(0.126)		(0.0168)		(0.0687)
Agreeableness		−0.123		0.0208		0.0286		0.0895
		(0.116)		(0.146)		(0.0207)		(0.0859)
Neuroticism		−0.0429		−0.149		−0.0281*		−0.0295
		(0.0909)		(0.114)		(0.0159)		(0.0638)
Controls	Yes	Yes	Yes	Yes	Yes	Yes	Yes	Yes
Observations	1015	979	1015	979	1015	979	1015	979
*R* ^2^	0.025	0.031	0.043	0.055	0.067	0.075	0.026	0.034

*Note*: Robust standard errors in parentheses.

* *p* < 0.1.

**p < 0.05.

***p < 0.01.

Neither of these results stems from the online nature of the experimental and survey setting adopted in Trustlab. Although both an internet connection and a device enabling online access are needed to take part in Trustlab, we find no evidence that this selection brings about systematic trends in the experimental outcomes. In fact, the North‐South gap in trustworthiness (and within‐country similarity with respect to other social preferences) is confirmed under Heckman's correction for selection into internet access (Heckman, 1979).^19^

In Table 10 we check for within‐country differences in social engagement through an ordered logit regression of the frequency of voluntary work (Columns 1–2 and 5–6) and of encounters with friends (Columns 3–4 and 7–8). Also in this case, we do not find evidence of lower levels of social engagement in the South. Conversely, Southerners tend to have more frequent social contacts^20^ (Table 10, Columns 3–4 and 7–8). However, these results do not imply that they are in general more given to cooperation. Since frequency of social contacts in our data includes friends, this variable is closer to the “bonding” rather than to the “bridging” feature of social capital (Putnam, 2000; Uslaner, 2002), with mainly the latter capturing trust in (and cooperation with) *unknown* persons and being associated with better economic performance (e.g., Tabellini, 2008).

**Table 10:** Differences in social engagement by macroarea

	Frequency of							
	voluntary work		encounters with friends		voluntary work		encounters with friends	
	(1)	(2)	(3)	(4)	(5)	(6)	(7)	(8)
North‐east	0.195	0.162	−0.0999	−0.0790				
	(0.189)	(0.191)	(0.180)	(0.181)				
Center	−0.320*	−0.294	0.0984	0.0574				
	(0.188)	(0.197)	(0.172)	(0.174)				
South	0.0635	−0.0647	0.651***	0.624***	0.0262	−0.0806	0.553***	0.550***
	(0.176)	(0.178)	(0.165)	(0.173)	(0.149)	(0.154)	(0.145)	(0.153)
Islands	0.487**	0.342	0.622***	0.500**				
	(0.219)	(0.226)	(0.227)	(0.228)				
Openness		0.322***		0.208**		0.331***		0.208**
		(0.0949)		(0.0891)		(0.0941)		(0.0894)
Conscientiousness		0.118		0.0474		0.119		0.0560
		(0.108)		(0.108)		(0.108)		(0.108)
Extraversion		0.291***		0.422***		0.298***		0.419***
		(0.0855)		(0.0845)		(0.0855)		(0.0845)
Agreeableness		0.102		0.0549		0.0998		0.0541
		(0.110)		(0.103)		(0.108)		(0.102)
Neuroticism		−0.0370		−0.265***		−0.0403		−0.281***
		(0.0842)		(0.0800)		(0.0836)		(0.0799)
Controls	Yes	Yes	Yes	Yes	Yes	Yes	Yes	Yes
Observations	1015	979	1015	979	1015	979	1015	979
Pseudo *R* ^2^	0.0367	0.0556	0.0305	0.0563	0.0312	0.0521	0.0266	0.0538

*Note*: Ordered logit estimates. Robust standard errors in parentheses.

* *p* < 0.1.

** *p* < 0.05.

*** *p* < 0.01.

To understand whether the findings on trust and trustworthiness conceal North‐South differences in other preferences, we re‐estimate the previous models of Table 7 (Column 4 and 6) controlling for respondent's behavior in other games. Similar to previous studies (Ashraf et al., 2006; Chaudhuri & Gangadharan, 2007; Sapienza et al., 2013), the respondent's choices in the role of trustor seem motivated by unconditional kindness and cooperation (Table 11). Since the receiver's behavior is also positive and significant whereas expected trustworthiness is not, it is likely that trustors formed expectations of reciprocity by extrapolating the expected behavior of their opponents from their own (Sapienza et al., 2013).^21^

**Table 11:** The rationales of trust

	Trust							
	(1)	(2)	(3)	(4)	(5)	(6)	(7)	(8)
South	0.135	0.0416	−0.0463	0.00559	−0.0538	−0.0683	0.0871	0.0644
	(0.228)	(0.230)	(0.225)	(0.220)	(0.239)	(0.240)	(0.211)	(0.221)
Trustworthiness	0.200*						0.102*	0.127*
	(0.0172)						(0.0207)	(0.0214)
Expected trustworthiness		0.149*					0.0327	0.0365**
		(0.0177)					(0.0209)	(0.0213)
Altruism			0.472*				0.227*	0.335*
			(0.0388)				(0.0462)	(0.0453)
Cooperation				0.433*			0.306*	
				(0.0322)			(0.0349)	
Cond. cooperation					−0.253			−0.0823
					(0.250)			(0.219)
Risk propensity						0.111**	0.00221	0.0377
						(0.0649)	(0.0542)	(0.0568)
Controls	Yes	Yes	Yes	Yes	Yes	Yes	Yes	Yes
PTs	Yes	Yes	Yes	Yes	Yes	Yes	Yes	Yes
Observations	979	979	979	979	979	979	979	979
*R* ^2^	0.164	0.118	0.175	0.213	0.044	0.046	0.305	0.233

*Note*: Robust standard errors in parentheses.

* *p* < 0.1.

** *p* < 0.05.

***p < 0.01.

Conversely, the North‐South gap in trustworthiness is confirmed (and is even larger) when we control for player's behavior in other games (Table 12). With the exception of risk propensity, the additional variables are all statistically significant.^22^ This evidence is consistent with previous studies showing that trustworthiness can be motivated by other‐regarding conditional and unconditional preferences (Ashraf et al., 2006; Cox, 2004). Importantly, the inclusion of these preferences leads to a remarkable increase in the goodness of fit.^23^ This suggests that a significant portion of the variation in trust and trustworthiness is explained by respondents' behavior in other games rather than their observed (and likely unobserved) individual characteristics.^24^

**Table 12:** The rationales of trustworthiness

	Trustworthiness							
	(1)	(2)	(3)	(4)	(5)	(6)	(7)	(8)
South	−0.892**	−0.896***	−0.917**	−0.862**	−0.922**	−0.944**	−0.854***	−0.861***
	(0.375)	(0.341)	(0.371)	(0.383)	(0.394)	(0.396)	(0.327)	(0.328)
Trust	0.632**						0.297***	0.333***
	(0.0615)						(0.0603)	(0.0559)
Expected trustworthiness		0.486**					0.388***	0.393***
		(0.0398)					(0.0396)	(0.0398)
Altruism			0.810**				0.294***	0.331***
			(0.0915)				(0.0856)	(0.0875)
Cooperation				0.479**			0.114**	
				(0.0631)			(0.0560)	
Cond. cooperation					0.130			0.706*
					(0.478)			(0.371)
Risk propensity						0.121	−0.0928	−0.0658
						(0.118)	(0.0920)	(0.0929)
Controls	Yes	Yes	Yes	Yes	Yes	Yes	Yes	Yes
PTs	Yes	Yes	Yes	Yes	Yes	Yes	Yes	Yes
Observations	979	979	979	979	979	979	979	979
*R* ^2^	0.152	0.287	0.153	0.096	0.029	0.030	0.353	0.353

*Note*: Robust standard errors in parentheses.

* *p* < 0.10.

** *p* < 0.05.

*** *p* < 0.01.

The lack of a significant North‐South divide in trust and in expected trustworthiness also suggests that Southern‐Italian trustors fail to anticipate the lower reciprocity levels in their macroarea. This result is confirmed when calculating the payoff‐maximizing transfer on the basis of the empirical distribution of return choices in the trustor's macroarea. More specifically, we computed the median amount returned by the trustee for each hypothetical transfer and in each macroarea. We then calculated the corresponding theoretical payoffs of the trustor. These payoffs appear to be lower in the South than in the Northern macroareas, especially for higher transfers (Figure A4 in Appendix A). While we further discuss this finding in the next section, it is important to note here that in most Italian macroareas there is only one profit‐maximizing transfer (i.e., €10), whereas in the South trustors would equally maximize profits by sending €5, €9, or €10. However, the presence of unique versus multiple maximizing transfers does not translate into real differences in trustor's choices, which appear to be distributed in a similar (bimodal) way across macroareas (Figure A5 in Appendix A).^25^

This last result suggests that the nonresult for the North‐South gap in trust could be driven by non‐Southern trustors sending less than what would be optimal according to the trustworthiness levels in their macroarea. Their transfers, instead, appear more consistent with the trustworthiness patterns we observe in the South.

Overall these findings suggest that there is no evidence of a systematic gap in trust and cooperation between North and South as shown in previous studies. In addition, the lack of North‐South differences in expected trustworthiness in our data contrasts with the evidence from nonrepresentative data in Bigoni et al. (2019), who show that the cooperation gap they found in their previous study (Bigoni et al., 2016) is due to the pessimistic beliefs Southerners have about their own cooperativeness compared to beliefs of Northerners about other Northerners' cooperativeness. Our countrywide lab‐experiment suggests, instead, that the North‐South gap in social capital is preference‐ and not belief‐based, and lies only in *one* particular dimension, that is, reciprocity.

Apart from differences in sample representativeness, another possible explanation for our divergent results is that the beliefs‐elicitation method in Bigoni et al. (2019) rests on an explicit priming of the “North” versus “South” categories, which could lead to an overestimation of otherwise less‐stereotyped beliefs about Southerners' level of cooperation. The elicitation of the subject's beliefs about others' trustworthiness in our experiment is, instead, not conditional on the geographical origins of the counterpart, and it might therefore, be interpreted as a more conservative estimate of expected reciprocity. Lastly, whereas beliefs‐elicitation is incentivized in Bigoni et al. (2019), it is not in Trustlab.

## 6 UNDERSTANDING THE NORTH‐SOUTH GAP IN TRUSTWORTHINESS

In this section, we analyze trustee's reciprocity by exploiting the strategy method, which allows us to understand how receivers condition their choices on the basis of their opponent's hypothetical choices. With this information, we also test whether the North‐South gap is driven by differences in conditional reciprocity when expected profits from the dominant strategy (“do not reciprocate”) increase.

**Figure 5:**
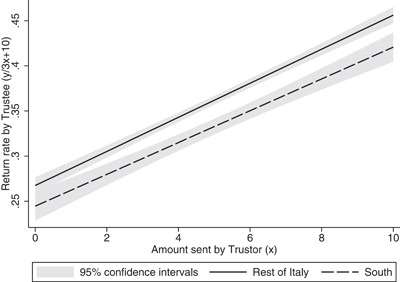
The North‐South gap in conditional reciprocity (trust game)

Figure 5 plots the trustee's return rates as a function of the 11 hypothetical transfers. As in previous studies (Ashraf et al., 2006; Bellemare & Kröger, 2007; Bornhorst et al., 2010; Schotter & Sopher, 2006), the upward sloping curve confirms that reciprocity is the driving force of trustworthiness. With respect to the North‐South gap, both Southerners and Northerners are “conditional reciprocators,” since the return rate on average increases in proportion to the amount that they receive. However, Southerners tend to reciprocate less than Northerners when transfers are larger than 40% of the trustor's endowment.

We check for the significance of this difference through an OLS regression controlling for individual‐level characteristics and by looking at behavior in other games. More specifically, we treat the trustee's choices in the response vector as separate rounds of a trust game, and regress the amount returned on the hypothetical transfer by estimating Equation (2)

(2) $$Y_{ijt}=\alpha_{0}+\alpha M_{j=South}+\underset{k}{\sum }\beta_{k}X_{ik}+\gamma Send_{t}+\epsilon_{ijt}$$

where $Y_{ijt}$ is the amount the respondent i living in macroarea j decides to return conditional on the hypothetical transfer t of the trustor (t={0,…,10}), and Send is a variable capturing the increase in the transfer. All other controls are the same as in Table 12 (Column 7), including the other experimental measures. We consider the South dummy, $M_{j}$, instead of the four dummies for the macroareas (results are similar in both specifications^26^), equal to one if respondent i lives in macroarea j=South and zero otherwise. The coefficient γ can be interpreted as a measure of conditional reciprocity, that is, how much receiver's decisions depend on the size of the senders' transfer. Since we have eleven data points *per* respondent (for a total of 10,769 observations), we clustered standard errors at the individual level. $\alpha_{0}$ is a constant term, while $\epsilon_{ijt}$ is an error term. In alternative specifications the South macroarea dummy is interacted with the variable Send, while also a quadratic Send term is introduced and interacted with the South macroarea dummy.

Results in Table 13 confirm the diverging path in conditional reciprocity as highlighted in Figure 5. Trustees are, on average, conditional reciprocators since their return choices significantly depend on the amount sent by the trustor (Column 1). As expected, Southerners return, on average, less than Northerners, confirming the previous results. However, the interaction between the trustor's transfer and the South dummy is negative and significant, suggesting that the North‐South gap in reciprocity widens as the transfer increases (Column 2). The same effect is also found when allowing for a nonlinear relationship between trustees' decisions and trustor's transfers (Columns 3 and 4).

**Table 13:** The North‐South divide in conditional reciprocity (trust game)

	Reciprocity			
	(1)	(2)	(3)	(4)
South	−0.854***	−0.0579	−0.854***	−0.212
	(0.322)	(0.275)	(0.322)	(0.274)
Send	1.475***	1.510***	1.450***	1.462***
	(0.0274)	(0.0302)	(0.0344)	(0.0388)
Send X South		−0.159**		−0.0568
		(0.0702)		(0.0842)
Send squared			0.00254	0.00475
			(0.00254)	(0.00296)
Send squared X South				−0.0102*
				(0.00562)
Trust	0.297***	0.297***	0.297***	0.297***
	(0.0594)	(0.0594)	(0.0594)	(0.0594)
Cooperation	0.114**	0.114**	0.114**	0.114**
	(0.0551)	(0.0552)	(0.0552)	(0.0552)
Expected trustworthiness	0.388***	0.388***	0.388***	0.388***
	(0.0390)	(0.0390)	(0.0390)	(0.0390)
Altruism	0.294***	0.294***	0.294***	0.294***
	(0.0844)	(0.0844)	(0.0844)	(0.0844)
Risk propensity	−0.0928	−0.0928	−0.0928	−0.0928
	(0.0907)	(0.0907)	(0.0907)	(0.0907)
Controls	Yes	Yes	Yes	Yes
PTs	Yes	Yes	Yes	Yes
Observations	10,769	10,769	10,769	10,769
*R* ^2^	0.543	0.544	0.543	0.544

*Note*: Robust standard errors in parentheses, clustered at individual level.

* *p* < 0.10.

** *p* < 0.05.

*** *p* < 0.01.

**Figure 6:**
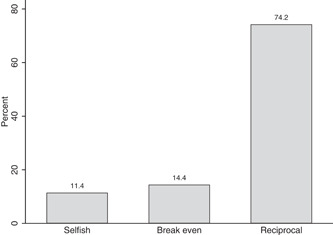
Distribution of types of trustworthiness choices

**Figure 7:**
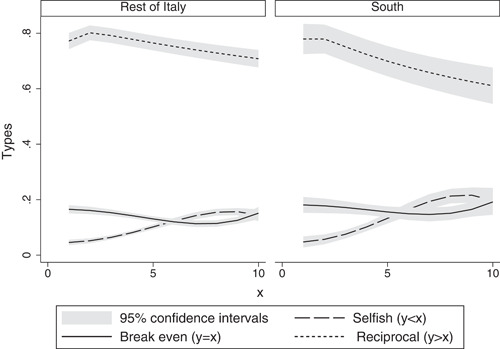
The evolution of reciprocal strategies over the amount transferred

We also analyze conditional reciprocity by classifying subjects according to the amount they return for each possible transfer. As Figure 6 shows, in our sample we classify 11% of the trustee's choices as “selfish,” 14% as “break even” and 74% as “reciprocal” when they are, respectively, below, equal to or above the hypothetical transfer. In other words, selfish choices provide trustors with negative returns on investment, while break even and reciprocal choices imply, respectively, zero or positive returns. Figure 7 shows how the number of selfish (reciprocal) choices starts increasing (decreasing) for transfers larger than 40% of the trustors' endowment (e.g., €4). This pattern is stronger in the South than in the rest of Italy.

We then estimate the determinants of the probability of playing each strategy. Results are reported in Table A6 in Appendix A, and show that the likelihood of selfish (reciprocal) choices increases (decreases) when the amount at stake gets larger. However, Southern regions are significantly different from the others only in reciprocal choices, which are less likely in the case of Southerners (Column 5). Consistent with results in Table 13, the North‐South gap in reciprocity widens when transfers increase (Table A6 in Appendix A, column 6), with Southerners rewarding trustors less often than Northerners, as doing so generates larger profits.

These results highlight a new dimension underlying the North‐South gap in social capital that has not been analyzed in previous studies. The higher the temptation to defect, that is the larger the amount at stake, the more likely are Southerners (as opposed to Northerners) to reciprocate less to maximize their own benefits.

To check whether this behavior is also there in other scenarios replicating a social‐dilemma, we analyze contributions in the PGG conditional on the average amount contributed by group members. If the North‐South differences are driven by a decrease in Southerners' reciprocity when the selfish strategy is more profitable, we should observe the same pattern for conditional cooperation when group contributions increase. Figure A6 in Appendix A shows that when group contributions are above 60% of the endowment both Southerners and Northerners contribute less than 60%, but contributions are lower in the South than in the North.

Similarly, regression results for the PGG in Table A7 in Appendix A mirror those for the TG in Table 13, showing that subjects tend to condition their contribution on the behavior of their group members, though non linearly (Column 3). However, while *on average* there is no significant North‐South gap in cooperation, an increase in the expected payoffs from free‐riding leads Southerners to contribute less than Northerners (Column 4).

Summarizing, these findings suggest that as long as there is little to lose, Southerners and Northerners cooperate and reciprocate in the same manner. However, when the amount at stake increases, preferences for reciprocity and cooperation start diverging, with Southerners defecting more often than Northerners. The fact that this behavior is consistent both in the TG and PGG suggests that Southerners obey a social norm that prevents them from rewarding highly prosocial acts.^27^

## 7 OTHER EXPLANATIONS FOR THE TRUSTWORTHINESS GAP

According to Putnam, social capital refers to “connections among individuals—social networks and the norms of reciprocity and trustworthiness that arise from them” (Putnam, 2000, p. 19). To assess whether the trustworthiness gap can be explained by North‐South differences in the “Putnamian” dimensions of social capital, we add to the main trustworthiness regression the individual‐level measures of civic and social engagement: for example, voluntary work, connectedness with neighbors, and participation in the last political elections. Results reported in Table A14 in Appendix A show that these facets of social capital cannot account for the North‐South gap in trustworthiness. In the following sections, we explore other potential explanations for the trustworthiness gap: betrayal aversion, strength of family ties and migration.

### 7.1 The Italian Trustlab follow‐up

In May and June 2018, additional survey modules were administered on the original Italian Trustlab sample with the purpose of measuring other preferences and characteristics of respondents that have been shown to be important explanations for the North‐South gap. The follow‐up survey collected information primarily aimed at: (i) disentangling aversion to social risk from aversion to natural risk (betrayal aversion); (ii) assessing the extent to which people in the sample respond to “familistic” norms (strength of family ties); (iii) reconstructing the residential history of respondents; and (iv) finding patterns of intergenerational norms transmission.^28^

Because of an attrition rate of about 25% of the sample in our main estimates, we include, in the following analysis, a supplementary sample, which enables us to increase statistical power for testing the new hypotheses.^29^ Since the analyses in the following sections rely on the largest set of respondents (those in the extended sample who also participated in the follow‐up), we restore representativeness by creating weights to adjust the demographic composition of the extended sample (in terms of gender and age) to that of the Italian population as it was in 2017. Moreover, we control for the residual heterogeneity of the supplementary sample by augmenting our models with a dummy variable, taking value one for respondents who were not part of the representative sample. Importantly, the inclusion of the extended sample does not alter the results shown in the previous tables, thereby underlining the validity of the estimates. In Tables A15 and A16 in Appendix A we check the consistency of some of the main results presented so far by re‐estimating models on a sample inclusive of the supplement. Statistical significance of the main variables' coefficients is, if different from previous models, higher. In general, the magnitude of re‐estimated coefficients is slightly larger, while the control variable marking the supplementary respondents is always far from approaching significance. Notice also that re‐estimation of all the models in previous tables provides almost equal results (available upon request).

### 7.2 Betrayal aversion

In comparison with Northern Italians, Italians in the South have been shown to be more averse to betrayal: that is they dislike risk when risk relates to human behavior rather than to nature (Bigoni et al., 2019). Since the literature has shown that betrayal aversion is mainly associated with trust (and not trustworthiness), we could, in principle, rule out aversion to betrayal as a possible explanation for the observed South‐North gap in reciprocity.

However, our results could be due to unobserved differences in the way in which trustees *internalize* the potential cost of betrayal when it comes to trust. Such internalization may emerge more clearly when individuals play both roles in a trust game (as the participants in Trustlab do). Taking betrayal aversion into account, we would expect higher reciprocity in the South, provided that Southerners are systematically more betrayal‐averse *and* systematically more likely to internalize the trustor's disutility from expected betrayal than non‐Southerners.

Our evidence showing lower reciprocity in the South suggests that this is not the case. This is probably because the internalization of trustor's aversion to betrayal is less likely to occur in the South, or because betrayal aversion is not systematically different across Italian macroareas. To shed light on the role of betrayal aversion, we nonetheless check whether the North‐South gap in reciprocity mirrors an underlying gap in aversion to betrayal, and whether the former narrows when controlling for the latter.

To derive a measure of betrayal aversion, in the follow‐up study, we performed a survey‐based task to measure whether respondents are more willing to take on risk when such risk derives from nature rather than from another person's actions.^30^ More specifically, we adopt the vignette‐based approach as in Cubitt et al. (2017), who rely on a hypothetical scenario where people need to take a taxi from the airport to the city center, and they have to choose between two taxi companies: one charging a fixed fee and the other using the taximeter. While the first company charges the same amount (€12) however long the journey (*safe company*), the price charged by the second company (*risky company*) is uncertain: 1/5 probability of €16, and 4/5 probability of €8. The two vignettes differ in terms of the risk faced by the respondents when making their choice between the safe and the risky company: in one case the risk stems from weather conditions (*natural risk*), while in the other case the risk relates to human behavior, that is, the taxi driver (*social risk*).^31^

We use the same parametrization as in Cubitt et al. (2017) so that, with an expected cost of €9.60, a risk neutral, profit‐maximizing agent would always choose the risky company. Risk‐averse respondents might choose the safe company in the natural risk vignette, even though it is more expensive. Thus, betrayal aversion would make respondents more likely to choose the safe company in the social‐risk vignette than in the natural‐risk vignette (refer to Appendix C for the text of the two vignettes).^32^

Consistent with Bigoni et al. (2019),^33^ we find an overall prevalence of betrayal aversion in Italy. As in Cubitt et al. (2017), the share of respondents who chose the safe option in the first vignette is significantly higher when the vignette depicts social rather than natural risk. The difference is at least as great as 7.7 percentage points and significant in each macroarea, reaching a peak in North‐eastern Italy (Table A17 in Appendix A). To test if betrayal aversion significantly differs across macroareas, we regress an indicator variable for the safe option (*Safe choice*) on the South dummy, a dummy variable equal to one for the social‐risk scenario (SR) and their interaction.^34^ Regression results (Table A18 in Appendix A) show that there is no significant gap in betrayal aversion between the South and other macroareas.

The combination of answers in both vignettes allows us to categorize four different types of individuals. The “risk averse” types are those choosing the safe option in both vignettes; at the other extreme we have what we have termed “risk lovers,” that is, the respondents with a preference for risk irrespective of the situation they face. In the middle, there are the “principled trustful” (Fetchenhauer & Dunning, 2012), respondents who tend to accept risk only insofar as such risk stems from social interactions, but who avoid it when it comes from nature. Lastly, we categorize respondents as “betrayal averse” if they opt for the safe option when exposed to social risk, but they choose the risky option when facing natural risk. In our sample, most individuals are risk averse (about 58%), while only about 19% are betrayal averse; risk lovers and principled trustful individuals are rather few, respectively about 15% and 8% (Figure A7 in Appendix A). Consistent with results in Table A18 in Appendix A, the distribution of types does not vary significantly by macroareas (Figure A8 in Appendix A). None of these types shows statistically significant correlations with trustworthiness (Table A19 in Appendix A). Betrayal averse and (to a lesser extent) risk averse individuals appear, on average, to be more trustworthy than risk lovers and principled trustful ones, probably because the risk averse subjects, when playing as trustors, are more likely to internalize the social risk embedded in the decision to trust.^35^

Overall this evidence suggests that betrayal aversion does not explain the North‐South gap in reciprocity.

### 7.3 Family ties

A potential explanation for the trustworthiness gap hinges on the geographical differences in the strength of family ties. In collectivistic societies, most socioeconomic transactions rely on mutual obligations among known individuals, where the risk of being cheated is mitigated by informal commitment‐devices such as monitoring and sanctioning (see Yamagishi & Yamagishi, 1994; Yamagishi et al., 1998).^36^ Trust in unknown persons is, therefore, endangered as strong and stable relations, by decreasing social risk, provide an “assurance” of mutual cooperation (Yamagishi & Yamagishi, 1994). Sanctioning and monitoring are, of course, more efficiently carried out among small groups of known persons. Therefore, lower trustworthiness should emerge more easily when dealing with strangers, as deviations from the socially optimal equilibrium are less promptly discovered (and punished) in these kinds of transactions. Thus, strong family ties should negatively affect both trust and trustworthiness in anonymous transactions, like those mimicked by the TG. Consistent with this hypothesis, Alesina and Giuliano (2014) find a negative relationship between strong family ties (measured through survey questions) and generalized trust. Similarly, Ermisch and Gambetta (2010), in an experimental setting, find that strong family ties—measured through the self‐reported frequency of contacts with relatives—predict significantly lower trust.

As in Bertrand and Schoar (2006), Alesina and Giuliano (2010), and Alesina and Giuliano (2014), in the follow‐up study we measure the respondents' family ties by relying on three questions on the importance of family, as asked in the EVS. The first question asks respondents how important is family in their life (answers range from “1—Not at all important” to “4—Very important”). Then, respondents state their agreement with one of two statements about the parent‐child relationship and their responsibilities to each other, that is, “1—there's no duty to respect and love parents who misbehave” or “2—parents should be loved and respected in any circumstances.” The last question is about responsibilities of parents towards children, that is, “1—parents should not pursue children's well‐being if this implies giving up their own's,” or “2—children deserve the best irrespective of sacrifices in which parents might incur.” We aggregate answers to these questions through a principal component analysis and consider the first extracted component as a proxy for the strength of family ties.^37^

Family ties appear stronger in Southern Italy and in the Islands (Sicily and Sardinia), while they are weaker in the Northern regions of Italy (Figure A9 in Appendix A).^38^ The North‐South gap in family ties is also confirmed when regressing family ties (*family ties [PCA]*) on the South dummy and other controls (Table A20 in Appendix A). To test whether the North‐South difference in family ties accounts for the North‐South gap in reciprocity we add family ties to the regressions of trustworthiness on the South dummy and controls. While, as expected, strong ties negatively predict reciprocity, the South dummy remains negative and significant, thereby suggesting that the strength of family ties is not the main explanation for the North‐South divide in trustworthiness (Tables A21 and A22 in Appendix A).

### 7.4 Migration

Another possible explanation for the trustworthiness gap is the self‐selection of emigrants. If Southerners moving to Northern regions have on average lower level of trustworthiness, the estimated North‐South gap in reciprocity would be a lower bound of the real gap. A major concern arises, instead, if Southerners with greater civic and human capital move to the North, for instance because they are attracted by better job perspectives or because they feel uncomfortable with the uncooperative social norms in the region of origin. Self‐selection of emigrants implies that, when the most prosocial South‐Italian citizens emigrate, the regions of origin are left with low‐cooperative individuals and hence experience a “civicness drain” (Casari et al., 2018).

To assess the role of migration, we collect information about respondents' residential history, that is, where they were born, where they spent most of their life until age 16, and the province of origin of their parents. In this way, we are able to identify respondents who emigrated to the region in which they currently live and, also, the stage of life in which migration occurred. Descriptive statistics show that 14% of respondents migrated to the current macroarea, with most of them moving from the South (46%); the most frequent migration route is from the South to the North, especially to the North‐west of Italy (Figure A10 in Appendix A). Among respondents born in the South, trustworthiness tends to be higher in respondents who currently live in Northern regions, than for those who live in other regions or remained in the South (Figure A11 in Appendix A); yet these differences fell only marginally short of significance.

We also re‐estimate our preferred trustworthiness regression including two dummy variables for individuals who moved from the South to the North and for other migration patterns (the omitted category is composed of nonemigrants). In alternative specifications we consider, too, migration at different stages of life, and include indicators for specific migration routes across macroareas and periods of life (before or after age 16). Results show that emigrants are not statistically different in trustworthiness from nonemigrants, while the North‐South gap in trustworthiness remains statistically significant in all specifications (Table A23 in Appendix A). Thus, self‐selection of emigrants or learning of social norms do not seem to account for low reciprocity in the South.^39^

### 7.5 Intergenerational transmission

The observed gap in trustworthiness could also be driven by the intergenerational transmission of social norms and values, with parents from the South passing on to their children norms of behavior based on low reciprocity. If social norms are inherited from parents in childhood and change only slowly thereafter (Bisin & Verdier, 2001; Dohmen et al., 2011; Guiso et al., 2008; Giulietti et al., 2016), respondents with a Southern Italian parent should display lower trustworthiness than those with a parent from a different macroarea, regardless of their current residence.

To test this hypothesis, we re‐estimate the trustworthiness regression replacing the South dummy with an indicator for respondents living in the North. We also add a dummy variable for respondents having at the least one parent from the South, who represent 39.8% of our sample. To adjust for residential history, in additional specifications we control for migration from South to North and other migration patterns (at any age). Regression results (Table A24 in Appendix A) document that living in the North is associated with higher trustworthiness (Column 1); this positive effect is, however, absorbed by the Southern origins of parents, which pulls the data in the opposite direction (Column 2). Interestingly, living in the North slightly counterbalances the negative effect of parental origins for trustworthiness (Column 3), regardless of respondents' migration decisions, which—as previously shown—do not play a significant role (Column 4). The fraction of respondents living in the North with at least one parent from the South is 12.2%.

Overall this evidence suggests that the lower trustworthiness of non‐Northerners could be a result of the intergenerational transmission of norms, prescribing low reciprocity. This might, then, be moderated by a prolonged exposure to the highly reciprocal contexts of Northern Italian regions. Such moderation occurs independently of respondents' migration patterns, thereby suggesting that it is parental attitudes to a high‐trustworthiness environment, rather than the exposure of their children to these contexts, that offsets the intergenerational transmission of low‐trustworthiness norms.

## 8 CONCLUSIONS

This paper offers novel results on the North‐South gap in the Italian social capital. We find that Southern Italians are *not* statistically different from citizens residing in other macroareas with respect to *universal* social preferences, namely generalized trust, beliefs about others' trustworthiness, cooperation, altruism, and risk preferences. Furthermore, no robust differences in survey‐measured trust, civicness and social participation are found. The only statistically significant gap emerges in reciprocity: average trustworthiness is about 10 percentage points lower in the South than in the rest of Italy. While both Northerners and Southerners are conditional reciprocators, the latter tend to return less than the former when the temptation to deviate from the socially‐optimal equilibrium increases (i.e., the trustor's transfer gets larger). Higher trustworthiness in non‐Southern regions is, nevertheless, not anticipated by trustors, who—by transferring an amount below the profit‐maximizing one—fail to reap the benefits of the high reciprocity of their area.

Through follow‐up data, we also show that the gap in trustworthiness is not due to participants' differences in betrayal aversion or to the strength of family ties, while the self‐selection of emigrants does not seem to drive our findings. Finally, none of our socio‐demographic and economic controls, including personality traits, play a mediating role. Our evidence, instead, documents that—independently of current residence—the North‐South gap in trustworthiness originates from the Southern origin of respondents' parents. This effect is partly compensated for by living in the North, but it is not explained by migration choices (at any age) of the respondents. This last finding reveals that the learning of high‐reciprocity norms is a long‐term process. It might take more than one generation to be accomplished.

Our results could be seen as complementary with those provided by Bigoni et al. (2016) and Bigoni et al. (2019). In the latter, North‐South Italians show different prosocial preferences when stereotyped intergroup identities or the “local,” narrow identity of the county is made salient. Thus, as long as agents know their counterparts' geographic origin and/or can form stereotyped beliefs on them, there might be room for policy: economic convergence could be achieved by stimulating in‐group trust and cooperation in the South or promoting optimistic beliefs about Southerners' trustworthiness (Bigoni et al., 2016). However, our results suggest that when geographic identities are not made salient, preferences do not differ that much: apart from reciprocity, Italians share a common *generalized* preference for trust and cooperation. Policies aiming at achieving economic convergence, therefore, need to be specific and should target generalized reciprocity, while promoting activities aimed at building a broad sense of identity, which has to trespass the boundaries of one's own county or region. Future studies could further investigate whether differences in identity or “sense of belonging,” properly defined and measured through specific tasks not included in this study, might explain the North‐South gap in reciprocity.

Overall our findings suggest that perhaps too much emphasis has been put on the *cultural* roots of the economic disparities plaguing Italy since unification in the 1860s. This study provides experimental and survey‐based evidence suggesting that, in most social‐capital dimensions, the preferences for trust in (and cooperation with) unknown persons on the part of Southern Italians are statistically indistinguishable from those of Italians living in other areas.

So, was Banfield right? A closer look into the Banfield's and Putnam's hypotheses carried out in Bigoni et al. (2016) suggests that none of the two find empirical support. First, the morality problem of Southerners identified by Banfield, and operationalized by the authors as a concern towards equity versus efficiency, does not explain much of the North‐South gap in cooperation found in the data. Second, the proxies for social capital used by the authors to test the Putnam's hypothesis do not seem to account for the differential patterns of in‐group cooperation across the locations where the experiments were carried out. The authors, thus, argue that the root of the North‐South gap in cooperation might lie in the role played by “preferences, expectations and social norms in shaping the differential ability to cooperate that we observe across Italy” (Bigoni et al., 2016, p. 1338), and suggest that conditional cooperation and betrayal aversion could account for differences in preferences and expectations.

Our results, on the contrary, highlight that Southerners do not show higher aversion to the social risk of being cheated, and do not display lower conditional cooperation with a generalized transaction partner; moreover, they tend to engage in more social interactions than Italians residing in other macroareas. Our results also suggest that the North‐South gap in trust, altruism and cooperation found in previous studies can disappear when no clue about the counterpart's origin is provided, and hence North and South Italians have similar levels of universal prosociality. These findings, jointly with the lack of predictive power of amoral familism shown by Bigoni et al. (2016), therefore imply that neither Banfield nor Putnam was right.

The same degree of universal prosociality we observe today can well be the result of a convergence process started centuries ago, with the Italian unification; a process which, except for trustworthiness, looks complete in many aspects of social capital. However, if Italian regions historically managed to converge in most of our experimental and survey‐based measures, they did not do so in terms of economic performance. In this regard, while not directly assessing the link between universal prosociality and growth, this paper nonetheless contributes to the debate on North‐South economic divide by highlighting that the reasons of the uneven economic outcomes of Italian macroareas might not necessary be cultural or preference‐based; or, at very last, that these reasons are not to be searched for in the lower innate propensity of Southerners to cooperate with strangers.

It may also be that universal prosociality does not harness economic growth, while parochial prosociality does. While this could be a promising line of research for future studies, it has to be considered that the previous literature on this topic has suggested rather the opposite: universal prosociality, measured as “generalized trust” or “generalized morality,” do matter for economic growth (e.g., Algan & Cahuc, 2010; Tabellini, 2010). However, differently from the experimentally validated measures used in this paper, this literature is mostly focused on nonincentivized survey measures of values and preferences, which are not particularly helpful in disentangling parochial versus universal attitudes towards cooperation. For instance, the GTQ used in many of these studies asks how much “most people” can be trusted, and therefore do not capture entirely what the specific target “most people” actually reminds of to different groups of people (Delhey et al., 2011, 2014). For instance, “most people” in the North may elicit different groups of people than those it may elicit in the South, thereby inducing measurement error or unobserved heterogeneity when estimating the effect of trust on economic performance.

An alternative interpretation to this mismatch between economic convergence and convergence in universal prosociality is that the North‐South economic divide could be a result of bad economic policies implemented over time at a national and subnational level (Beraldo, 2010; Daniele & Malanima, 2011; Felice, 2013). In facts, statistics on other factors related to growth reveal that national or international policy‐makers need also to guard against other gaps than those being of cultural nature. For instance, in spite of higher public spending on education in the South than in the North (respectively 6% vs. 2.7% of GDP), educational attainments are still dramatically different across Italian macroareas. Previous studies have, as we have seen, concluded that the “questione meridionale” (the Southern problem) is not just an economic problem, but also a cultural issue. The aforementioned gap in human capital—along with our results—suggests, instead, that narrowing differentials in social capital (to the extent that these exist) would not be sufficient to bring about change were educational outcomes not also equalized.

## Supporting information

Supplementary Information

## Footnotes

1 The experimental instructions clearly state that all participants in the session were born in the province or in the region where the experiment was conducted.

2 Concerns related to endogenous selection into the on‐line Trustlab platform are dealt with in Section 5 and Appendix B. Experimental instructions and the questionnaire are reported in Supporting Information Materials in Appendix C.

3 The institutions included in the Trustlab network (and the respective representatives) are Science Po Paris (Yann Algan), the Korean Development Institute (Soonhee Kim), the Government of Slovenia, Brown University (Louis Putterman), Kiel University (Gianluca Grimalda and Ulrich Schmidt), Bocconi University (Arnstein Aassve), the London School of Economics (Rafa Hortala‐Vallve and Matteo Galizzi). On top of experiments and core survey modules administered in each participating country, the Italian project includes a survey module on personality traits and a survey module on fertility intentions.

4 While the strategy method is supposed to reduce the degree to which trustees feel responsible towards their matched trustors, Johnson and Mislin (2011) find no significant effect either in terms of trust or of trustworthiness.

5 Burks et al. (2003) show that prior knowledge of playing in both TG roles leads players both to send and return lower amounts (lower trust and trustworthiness), most likely because of “reduced responsibility”: that is, the two‐role players perceive their responsibility in determining the counterpart's payoff as halved. In a meta‐analysis of more than 130 trust games, Johnson and Mislin (2011) find that playing both roles reduces trustworthiness significantly. If the same mechanism is at work in our study, the figures we find could be considered the lower bounds to “real” trust and trustworthiness. Moreover, there are no ex ante arguments predicting that “reduced responsibility” should differ between Southerners and Northerners; even if we expect that Southerners in two‐role TGs perceive less responsibility than Northerners, we should have found a significant North‐South gap both in trust and trustworthiness. Our results show that this is not the case.

6 Using a similar distinction, and relying on Banfield's conjectures about the cultural roots of backwardness in the South of Italy, Tabellini (2008) and Platteau (1994) argue that, by stimulating economic transactions outside the small circles of known persons (e.g., relatives, friends, etc.), generalized as opposed to limited morality is more conducive to economic growth.

7 The generalized trust question *GTQ* asks respondents “On a scale from zero to ten, where zero is not at all and 10 is completely, in general, how much do you trust most people?”

8 Respectively, the two questions ask “How often do you participate in voluntary activities to help people other than your direct relatives, friends or colleagues?”, and “How often do you get together with friends?”. Answers range from “Never” to “Daily.”

9 The respondent is asked how much she is willing to contribute to the common project, would the other three group members, j, have contributed on average by €0 up to €10. The average conditional contribution, ${\bar{c}}_{i}$, is computed as a simple average of the 11 conditional contributions by respondent i. The measure of reciprocity in the PGG is an average of eleven deviations of conditional contributions from the average conditional contribution, $c_{i,j}-{\bar{c}}_{i}$, with weights ranging from ‐5 to +5 attached, respectively, to the first up to the last deviation, dividing all by 110 (the maximum sum of individual contributions across all eleven cases). The first (last) deviation is the difference between the conditional contribution in the case of a €0 (€10) average contribution by other group members and the average conditional contribution. The resulting variable varies between −1.36 (inverse reciprocity) and +1.36 (direct reciprocity). The exact formula is:

$$Cond.cooperation_{i}=\frac{\sum_{j=0}^{10}\left(c_{i,j}-{\bar{c}}_{i}\right)\left(\;-\;5+j\right)}{10\;\cdot \;N_{j}}$$

where

$${\bar{c}}_{i}=\frac{\sum_{j=0}^{10}c_{i,j}}{N_{j}},\;N_{j}=11,\;c_{i,j}=\{0,\text{…},10\},\;i=\{1,\text{…},N\}$$

10 Risk can be deemed a dimension of prosocial behavior (and more in general of social capital) since it is often associated, theoretically and empirically, with trust (Albanese et al., 2017; Ashraf et al., 2006; Bohnet & Zeckhauser, 2004; Eckel & Wilson, 2004; Fehr, 2009; Karlan, 2005).

11 The lotteries in the risk ladder proposed to the respondent give the following payoffs in the case of success (s) and failure (f), both occurring with probability 0.5, listed by increasing risk: first lottery 8 (f)–8 (s), second lottery 7 (f)–10 (s), third lottery 6 (f)–12 (s), fourth lottery 5 (f)–14 (s), fifth lottery 4 (f)–16 (s), sixth lottery 1 (f)–19 (s).

12 According to Mann–Whitney *U* tests the South and the rest of Italy rank in a not significantly different way in terms of self‐reported trust (*Z* statistic equal to 0.955), experimental trust (0.843), expected trustworthiness (0.877), while experimental trustworthiness is significantly lower in the South (2.587***).

13 The South and the rest of Italy rank similarly in terms of cooperation (*Z* statistic from the Mann–Whitney *U* test equal to 0.365), conditional cooperation (1.239), altruism trustworthiness (0.539), and risk propensity (0.547).

14
*Z* statistics from Mann–Whitney *U* tests are equal to 2.323** as for voluntary work, and to 0.094 as for social engagement.

15 This leads to the exclusion of 33 respondents from our sample. Results are not dramatically affected if we include them in the analysis.

16 All results presented in the paper are robust to using Tobit regressions (available upon request).

17 Control variables are expressed as categories in the case of gender (dummy indicating female respondents), age (five groups, namely 18–24—reference, 25–34, 35–44, 45–54, 55–64), education (three groups, namely high school or less—reference, some college or nontertiary diploma, tertiary education), job status (four groups, namely employed—reference, self‐employed, unemployed, inactive), marital status (three groups, namely single—reference, married, divorced/separated/widowed), residential area (five groups, namely rural, village—reference, urban, small or middle area, large metropolitan area), parental education (two dummies indicating the parent completed at least secondary education, both for the father and the mother), whereas they are kept continuous in the case of household size (number of people in the household, ranging from 1 to 8), personal income (expressed in logarithm). Each personality trait variable results from averaging the answers to the three items capturing the respective personality dimension, after the appropriate recoding. Other experimental measures are added as continuous variables as specified in Table 3.

18 The value of *R* ^2^ increases when personality traits are introduced in the regressions, yet this happens to a larger degree when survey trust is set as outcome. In general, in the Trustlab sample personality traits correlate higher with variables measured through survey questions than through behavioral experiments. This might be due to the fact that individuals' answers in surveys might be biased by factors related to their personality. Whereas the resulting noise in survey outcomes might be “cleaned” by controlling for personality traits, it is likely that experimental outcomes suffer less from this form of bias because of the very design of experiments.

19 Following Heckman's two‐step selection model, we adjust our estimates with the probability of participating in Trustlab. This probability is estimated controlling for frequency of internet access and, in an additional model, for the quality of the broadband internet infrastructure. See the Appendix B for further details.

20 Raw differences shown in Tables A1–A3 in Appendix A provide similar results to those presented in Tables 7, 9, and 10. In addition, unreported analyses where experimental conditions are controlled for provide again similar results (available upon request).

21 The data provide support for this hypothesis since the subject's beliefs about the opponent's trustworthiness and his or her own level of trustworthiness are highly correlated (ρ=0.50). A positive correlation between trust and trustworthiness is also found in previous studies in which, as in our experiment, subjects play both roles in the TG (Altmann et al., 2008; Chaudhuri & Gangadharan, 2007; Glaeser et al., 2000; Kovacs & Willinger, 2013).

22 In further specifications of Tables 11 and 12 we included among controls generalized trust measured through the survey question in addition to experimental measures. The correlation between survey trust and experimental trust and with experimental trustworthiness is positive and significant only when personality traits are not accounted for. The coefficient on the South dummy remains similar in terms of magnitude and significance in both cases.

23 Collinearity problems do not arise in any model estimated in the paper. The Variance Inflation Factor (VIF) is always far from the critical threshold of 10: it gets larger than 2 on age and job status dummies, but never exceeds 4.5.

24 We check for geographical patterns in overall prosociality measured through the complex of social preferences elicited and assessed in Trustlab. Specifically, we generate an index of prosociality by extracting the first principal component of the five variables selected according to the analysis reported in Table A4 in Appendix A (we exclude variables showing low item‐test correlations and leaving high Cronbach's α in the latent index once removed). The index has a reliability score of 0.686, an eigenvalue of 2.94 and retains 48% of total variation. In Table A5 in Appendix A the index is regressed on all macroareas or on the South macroarea only, controlling for socio‐demographics and personality traits. Southern Italy is less inclined to prosocial behavior, yet the correlation is never significant at standard levels. These results further corroborate our main argument since, overall, Southerners do not appear highly dissimilar from other Italians in terms of prosociality.

25 Furthermore, the percentage of trustors sending €5, €9, or €10 is statistically indistinguishable between the South and the rest of Italy (z=0.216;p=0.8292).

26 Available upon request.

27 While we decided to divide the Italian territory into five macroareas according to the official NUTS adopted by Eurostat (i.e., Italian macroareas are NUTS‐1 areas corresponding to major socioeconomic regions of the European Union), the main results of the paper are robust to alternative classifications of macroareas. In Appendix A we replicate specifications from Tables 9, 11, 12, and 13 first by including the Islands into the Southern macroarea, and second by defining a unique Northern macroarea and excluding the Center. Tables A8‐A10 report results according to the former alternative classification (i.e., Sicily and Sardinia being part of the peninsular South), whereas results in Tables A11‐A13 stem from the latter alternative classification (i.e., merging North‐western and North‐eastern Italy, and excluding the Center from the sample).

28 Personality traits were not measured again in the follow‐up, so we work under the assumption that personality has remained constant within respondent between October 2017 and May 2018. This is not a very strong assumption in our sample, as vast literature documents that personality traits change more during teenage years whereas they tend to remain more stable at later ages (Caspi et al., 2005; Donnellan & Lucas, 2008).

29 The full sample of Trustlab Italy oversamples female respondents between 18 and 45 years of age to study specific issues related to fertility behavior and family demography. These additional respondents have been excluded from all the estimates carried out to fully exploit the national representativeness of the data.

30 To measure aversion to betrayal, Bigoni et al. (2019) implement the experimental procedure developed by Bohnet et al. (2008) on a sample of students at the University of Bologna, who were recruited so as to cover the South and the North of Italy (excluding the Center). They exploit a between‐subjects design where individuals take part in a trust game and risky dictator game aimed at disentangling the behavioral response to risk originating from social interaction as opposed to nature. The authors find larger minimum acceptable probabilities (MAPs of finding reciprocating trustees) among participants in the trust game compared to participants in the risky dictator game. The larger the MAP, the more the trustor is considered as averse to risk taking. These results are interpreted as evidence of overall betrayal aversion, which appears to be systematically high and statistically significant only among trustors from the South. We could not measure betrayal aversion experimentally in Trustlab because of the high number of experimental tasks and survey questions to which respondents were already exposed. Moreover, the calculation of MAPs might have been a difficult task for a population composed not only of students, and therefore the Bohnet et al. (2008)'s experimental procedure would have likely produced noisy and unreliable data.

31 The vignette‐based approach addresses the potential shortcomings of the experimental tasks in Bohnet et al. (2008) that stem from the complex incentive structure. However, Cubitt et al. (2017) find significantly higher proportions of people choosing either higher MAPs (in the experimental setting) or safe options (in the vignettes) when facing social risk. Hence, both approaches consistently measure similar patterns of betrayal aversion.

32 Unlike Cubitt et al. (2017) who use a between‐subject design, we carry out a within‐subjects design, that is, each respondent chooses between the risky and the safe company in *both* the social‐ and the natural‐risk vignettes. However, the order of vignettes is randomized, and the names of companies differ across vignettes to mitigate potential order effects and response biases.

33 Differently from our vignette‐based approach run at the individual level, Bigoni et al. (2019) rely on an incentivized design in which betrayal aversion is assessed based on a comparison of choices between subjects in a trust as opposed to a risky dictator game.

34 In an additional specification we also control for the order in which the two scenarios are presented. Standard errors are clustered at the individual level to adjust for the doubling of sample size induced by our within‐subject design.

35 The distribution of types does not significantly correlate with trust either, yet the sign of the correlation is as expected: risk lovers and principled trustful individuals show higher trust, whereas lower trust can be found among risk averse and (even lower) betrayal averse individuals.

36 A further qualification of this theory is provided by Ermisch and Gambetta (2010), who posit that in societies where family ties are less stringent, people feel more motivated to deal with unknown persons to fulfill socioeconomic transactions and to face more opportunities (not ultimately because interacting more with strangers increases learning), thereby fostering higher trust in strangers. Furthermore, Enke (2019) provides cross‐country evidence of a positive relationship between ancestral kinship tightness and ingroup bias in present levels of trust and cooperation.

37 The geographical distribution of family ties in our sample is consistent with that obtained using the same proxy for family ties built through EVS data for Italy (available upon request).

38 A *χ* ^2^ test rejects at the 1% level the hypothesis that the distribution of family ties is equal across macroareas ($\chi^{2}\left(52\right)=78.4,\;p=0.01$). Nonparametric tests confirm that the family ties are significantly stronger in the South than in the North ($z‐score=‐3.87,\;p\leq 0.001;\;\chi^{2}\left(13\right)=33.7,\;p=0.001$).

39 In addition, the equality of coefficients between the group of current residents in the South and that of migrants from South to North before childhood (*F* = 1.90) and after childhood (*F* = 1.50) cannot be rejected based on estimates from column 3 of Table A23.
