# Supplementary material for: Was Banfield right? New insights from a nationwide laboratory experiment
Source: J Reg Sci. 2021 Jun 15;61(5):1029–64. doi: 10.1111/jors.12538 (PMC8596913; doi:10.1111/jors.12538)
Supplement: Supplementary file 1 — Supplementary Information [file JORS-61-1029-s001.pdf]

Appendix A

The classification of Italian macroareas used in the paper is the official classification adopted by national and supranational statistical offices like Istat and Eurostat, also known as Nomenclature of Territorial Units for Statistics (NUTS). The NUTS classification has been in use at the European level since the early 1970s and was formally recognised starting from 2003. In light of the subject and the purpose of the paper, i.e. to document whether there exist geographical divides in social preferences, it is important to compare territorial units characterized by internal similarity in regards to social, cultural and economic aspects. This is best achieved by using the five Italian macroareas (*ripartizioni geografiche*), which are NUTS-1 areas corresponding to major socio-economic regions in the European Union (https://ec.europa.eu/eurostat/web/nuts/background).

According to the NUTS classification, the two major Italian Islands (Sicily and Sardinia) form a separate macroarea which accounts for 11% of the Italian population and for 16.5% of the Italian territory. One could use a more inclusive definition of the South by including the Islands in the Southern macroarea, e.g. according to the cultural and institutional roots in the Bourbons kingdom (*Regno delle due Sicilie*). Yet one could go even further back in the Italian history and assess the legacy of differential patterns of local public governance well before the medieval age. Or one might even exploit within-macroareas variation by looking, for example, at the persistent effects of different governance strategies adopted by maritime and communal republics in the same macroarea (see the recent work by Buonanno, Cervellati, Lazzaroni & Prarolo, 2019).

There are, indeed, historical factors that make Italian Islands a specific case. First, Sicily and Sardinia are the oldest "special" regions. According to Putnam, Leonardi, and Nanetti (1993), the attempt of fostering regional identities remained silent until 1948 since it was perceived as incompatible with the post-unification priority of national development, under the slogan "making the Italians". The new Constitution of 1948 allowed for directly elected regional governments, which was carried out immediately in five "special" regions located along the national borders and on the Islands of Sicily and Sardinia, which were already areas threatened by separatism and ethnic problems.

Second, a recent estimation of real wage dynamics from the Italian unification to 1913 for five main macro-regional areas of Italy (i.e. the same categories used in our paper) highlights that the regional divide was already large at unification (Federico, Nuvolari & Vasta, 2019); this evidence is also consistent with Felice (2014), who estimated that the gap between the North-Centre and the South of the country was already 18% in 1871, and hence probably also in 1861. Following the estimation by Federico et al. (2019), wages were about 15% higher in the North than in the South, including Islands, and 20% higher excluding Islands. This last comparison is important for our purpose. More specifically, the authors find a relatively high level of real wages in the Islands relative to wages in the continental South. They explained that by describing the peculiar settlement of the agricultural workforce in Sicily, i.e. the so called "agro-towns", large agglomeration where the workforce clustered since the Middle Ages. This type of settlement prevented women from seeking agricultural employment, generating low female employment and higher wages for men to guarantee the survival of the household. Interestingly, the authors also identify a sharp rise of wages in the 1870s in the Islands, which they attribute to a massive wave of public works, especially in railways. Other estimates suggest that from 1861 to 1871 there was not yet an industrialization gap between the North and South of Italy (Daniele & Malanima, 2014); again Sicily emerges as an outlier in the South, ranking highest in terms of industrial workers compared to other Italian regions, with shares similar to those estimated for Tuscany and Lombardy (Bevilacqua, 2005; Fenoaltea, 2001; Fenoaltea, 2006; Ciccarelli & Fenoaltea, 2012). These historical peculiarities of Italian islands might have played a role in shaping social relationships and cooperation dynamics, which could put these regions on a potentially different path of social capital accumulation.

Third, quoting the Times Atlas of World History, Putnam et al. (1993) highlights that by the end of the 12th century, Sicily, by controlling the Mediterranean Sea routes, was the richest, most advanced, and highly organized State in Europe. Moreover, the author's estimates of civic capital across Italian regions show that Sicily and Sardinia share a very similar pattern of civic traditions, yet with lower levels than in Campania and Calabria and with higher levels than Molise and Basilicata (which rank lowest).

Finally, recent historical estimates of living standards in Italy show, again using the

same macroarea division as in our paper, that human capital levels were different in Sicily and Sardinia from those measured for the other Southern regions (Vecchi, 2017). More specifically, literacy rates in the south in 1861 were almost the same (about 13.8% in all southern regions), while in Sicily and Sardinia they were similarly lower (11.3% and 11.00% respectively); the same results emerge when comparing macroareas in terms of height (a proxy for health) in the 1880s.

All these historical facts suggest that, while there are cultural similarities between Sicily and the other Southern Italian regions, there are also reasons for treating Sicily (and also Sardinia) as institutionally and economically different entities from the continental South, thereby motivating our macroarea differentiation followed also by other historical studies (Federico et al., 2019; Felice & Vasta, 2015; Daniele, 2015).

At the same time, there are historical reasons for considering Sicily and Sardinia as being part of a broad South macroarea. So, in the attempt of balancing the aforementioned motivations for keeping the Islands outside or inside the South category, and reducing the inevitable degree of arbitrariness in picking the part of history that better fits hypotheses, we have decided to i) keep our original classification of macroareas, which is the official division adopted by Istat; and ii) run a robustness check on our preferred model to test the stability of our results when including Islands into the South macroarea.

We consider first all the behavioral outcomes but trust and average trustworthiness (Table A8), then we concentrate on the latter (Table A9). We also repeat the analysis of trustworthiness conditional on the trustor's transfer (Table A10). Results are qualitative similar to those obtained in the original version of the paper (i.e. a gap in social capital emerges only for trustworthiness), yet they are weaker in terms of statistical significance. This might be due to the fact that trustworthiness is high in the Islands, and hence adding them to the South category reduces the estimated gap in trustworthiness. Even if one interprets this as a "null result", the lack of dramatic differences in trustworthiness across Italian macroareas is still consistent with the general message of our paper: there might not necessary be North-South differences in terms of generalized other-regarding preferences.

To further address this point and highlight potential differences between this alternative classification of the North and the South, we now drop from the analysis the regions from the Center, and compare just the South (including Islands) with the North (including both North West and North East). As above, we take our preferred specification and consider first all the behavioral outcomes but trust and average trustworthiness (Table A11), then we concentrate on the latter (Table A12). We also repeat the analysis of trustworthiness conditional on the trustor's transfer (Table A13). Results are broadly consistent with those obtained by using our original macroarea classification, with a gap in social capital emerging only for trustworthiness and conditional reciprocity (though only when the latter is modeled linearly). Differently from the results showed previously, here the gap in trustworthiness is highly statistically significant, further suggesting that the two macroareas broadly considered do differ in this specific dimension of social capital.

References

Bevilacqua, P. (2005). *Breve storia dell'Italia meridionale: Dall'Ottocento a oggi*. Donzelli editore.

Buonanno, P., Cervellati, M., Lazzaroni, S., & Prarolo, G. (2019). Political history, fiscal compliance and cooperation: Medieval social contracts and their legacy.

Ciccarelli, C., & Fenoaltea, S. (2012). The rail-guided vehicles industry in Italy, 1861–1913: The burden of the evidence. *Research in Economic History*, *28*, 43–115.

Daniele, V. (2015). Two Italies? genes, intelligence and the italian north–south economic divide. *Intelligence*, *49*, 44–56.

Daniele, V., & Malanima, P. (2014). Falling disparities and persisting dualism: Regional development and industrialisation in Italy, 1891–2001. *Investigaciones de Historia Economica - Economic History Research*, *10*(3), 165–176.

Federico, G., Nuvolari, A., & Vasta, M. (2019). The origins of the italian regional divide: Evidence from real wages, 1861–1913. *The Journal of Economic History*, *79*(1), 63–98.

Felice, E. (2014). Il mezzogiorno fra storia e pubblicistica. Una replica a Daniele e Malanima. *Rivista di storia economica*, (2/2014), 197–242.

Felice, E., & Vasta, M. (2015). Passive modernization? The new human development index and its components in Italy's regions (1871–2007). *European Review of Economic History*, *19*(1), 44–66.

Fenoaltea, S. (2001). *La crescita industriale delle regioni d'Italia dall'unità alla grande guerra: Una prima stima per gli anni censuari*. Banca d'Italia.

Fenoaltea, S. (2006). *L'economia italiana dall'unità alla grande guerra*. Laterza.

Putnam, R. D., Leonardi, R., & Nanetti, R. Y. (1993). *Making democracy work: Civic traditions in modern Italy*. Princeton, NJ: Princeton university press.

Vecchi, G. (2017). *Measuring wellbeing: A history of italian living standards*. Oxford University Press.

TABLE A1: Generalized trust, experimental trust, trustworthiness and expected trustworthiness (South vs. rest of Italy) - Raw differences

	GTQ	Trust	Trustworthiness	Expected trustworthiness
	(1)	(2)	(3)	(4)
South	-0.217	-0.125	-0.767**	0.0319
	(0.171)	(0.224)	(0.372)	(0.426)
Observations	1,011	1,016	1,016	1,016
R-squared	0.002	0.000	0.004	0.000

Notes: Robust standard errors in parentheses. *** p<0.01, ** p<0.05, * p<0.1.

TABLE A2: Altruism, cooperation, conditional cooperation and risk propensity (South vs. rest of Italy) - Raw differences

	Altruism	Cooperation	Conditional cooperation	Risk propensity
	(1)	(2)	(3)	(4)
South	-0.0900	-0.153	0.0186	0.145
	(0.169)	(0.225)	(0.0323)	(0.125)
Observations	1,016	1,016	1,016	1,016
R-squared	0.000	0.000	0.000	0.001

Notes: Robust standard errors in parentheses. *** p<0.01, ** p<0.05, * p<0.1.

TABLE A3: Differences in social engagement (South vs. rest of Italy) - Raw differences

	Voluntary work	Encounters with friends
	(1)	(2)
South	0.177	0.480***
	(0.138)	(0.143)
Observations	1,016	1,016
R-squared	0.000677	0.00438

Notes: Robust standard errors in parentheses. *** p<0.01, ** p<0.05, * p<0.1.

TABLE A4: Analysis of items for an index of prosociality

	Item-Test correlation	Cronbach's α after removal
GTQ	0.303	0.622
Trust	0.608	0.562
Trustworthiness	0.763	0.529
Exp. Trustworthiness	0.761	0.546
Altruism	0.609	0.565
Cooperation	0.546	0.579
Conditional cooperation	0.066	0.631
Risk propensity	0.212	0.628
Volunteering	0.152	0.628
Encounters with friends	0.147	0.629

TABLE A5: Index of pro-sociality by macroarea

	Index of pro-sociality			
	(1)	(2)	(3)	(4)
North-east	-0.252*	-0.267*		
	(0.144)	(0.146)		
Center	-0.203	-0.209		
	(0.138)	(0.142)		
South	-0.160	-0.178	-0.0977	-0.117
	(0.139)	(0.141)	(0.119)	(0.121)
Islands	0.314	0.352*		
	(0.212)	(0.213)		
Controls	Yes	Yes	Yes	Yes
PTs	No	Yes	No	Yes
Observations	1,015	979	1,015	979
R-squared	0.038	0.047	0.028	0.035

Notes: Robust standard errors in parentheses. *** p<0.01, ** p<0.05, * p<0.1.

TABLE A6: Determinants of reciprocal strategies (trust game)

	Selfish		Break even		Reciprocal	
	(1)	(2)	(3)	(4)	(5)	(6)
South	0.0228	0.00344	0.0330	0.00896	-0.0674**	-0.00818
	(0.0162)	(0.0215)	(0.0229)	(0.0255)	(0.0305)	(0.0358)
Send	0.0131***	0.0124***	-0.00299***	-0.00396***	-0.0125***	-0.0102***
	(0.00128)	(0.00135)	(0.00105)	(0.00114)	(0.00158)	(0.00170)
Send * South		0.00291		0.00416		-0.0100**
		(0.00248)		(0.00278)		(0.00398)
Trust	-0.0109***	-0.0109***	-0.00889**	-0.00890**	0.0226***	0.0226***
	(0.00264)	(0.00264)	(0.00387)	(0.00387)	(0.00505)	(0.00506)
Expected trustworthiness	-0.00428***	-0.00429***	-0.00516**	-0.00517**	0.0104***	0.0105***
	(0.00157)	(0.00157)	(0.00217)	(0.00217)	(0.00294)	(0.00294)
Altruism	-0.00602*	-0.00601*	0.000290	0.000313	0.00631	0.00628
	(0.00361)	(0.00361)	(0.00559)	(0.00559)	(0.00731)	(0.00731)
Cooperation	-0.00962***	-0.00963***	-0.00238	-0.00237	0.0136***	0.0136***
	(0.00279)	(0.00279)	(0.00375)	(0.00375)	(0.00504)	(0.00505)
Risk propensity	0.00913**	0.00914**	-0.00981	-0.00981	0.000089	0.000075
	(0.00412)	(0.00413)	(0.00618)	(0.00618)	(0.00819)	(0.00819)
Controls	Yes	Yes	Yes	Yes	Yes	Yes
PTs	Yes	Yes	Yes	Yes	Yes	Yes
Observations	9,790	9,790	9,790	9,790	9,790	9,790

Notes: Zero transfers are excluded from the sample. Robust standard errors in parentheses, clustered at the individual level. *** p<0.01, ** p<0.05, * p<0.1.

TABLE A7: The North-South divide in conditional cooperation (public goods game)

	Conditional cooperation			
	(1)	(2)	(3)	(4)
South	-0.0330	-0.0763	-0.0330	-0.202
	(0.135)	(0.214)	(0.135)	(0.221)
Send	0.633***	0.631***	0.700***	0.680***
	(0.0134)	(0.0149)	(0.0219)	(0.0248)
Send * South		0.00866		0.0922*
		(0.0333)		(0.0531)
Send squared			-0.00669***	-0.00489**
			(0.00167)	(0.00190)
Send squared * South				-0.00835**
				(0.00400)
Trust	0.0876***	0.0876***	0.0876***	0.0876***
	(0.0229)	(0.0229)	(0.0229)	(0.0229)
Trustworthiness	0.107***	0.107***	0.107***	0.107***
	(0.0157)	(0.0157)	(0.0157)	(0.0157)
Expected trustworthiness	-0.0241*	-0.0241*	-0.0241*	-0.0241*
	(0.0140)	(0.0140)	(0.0140)	(0.0140)
Altruism	0.0987***	0.0987***	0.0987***	0.0987***
	(0.0316)	(0.0316)	(0.0316)	(0.0316)
Risk propensity	0.0612	0.0612	0.0612	0.0612
	(0.0379)	(0.0379)	(0.0379)	(0.0379)
Controls	Yes	Yes	Yes	Yes
PTs	Yes	Yes	Yes	Yes
Observations	10,769	10,769	10,769	10,769
R-squared	0.478	0.478	0.479	0.479

Notes: Robust standard errors in parentheses, clustered at the individual level. *** p<0.01, ** p<0.05, * p<0.1.

TABLE A8: Altruism, cooperation, conditional cooperation and risk propensity by macroarea (alternative classification of macroareas - South)

	Altruism	Cooperation	Cond. cooperation	Risk propensity
	(1)	(2)	(3)	(4)
South and Islands	0.245	0.0593	-0.00977	0.146
	(0.171)	(0.208)	(0.0297)	(0.115)
Controls	Yes	Yes	Yes	Yes
PTs	Yes	Yes	Yes	Yes
Observations	979	979	979	979
R-squared	0.033	0.055	0.075	0.034

Notes: Robust standard errors in parentheses. *** p<0.01, ** p<0.05, * p<0.1.

TABLE A9: The rationales of trust and trustworthiness (alternative classification of macroareas - South)

	Trust		Trustworthiness	
	(1)	(2)	(3)	(4)
South and Islands	0.157	0.144	-0.558*	-0.569*
	(0.178)	(0.189)	(0.304)	(0.305)
Trustworthiness	0.103***	0.127***		
	(0.0207)	(0.0214)		
Trust			0.299***	0.335***
			(0.0604)	(0.0561)
Expected trustworthiness	0.0321	0.0358*	0.390***	0.395***
	(0.0209)	(0.0213)	(0.0399)	(0.0401)
Altruism	0.226***	0.334***	0.297***	0.334***
	(0.0461)	(0.0453)	(0.0861)	(0.088)
Cooperation	0.306***		0.115**	
	(0.0349)		(0.0561)	
Cond. cooperation		-0.0829		0.714*
		(0.219)		(0.371)
Risk propensity	0.00159	0.037	-0.0965	-0.0692
	(0.0543)	(0.0568)	(0.0925)	(0.0932)
Controls	Yes	Yes	Yes	Yes
PTs	Yes	Yes	Yes	Yes
Observations	979	979	979	979
R-squared	0.305	0.233	0.351	0.351

Notes: Robust standard errors in parentheses. *** p<0.01, ** p<0.05, * p<0.1.

TABLE A10: The North-South divide in conditional reciprocity (alternative classification of macroareas - South)

	Reciprocity			
	(1)	(2)	(3)	(4)
South and Islands	-0.558*	-0.00240	-0.558*	-0.140
	(0.299)	(0.250)	(0.299)	(0.249)
Send	1.475***	1.511***	1.450***	1.456***
	(0.0274)	(0.0316)	(0.0344)	(0.0413)
Send * South and Islands		-0.111*		-0.0196
		(0.0615)		(0.0746)
Send squared			0.00254	0.00547*
			(0.00254)	(0.00314)
Send squared * South and Islands				-0.00916*
				(0.00530)
Trust	0.299***	0.299***	0.299***	0.299***
	(0.0596)	(0.0596)	(0.0596)	(0.0596)
Expected trustworthiness	0.390***	0.390***	0.390***	0.390***
	(0.0393)	(0.0393)	(0.0393)	(0.0393)
Cooperation	0.115**	0.115**	0.115**	0.115**
	(0.0553)	(0.0553)	(0.0553)	(0.0553)
Altruism	0.297***	0.297***	0.297***	0.297***
	(0.0849)	(0.0849)	(0.0849)	(0.0849)
Risk propensity	-0.0965	-0.0965	-0.0965	-0.0965
	(0.0911)	(0.0911)	(0.0911)	(0.0911)
Controls	Yes	Yes	Yes	Yes
PTs	Yes	Yes	Yes	Yes
Observations	10,769	10,769	10,769	10,769
R-squared	0.542	0.543	0.542	0.543

Notes: Robust standard errors in parentheses, clustered at individual level. *** p<0.01, ** p<0.05, * p<0.1.

TABLE A11: Altruism, cooperation, conditional cooperation and risk propensity by macroarea (alternative classification of macroareas - North)

	Altruism	Cooperation	Cond. cooperation	Risk propensity
	(1)	(2)	(3)	(4)
South and Islands	0.211	0.165	-0.00582	0.107
	(0.182)	(0.223)	(0.0315)	(0.125)
Controls	Yes	Yes	Yes	Yes
PTs	Yes	Yes	Yes	Yes
Observations	777	777	777	777
R-squared	0.055	0.059	0.084	0.043

Notes: Robust standard errors in parentheses. The reference macroarea is North (including North-west and North-east). Respondents from the Central macroarea are excluded from the sample. *** p<0.01, ** p<0.05, * p<0.1.

TABLE A12: The rationales of trust and trustworthiness (alternative classification of macroareas - North)

	Trust		Trustworthiness	
	(1)	(2)	(3)	(4)
South and Islands	0.169	0.199	-0.753**	-0.751**
	(0.190)	(0.201)	(0.322)	(0.323)
Trustworthiness	0.0957***	0.121***		
	(0.0234)	(0.0243)		
Trust			0.273***	0.315***
			(0.0675)	(0.0638)
Expected trustworthiness	0.0301	0.0339	0.396***	0.401***
	(0.0236)	(0.0239)	(0.0436)	(0.0440)
Altruism	0.239***	0.361***	0.345***	0.390***
	(0.0526)	(0.0508)	(0.0988)	(0.101)
Cooperation	0.306***		0.142**	
	(0.0398)		(0.0646)	
Cond. cooperation		0.153		0.522
		(0.246)		(0.415)
Risk propensity	0.0134	0.0414	-0.0945	-0.0727
	(0.0614)	(0.0641)	(0.101)	(0.102)
Controls	Yes	Yes	Yes	Yes
PTs	Yes	Yes	Yes	Yes
Observations	777	777	777	777
R-squared	0.322	0.254	0.382	0.380

Notes: Robust standard errors in parentheses. The reference macroarea is North (including North-west and North-east). Respondents from the Central macroarea are excluded from the sample. *** p<0.01, ** p<0.05, * p<0.1.

TABLE A13: The North-South divide in conditional reciprocity (alternative classification of macroareas - North)

	Reciprocity			
	(1)	(2)	(3)	(4)
South and Islands	-0.753**	-0.0498	-0.753**	-0.155
	(0.316)	(0.271)	(0.317)	(0.272)
Send	1.484***	1.540***	1.479***	1.507***
	(0.0312)	(0.0380)	(0.0384)	(0.0488)
Send * South and Islands		-0.141**		-0.0706
		(0.0651)		(0.0790)
Send squared			0.000499	0.00332
			(0.00280)	(0.00370)
Send squared * South and Islands				-0.00701
				(0.00565)
Trust	0.273***	0.273***	0.273***	0.273***
	(0.0662)	(0.0662)	(0.0662)	(0.0663)
Expected trustworthiness	0.396***	0.396***	0.396***	0.396***
	(0.0428)	(0.0428)	(0.0428)	(0.0428)
Cooperation	0.142**	0.142**	0.142**	0.142**
	(0.0634)	(0.0634)	(0.0634)	(0.0634)
Altruism	0.345***	0.345***	0.345***	0.345***
	(0.0970)	(0.0970)	(0.0970)	(0.0970)
Risk propensity	-0.0945	-0.0945	-0.0945	-0.0945
	(0.0992)	(0.0992)	(0.0992)	(0.0992)
Controls	Yes	Yes	Yes	Yes
PTs	Yes	Yes	Yes	Yes
Observations	8,547	8,547	8,547	8,547
R-squared	0.554	0.555	0.554	0.555

Notes: Robust standard errors in parentheses, clustered at individual level. The reference macroarea is North (including North-west and North-east). Respondents from the Central macroarea are excluded from the sample. *** p<0.01, ** p<0.05, * p<0.1.

TABLE A14: The North-South gap in trustworthiness: the role of social capital

	Trustworthiness			
	(1)	(2)	(3)	(4)
South	-0.879***	-0.856***	-0.838**	-0.855**
	(0.329)	(0.327)	(0.333)	(0.335)
Voted in last election	-0.106			-0.114
	(0.475)			(0.488)
Never volunteers		-0.161		-0.292
		(0.280)		(0.299)
Connectedness with neighbours			-0.0562	-0.0606
			(0.0593)	(0.0625)
Trust	0.284***	0.297***	0.294***	0.281***
	(0.0609)	(0.0603)	(0.0613)	(0.0619)
Expected trustworthiness	0.398***	0.387***	0.392***	0.400***
	(0.0402)	(0.0396)	(0.0401)	(0.0407)
Altruism	0.292***	0.293***	0.305***	0.300***
	(0.0875)	(0.0856)	(0.0865)	(0.0883)
Cooperation	0.100*	0.115**	0.113**	0.101*
	(0.0563)	(0.0560)	(0.0568)	(0.0571)
Risk propensity	-0.108	-0.0911	-0.0900	-0.104
	(0.0934)	(0.0920)	(0.0932)	(0.0946)
Controls	Yes	Yes	Yes	Yes
PTs	Yes	Yes	Yes	Yes
Observations	956	979	965	943
R-squared	0.355	0.353	0.353	0.356

Notes: Robust standard errors in parentheses. *** p<0.01, ** p<0.05, * p<0.1.

TABLE A15: Original sample vs. extended sample with population weights

	Trustworthiness			
	(1)	(2)	(3)	(4)
South	-0.854***	-0.861***	-0.980***	-0.986***
	(0.327)	(0.328)	(0.306)	(0.305)
Trust	0.297***	0.333***	0.308***	0.349***
	(0.0603)	(0.0559)	(0.0603)	(0.0555)
Expected trustworthiness	0.388***	0.393***	0.359***	0.363***
	(0.0396)	(0.0398)	(0.0372)	(0.0374)
Altruism	0.294***	0.331***	0.289***	0.333***
	(0.0856)	(0.0875)	(0.0837)	(0.0861)
Cooperation	0.114**		0.122**	
	(0.0560)		(0.0521)	
Cond. cooperation		0.706*		0.841**
		(0.371)		(0.351)
Risk propensity	-0.0928	-0.0658	-0.114	-0.0854
	(0.0920)	(0.0929)	(0.0877)	(0.0877)
Extended sample			-0.146	-0.0903
			(0.351)	(0.353)
Controls	Yes	Yes	Yes	Yes
PTs	Yes	Yes	Yes	Yes
Observations	979	979	1,406	1,406
R-squared	0.353	0.353	0.328	0.329

Notes: Robust standard errors in parentheses. Columns 3 and 4 include population weights adjusting the sample composition to the gender and age-group structure of Italian population as of 2017 by macroarea of residence. *** p<0.01, ** p<0.05, * p<0.1.

TABLE A16: Original sample vs. extended sample with population weights

	Reciprocity			
	(1)	(2)	(3)	(4)
South	-0.854***	-0.0579	-0.980***	-0.114
	(0.322)	(0.275)	(0.302)	(0.248)
Send	1.475***	1.510***	1.439***	1.480***
	(0.0274)	(0.0302)	(0.0264)	(0.0296)
Send * South		-0.159**		-0.173***
		(0.0702)		(0.0646)
Trust	0.297***	0.297***	0.308***	0.308***
	(0.0594)	(0.0594)	(0.0597)	(0.0597)
Expected trustworthiness	0.388***	0.388***	0.359***	0.359***
	(0.0390)	(0.0390)	(0.0368)	(0.0368)
Altruism	0.294***	0.294***	0.289***	0.289***
	(0.0844)	(0.0844)	(0.0828)	(0.0828)
Cooperation	0.114**	0.114**	0.122**	0.122**
	(0.0551)	(0.0552)	(0.0515)	(0.0515)
Risk propensity	-0.0928	-0.0928	-0.114	-0.114
	(0.0907)	(0.0907)	(0.0868)	(0.0868)
Extended sample			-0.146	-0.146
			(0.347)	(0.347)
Controls	Yes	Yes	Yes	Yes
PTs	Yes	Yes	Yes	Yes
Observations	10,769	10,769	15,466	15,466
R-squared	0.543	0.544	0.523	0.524

Notes: Robust standard errors in parentheses. Columns 3 and 4 include population weights adjusting the sample composition to the gender and age-group structure of Italian population as of 2017 by macroarea of residence. *** p<0.01, ** p<0.05, * p<0.1.

TABLE A17: Share of safe choices across vignettes

	Social risk		Natural risk		Difference	Z stat.	p-value
All macroareas	76.84	[42.20]	66.01	[47.39]	10.84	-5.52	0
North-West	75.59	[43.03]	67.89	[46.77]	7.69	-2.09	0.037
North-East	77.05	[42.17]	60.33	[49.06]	16.72	-3.45	0.001
Center	76.96	[42.21]	66.18	[47.43]	10.78	-2.41	0.016
South	73.28	[44.34]	64.78	[47.86]	8.50	-2.04	0.041
Islands	86.40	[34.42]	72.00	[45.08]	14.40	-2.80	0.005

Notes: p-values based on Mann-Whitney U tests.

TABLE A18: Betrayal aversion in Italy

	Safe choice							
	(1)	(2)	(3)	(4)	(5)	(6)	(7)	(8)
Social risk treatment	0.0930***	0.0930***	0.0937***	0.0979***	0.0930***	0.0930***	0.0936***	0.0979***
	(0.0170)	(0.0170)	(0.0191)	(0.0198)	(0.0170)	(0.0170)	(0.0191)	(0.0198)
South		-0.0435	-0.0423	-0.0400		-0.0417	-0.0404	-0.0371
		(0.0298)	(0.0379)	(0.0384)		(0.0297)	(0.0377)	(0.0382)
Social risk treatment * South			-0.00256	-0.0119			-0.00255	-0.0119
			(0.0410)	(0.0421)			(0.0410)	(0.0421)
Extended sample	-0.0567**	-0.0561**	-0.0561**	-0.0603*	-0.0568**	-0.0562**	-0.0562**	-0.0599*
	(0.0259)	(0.0259)	(0.0259)	(0.0342)	(0.0259)	(0.0259)	(0.0259)	(0.0342)
Order of vignettes					-0.0485*	-0.0472*	-0.0472*	-0.0540**
(natural risk first)					(0.0252)	(0.0252)	(0.0252)	(0.0245)
Controls	No	No	No	Yes	No	No	No	Yes
PTs	No	No	No	Yes	No	No	No	Yes
Observations	2,117	2,117	2,117	2,035	2,117	2,117	2,117	2,035
R-squared	0.013	0.015	0.015	0.071	0.016	0.018	0.018	0.075

Notes: Robust standard errors in parentheses clustered at individual level. All columns include population weights adjusting the sample composition to the gender and age-group structure of Italian population as of 2017 by macroarea of residence. *** p<0.01, ** p<0.05, * p<0.1.

TABLE A19: Trustworthiness by type of risk propensity, adjusting for other social preferences

	Trustworthiness				
	(1)	(2)	(3)	(4)	(5)
South	-0.796**	-0.788**	-0.797**	-0.796**	-0.789**
	(0.354)	(0.354)	(0.353)	(0.354)	(0.354)
Risk averse	0.00641				
	(0.312)				
Risk lover		-0.264			-0.229
		(0.385)			(0.410)
Betrayal averse			0.215		0.168
			(0.380)		(0.403)
Principled trustful				-0.0401	-0.0446
				(0.539)	(0.556)
Trust	0.335***	0.337***	0.337***	0.336***	0.338***
	(0.0666)	(0.0665)	(0.0662)	(0.0666)	(0.0666)
Expected trustworthiness	0.331***	0.330***	0.331***	0.331***	0.330***
	(0.0429)	(0.0429)	(0.0429)	(0.0430)	(0.0430)
Altruism	0.256***	0.254***	0.257***	0.256***	0.254***
	(0.0902)	(0.0898)	(0.0900)	(0.0900)	(0.0900)
Cooperation	0.108*	0.108*	0.107*	0.108*	0.108*
	(0.0590)	(0.0590)	(0.0590)	(0.0590)	(0.0591)
Risk propensity	-0.0831	-0.0826	-0.0856	-0.0828	-0.0841
	(0.110)	(0.110)	(0.110)	(0.110)	(0.110)
Extended sample	-0.0362	-0.0250	-0.0317	-0.0345	-0.0203
	(0.426)	(0.427)	(0.427)	(0.427)	(0.427)
Controls	Yes	Yes	Yes	Yes	Yes
PTs	Yes	Yes	Yes	Yes	Yes
Observations	1,017	1,017	1,017	1,017	1,017
R-squared	0.306	0.306	0.306	0.306	0.307

Notes: Robust standard errors in parentheses. Risk propensity types are defined as follows: *risk averse* types made a safe choice in both vignettes; *risk lover* types made a risky choice in both vignettes; *betrayal averse* types chose the risky option in the natural risk vignette and the safe option in the social risk vignette; *principled trustful* types chose the safe option in the natural risk vignette and the risky option in the social risk vignette. All columns include population weights adjusting the sample composition to the gender and age-group structure of Italian population as of 2017 by macroarea of residence. *** p<0.01, ** p<0.05, * p<0.1.

TABLE A20: The North-South divide in strength of family ties

	Family ties
South	0.235***
	(0.0853)
Extended sample	-0.0360
	(0.103)
Controls	Yes
PTs	Yes
Observations	1,016
R-squared	0.163

Notes: Robust standard errors in parentheses. The dependent variable is the first principal component extracted from three survey questions on family importance and responsibilities of family members. Included population weights adjusting the sample composition to the gender and age-group structure of Italian population as of 2017 by macroarea of residence. *** p<0.01, ** p<0.05, * p<0.1.

TABLE A21: The North-South divide in trustworthiness accounting for family ties

	Trustworthiness	
	(1)	(2)
South	-0.732**	-0.725**
	(0.355)	(0.353)
Family ties	-0.279*	-0.253*
	(0.143)	(0.143)
Trust	0.341***	0.374***
	(0.0667)	(0.0613)
Expected trustworthiness	0.331***	0.338***
	(0.0426)	(0.0427)
Altruism	0.256***	0.293***
	(0.0899)	(0.0922)
Cooperation	0.110*	
	(0.0588)	
Cond. cooperation		0.888**
		(0.419)
Risk propensity	-0.0938	-0.0485
	(0.110)	(0.109)
Extended sample	-0.0570	-0.0382
	(0.427)	(0.428)
Controls	Yes	Yes
PTs	Yes	Yes
Observations	1,016	1,016
R-squared	0.310	0.312

Notes: Robust standard errors in parentheses. Both columns include population weights adjusting the sample composition to the gender and age-group structure of Italian population as of 2017 by macroarea of residence. *** p<0.01, ** p<0.05, * p<0.1.

TABLE A22: The North-South divide in reciprocity accounting for family ties

	Reciprocity	
	(1)	(2)
South	-0.732**	0.107
	(0.349)	(0.296)
Send	1.421***	1.464***
	(0.0306)	(0.0350)
Send * South		-0.168**
		(0.0708)
Family ties	-0.279**	-0.279**
	(0.141)	(0.141)
Trust	0.341***	0.341***
	(0.0657)	(0.0657)
Expected trustworthiness	0.331***	0.331***
	(0.0420)	(0.0420)
Altruism	0.256***	0.256***
	(0.0886)	(0.0886)
Cooperation	0.110*	0.110*
	(0.0579)	(0.0579)
Risk propensity	-0.0938	-0.0938
	(0.108)	(0.108)
Extended sample	-0.0570	-0.0570
	(0.420)	(0.420)
Controls	Yes	Yes
PTs	Yes	Yes
Observations	11,176	11,176
R-squared	0.512	0.513

Notes: Robust standard errors in parentheses, clustered at the individual level. Both columns include population weights adjusting the sample composition to the gender and age-group structure of Italian population as of 2017 by macroarea of residence. *** p<0.01, ** p<0.05, * p<0.1.

TABLE A23: The North-South divide in trustworthiness accounting for internal migration patterns

	Trustworthiness					
	(1)	(2)	(3)	(4)	(5)	(6)
South (current residence)	-0.833**	-0.818**	-0.828**	-0.813**	-0.765**	-0.770**
	(0.361)	(0.358)	(0.362)	(0.359)	(0.386)	(0.383)
South to North migration (before childhood)			2.341	2.305		
			(2.301)	(2.336)		
South to North migration (after childhood)			0.665	0.702		
			(1.189)	(1.178)		
Migrated before childhood			0.130	0.150		
			(0.645)	(0.678)		
Migrated after childhood			-0.325	-0.298		
			(0.657)	(0.655)		
South to North migration (any age)	0.937	0.960				
	(1.052)	(1.047)				
Migrated at any age	-0.186	-0.161				
	(0.510)	(0.513)				
Trust	0.323***	0.356***	0.321***	0.354***	0.317***	0.350***
	(0.0673)	(0.0621)	(0.067)	(0.0618)	(0.0682)	(0.063)
Expected trustworthiness	0.322***	0.329***	0.321***	0.328***	0.326***	0.334***
	(0.0436)	(0.0436)	(0.0435)	(0.0435)	(0.0434)	(0.0433)
Altruism	0.269***	0.306***	0.273***	0.309***	0.274***	0.313***
	(0.091)	(0.0934)	(0.0906)	(0.0929)	(0.0912)	(0.0934)
Cooperation	0.108*		0.109*		0.114*	
	(0.0603)		(0.0601)		(0.0622)	
Cond. cooperation		0.886**		0.883**		0.926**
		(0.433)		(0.433)		(0.437)
Risk propensity	-0.0971	-0.0515	-0.092	-0.0465	-0.133	-0.083
	(0.112)	(0.112)	(0.112)	(0.112)	(0.111)	(0.111)
Extended sample	-0.0903	-0.0825	-0.0846	-0.0768	-0.217	-0.198
	(0.433)	(0.435)	(0.434)	(0.436)	(0.436)	(0.438)
Origin to current residence migration patterns (20 groups)	No	No	No	No	Yes	Yes
Controls	Yes	Yes	Yes	Yes	Yes	Yes
PTs	Yes	Yes	Yes	Yes	Yes	Yes
Observations	984	984	984	984	984	984
R-squared	0.304	0.306	0.305	0.307	0.327	0.329

Notes: Robust standard errors in parentheses. All columns include population weights adjusting the sample composition to the gender and age-group structure of Italian population as of 2017 by macroarea of residence. *** p<0.01, ** p<0.05, * p<0.1.

TABLE A24: The North-South divide in trustworthiness accounting for intergenerational transmission of preferences and migration

	Trustworthiness			
	(1)	(2)	(3)	(4)
North (residence)	0.599**	0.343	-0.127	-0.0888
	(0.270)	(0.342)	(0.433)	(0.442)
Parents from South		-0.617*	-1.075**	-1.085**
		(0.338)	(0.426)	(0.434)
Parents from South * North (residence)			1.238*	1.171*
			(0.695)	(0.699)
Migrated at any age				0.116
				(0.469)
Trust	0.306***	0.329***	0.327***	0.324***
	(0.0607)	(0.0675)	(0.0672)	(0.0677)
Expected trustworthiness	0.361***	0.329***	0.327***	0.321***
	(0.0374)	(0.0433)	(0.0429)	(0.0433)
Altruism	0.284***	0.261***	0.268***	0.275***
	(0.0841)	(0.0915)	(0.0910)	(0.0917)
Cooperation	0.127**	0.111*	0.105*	0.110*
	(0.0526)	(0.0601)	(0.0597)	(0.0604)
Risk propensity	-0.121	-0.0996	-0.0921	-0.0930
	(0.0880)	(0.112)	(0.111)	(0.112)
Extended sample	-0.155	-0.0745	-0.0659	-0.115
	(0.351)	(0.436)	(0.436)	(0.437)
Controls	Yes	Yes	Yes	Yes
PTs	Yes	Yes	Yes	Yes
Observations	1,406	993	993	980
R-squared	0.325	0.305	0.308	0.307

Notes: Robust standard errors in parentheses. All columns include population weights adjusting the sample composition to the gender and age-group structure of Italian population as of 2017 by macroarea of residence. *** p<0.01, ** p<0.05, * p<0.1.

FIGURE A1: Trust game in the Trustlab on-line platform

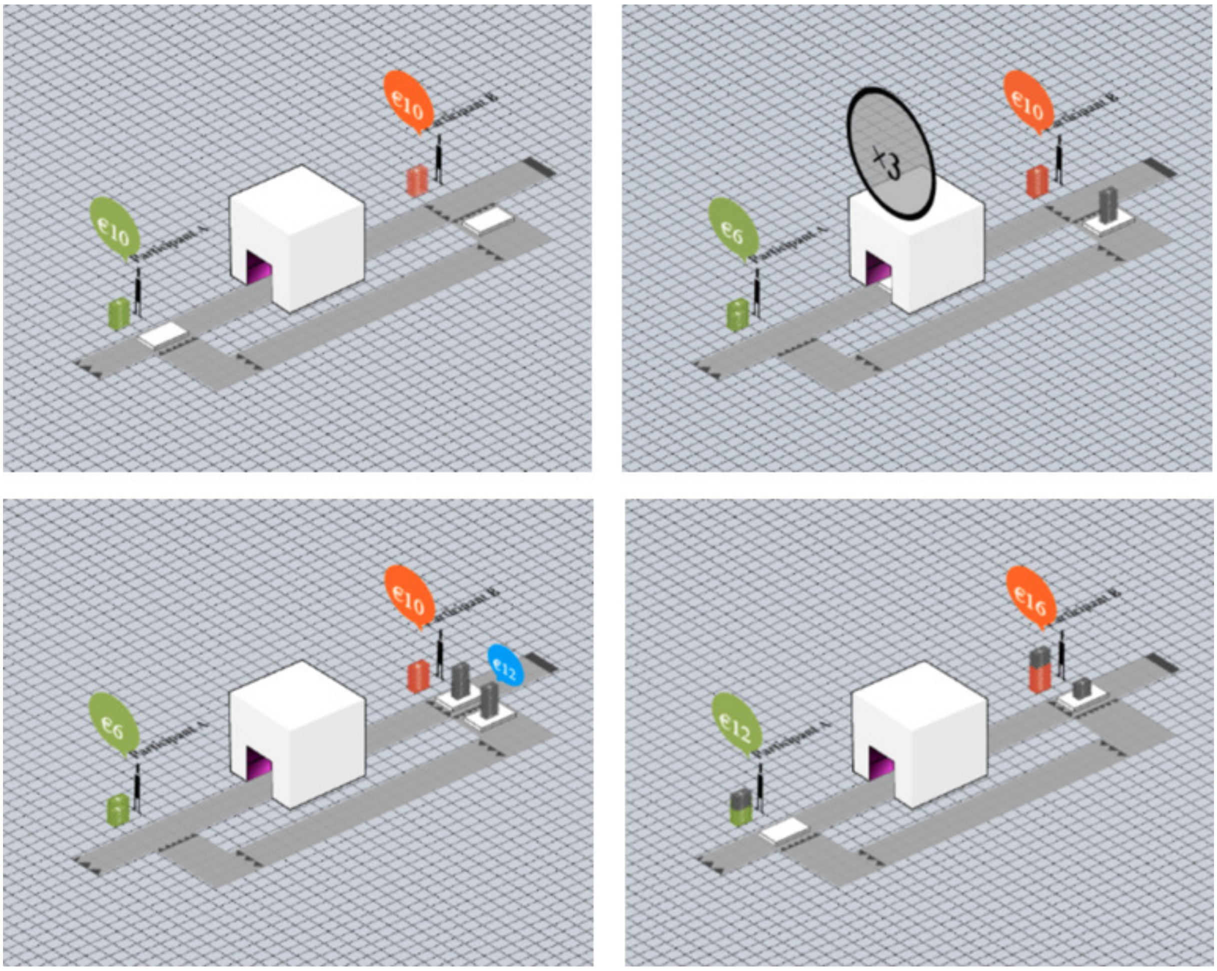

Notes: the screens show how respondents interact with the platform while playing the game. Player A chooses how much to transfer to player B (top left), then the amount is tripled (top right) and received by Player B (bottom left), who finally decides how much to return to player A.

FIGURE A2: Public goods game in the Trustlab on-line platform

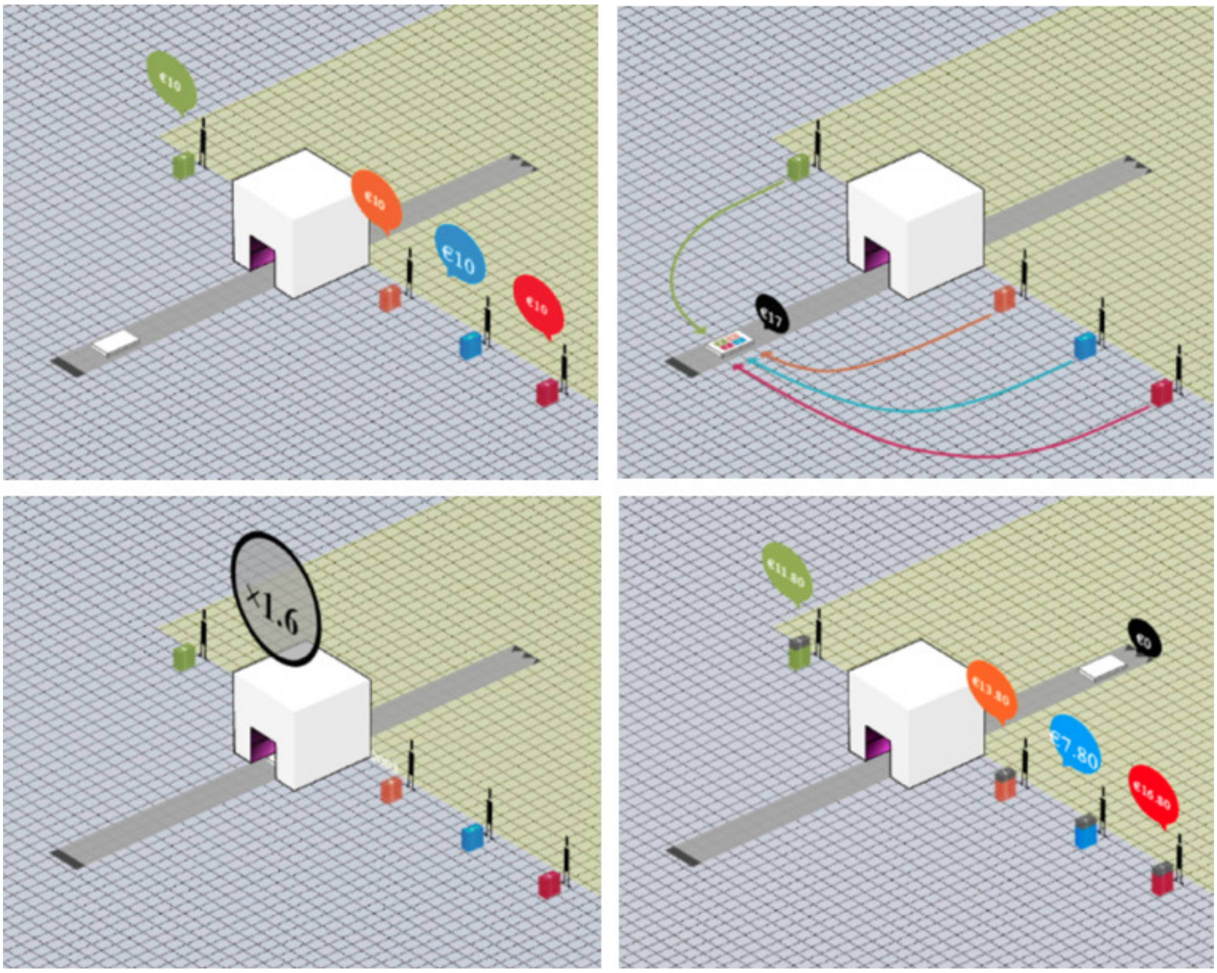

Notes: the screens show how respondents interact with the platform while playing the game. Each player chooses how much to contribute to the common project (top left), then the resources are collected into a common pool (top right) and multiplied by a factor of 1.6 (bottom left). The resulting new pool of resources is split equally among the four players (bottom right).

FIGURE A3: Personality differences across the Italian macroareas

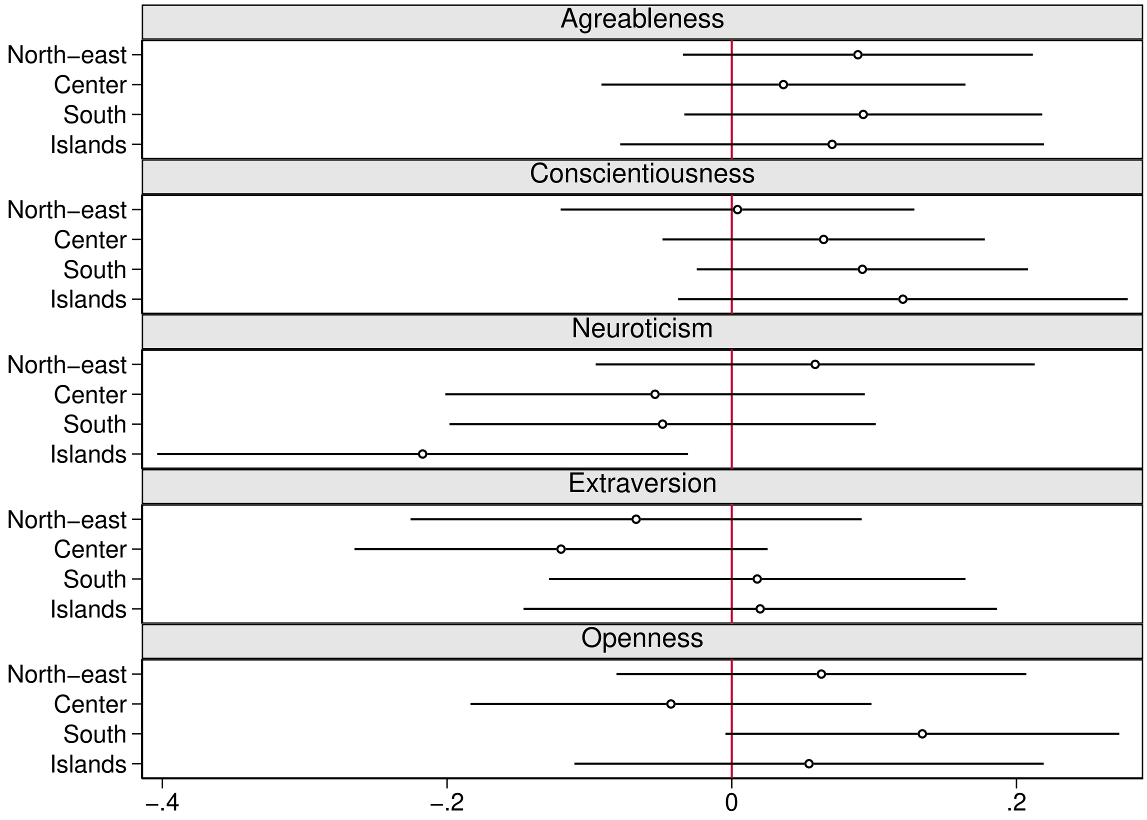

Notes: plot of coefficients of the Italian macroareas and their confidence intervals at the 95% level, from regressions of personality traits on socio-demographic characteristics of Trustlab respondents (the reference macroarea is North-West).

FIGURE A4: Theoretical trustor's payoffs based on empirical distribution of reciprocity

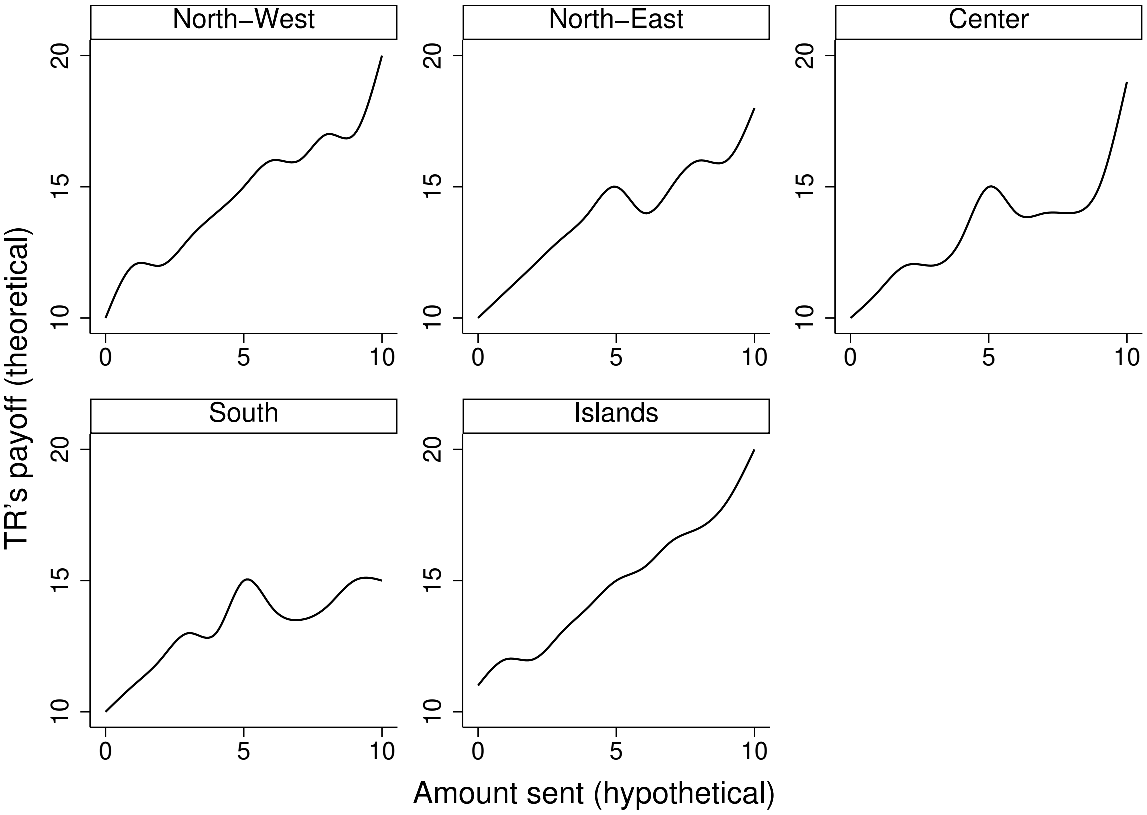

FIGURE A5: Distribution of trustor's offer

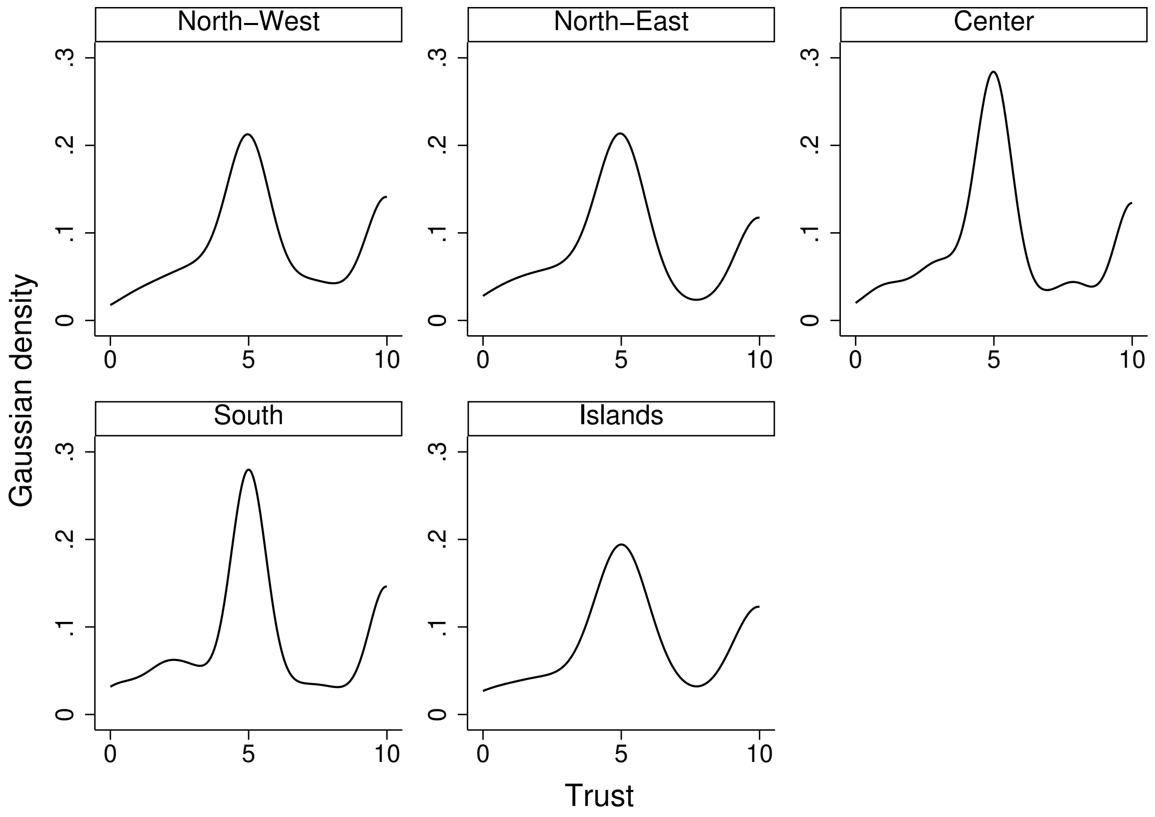

FIGURE A6: The North-South gap in conditional cooperation (public goods game)

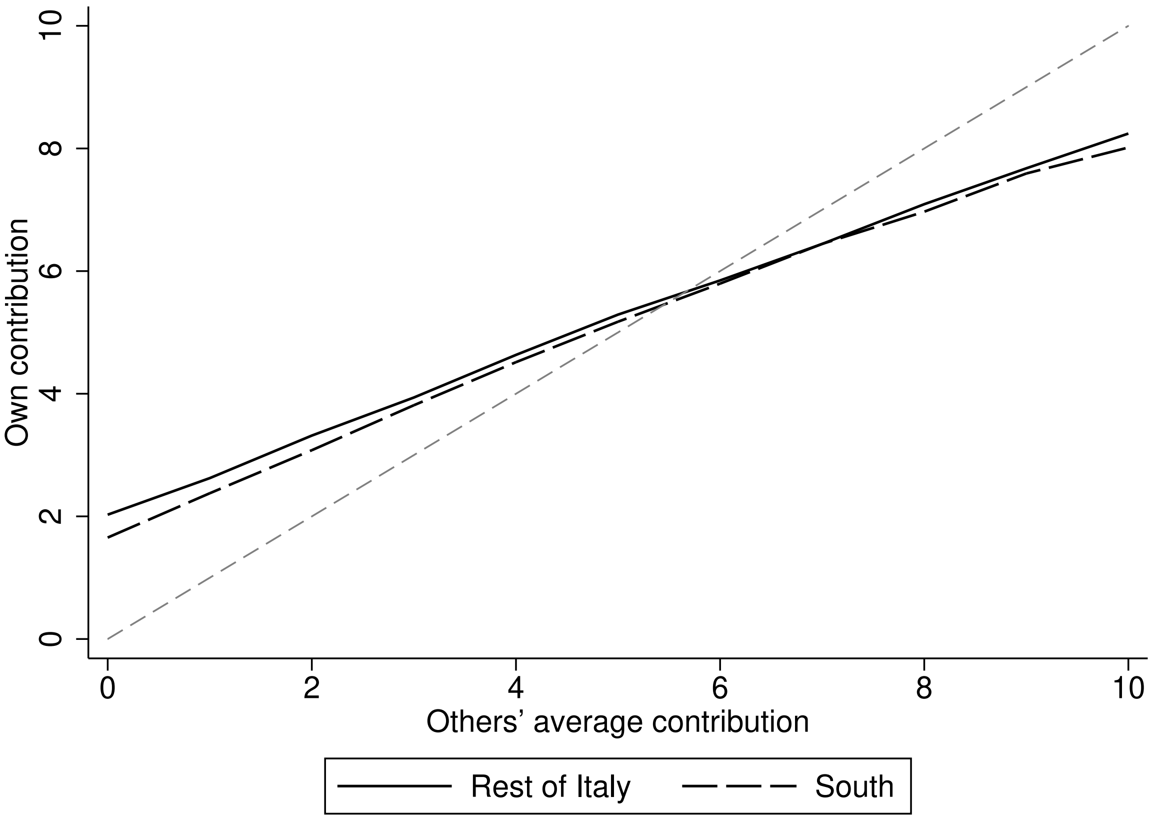

FIGURE A7: Distribution of risk-propensity types

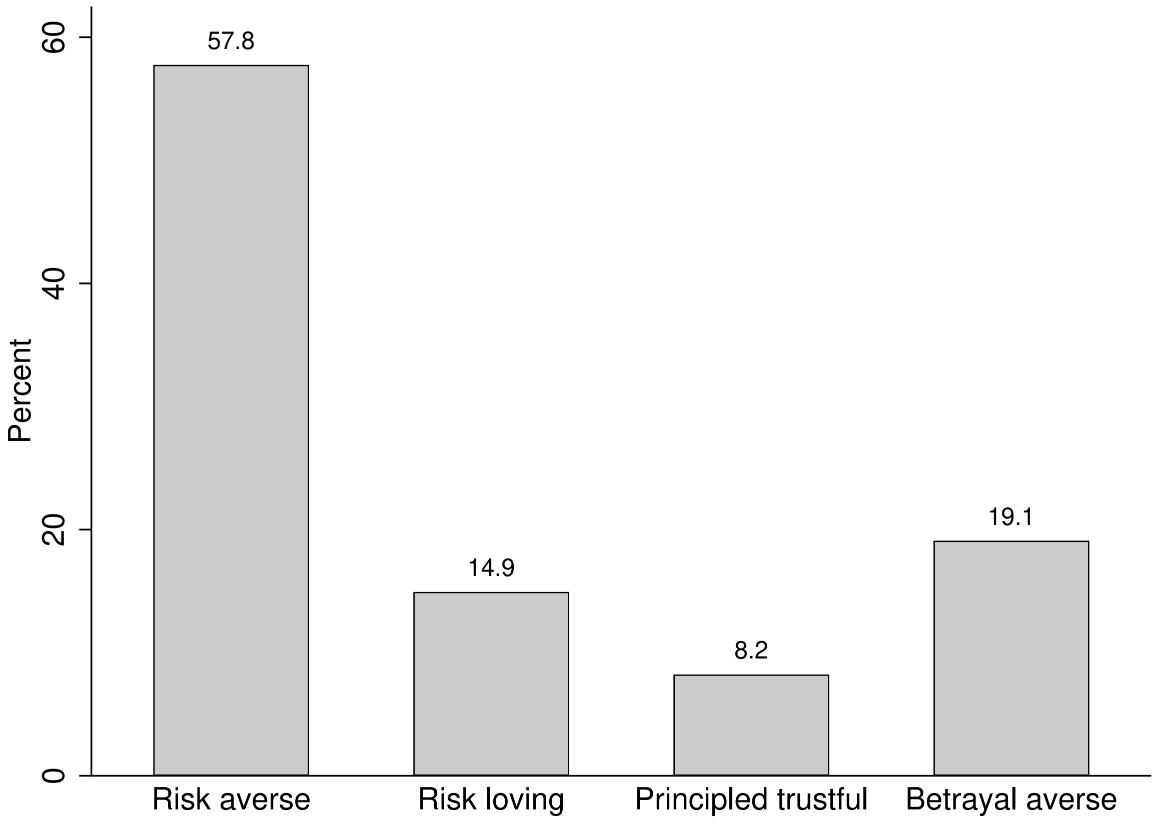

Notes: risk propensity types defined according to the choices taken in the social risk vignette (1) and in the natural risk vignette (2); risk loving types chose the risky option in both (1) and (2), risk averse types chose the safe option in both (1) and (2), principled trustful types chose the risky option in (1) and the safe option in (2), betrayal averse types chose the safe option in (1) and the risky option in (2).

FIGURE A8: Risk propensity types by macroareas

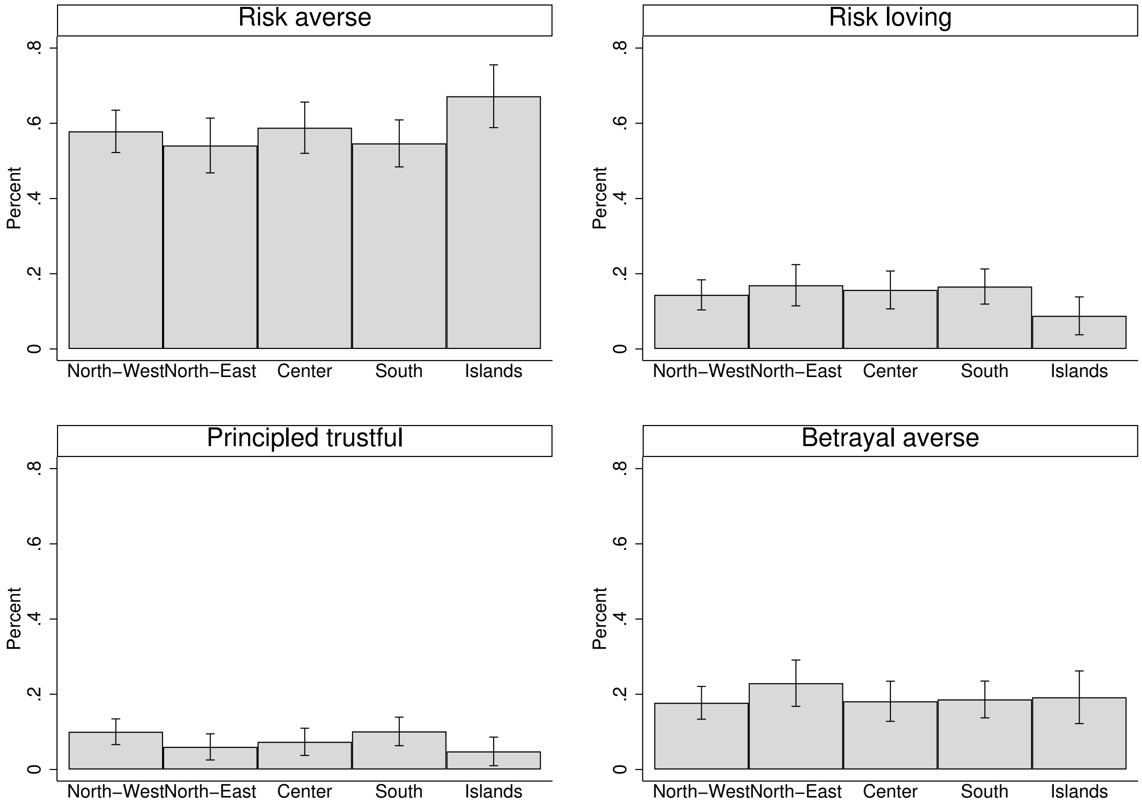

Notes: risk propensity types defined according to the choices taken in the social risk vignette (1) and in the natural risk vignette (2); risk loving types chose the risky option in both (1) and (2), risk averse types chose the safe option in both (1) and (2), principled trustful types chose the risky option in (1) and the safe option in (2), betrayal averse types chose the safe option in (1) and the risky option in (2).

FIGURE A9: Strength of family ties across Italian macroareas

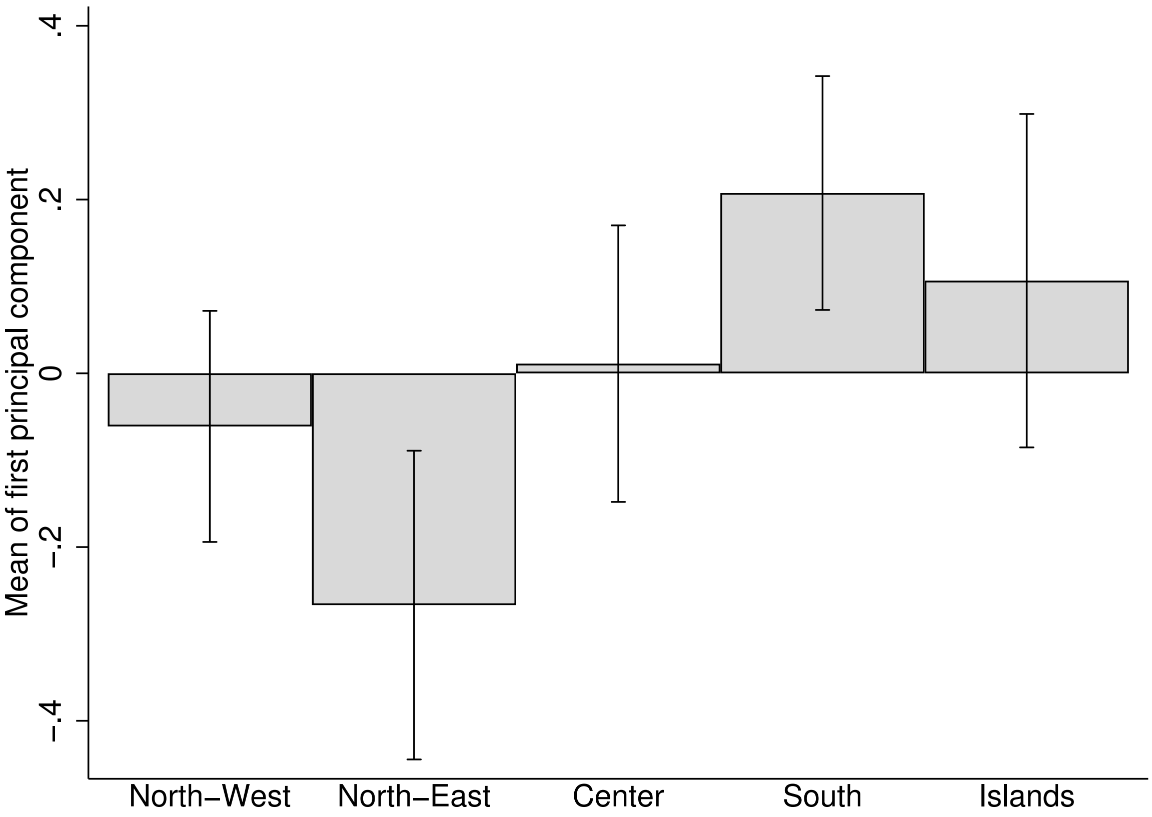

Notes: family ties measured as first principal component of three questions on the importance of the family, on children's responsibilities towards parents, and on parents' responsibilities towards children.

FIGURE A10: Migration patterns across Italian macroareas

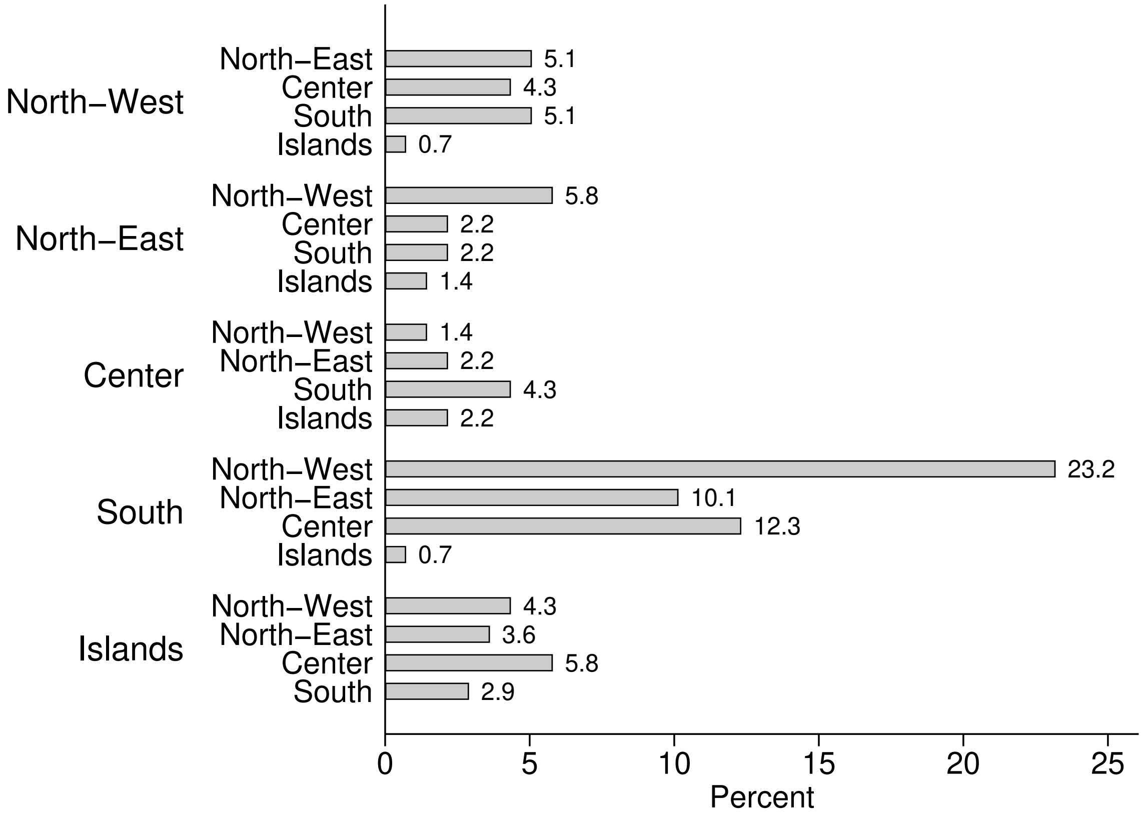

Notes: each bar shows the number of migrants from a macroarea of birth (outer categories) to a macroarea of residence (inner categories) as a percentage of total migrants in the sample.

FIGURE A11: Experimental trustworthiness by macroarea of residence (only respondents born in the South)

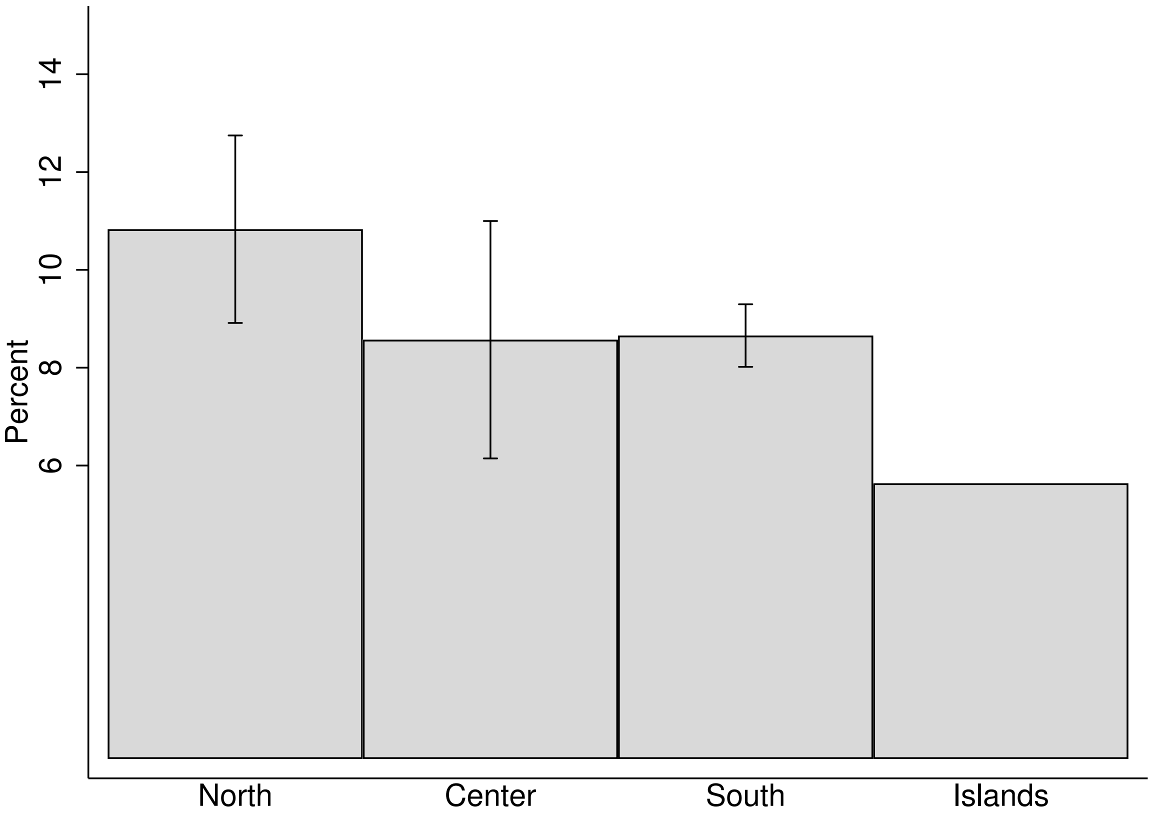

Appendix B

Trustlab is a platform developed online to measure trust and its determinants through behavioral games and survey questions in large samples representative of population in each country. The data collection was conducted by the OECD through a polling company responsible for the sampling design. All Trustlab participants are required to have access to the Internet through any device capable of loading the platform (specifically, over 90% of the Italian sample used a computer to participate (either desktop or laptop) while 7% used a tablet). Thus, Trustlab features by construction a non-random sampling design which addresses only people with Internet access.

The reliability and external validity of results from online surveys are often questioned. Estimation results will be biased as a consequence of scarce representation of population groups without Internet access (Bethlehem, 2010; Tourangeau, Conrad & Couper, 2013). Indeed, as shown in Table B1, use of the Internet is heterogeneous with respect to different strata of the Italian population in 2017. Overall, use of the Internet follows a North-South gradient, is more widespread among males, decreases with age while raising with education level, on top of being an increasing function of municipalities' population sizes; employed and self-employed Italians use the Internet more than unemployed and markedly more than inactive people (including housewives, students, retired and those seeking first job).

The heterogeneity in Internet use justifies concerns of sample selection. In fact, the geographical differences in social preferences estimated in the paper might be biased due to non-random sorting of population across Italy into Internet use and, in turn, into the Trustlab sample. In order to alleviate selection bias concerns, we re-estimate our models according to the two-step procedure discussed in Heckman (1979). The underlying regression equation mimics Equation 1. However, the social preference Y_i is observed only if respondent i can be selected into Trustlab, which means if she has access to the Internet, I_i. Selection into Internet access is modelled according to Equation B.1:

$$I_i = \begin{cases} 1 & \text{if } I_i^* > 0 \\ 0 & \text{if } I_i^* \leq 0 \end{cases} \tag{B.1}$$

Trustlab respondents all have Internet access, i.e. $I_i^* > 0$. The challenge is to supplement the Trustlab sample with respondents whose social preferences can not be observed because of no access to the Internet, i.e. $I_i^* \leq 0$. We choose a sample representative of the population that allows to discriminate between Internet users and non-users. An ideal candidate is the eighth round of the European Social Survey (ESS) administered in Italy in November 2017. The ESS conducts Computer Assisted Personal Interviews (CAPI) and collects measures of people's attitudes, beliefs and behaviors. It samples people older than fifteen years irrespective of the possession of a device or an Internet connection. In order to check whether ESS respondents have an Internet connection, we exploit a variable assessing the frequency of Internet use (respondents can answer they use the Internet never, only occasionally, a few times a week, most days, every day). While 84% of the ESS sample in the age range of Trustlab participants (18-64) consists of people who use the Internet at least a few times a week (who could have been selected for participation in Trustlab as inferred by frequent Internet use), the remaining 16% includes respondents who never use the Internet (8.7%) or use it only occasionally (7.3%): we supplement the Trustlab sample by the latter two groups of ESS respondents. Socio-demographic variables in the ESS are harmonised to be consistent with the ranges adopted in Trustlab (in particular gender, age, education, employment status, parents' education, size of residence area, macroarea of residence). Such variables are determinants of Internet use as shown in Table B1, and are used as controls in the baseline models.

In the first-step, we estimate the following selection Equation B.2 by probit:

$$I_i^* = \gamma_0 + \sum_k \gamma_k X_{ik}^* + \gamma_z Z_{j^*} + \eta_i \tag{B.2}$$

where I_i^* is a dummy variable taking value 1 if i is a Trustlab respondent (i.e. she has access to the Internet) and 0 if i is a ESS respondent using the Internet never or only occasionally (i.e. she does not have access to the Internet), X_{ik}^* is a set of k socio-demographic variables in common across ESS and Trustlab samples. Z_{j^*} is an exogenous instrument observed in each region j^* that influences the probability of Internet access without affecting directly social preferences. We impose exclusion restrictions to relax the assumptions on the functional form needed to identify the model correctly. γ_0 is a constant term, while η_i is an error term.

In the second-step, we estimate the following regression Equation B.3 by OLS:

$$Y_i = \delta_0 + \delta_m M_{j=South} + \sum_k \delta_k X_{ik} + \delta_h H_i + \zeta_i \tag{B.3}$$

where Y_i is the social preference of Trustlab's respondent i, M_j a macroarea dummy taking value 1 if respondent i lives in the South, X_{ik} is the set of k socio-demographic variables included in Equation 1 (inclusive of personality traits also), H_i is the non-selection hazard (inverse of the Mills' ratio) estimated from the parameter of the selection equation, and the associated coefficient δ_h summarizes the severity of selection bias. Imposing exclusion restrictions in the first-step reduces the collinearity problem between H_i and the other covariates. δ_0 is a constant term, while ζ_i is an error term.

Table B2 shows results from specifications of the selection equation obtained by varying the excluded instrument to improve robustness. Suitable exogenous determinants of Interet use come from the Italian Communications Regulatory Authority (AGCOM), which disseminates data on the supply of Internet within the country. While Column A is a specification without excluded instruments, the other columns augment the selection equation with the share of inhabitants served by high-speed broadband Internet (Column B), the share of households served by high-speed broadband Internet (Column C), the share of households not served by wireline network (Column D), the share of households served by ADSL technology (Column E). Across all columns these region-level variables significantly affect the probability of using the Internet in the expected direction. Overall, females are more likely to use the Internet. As for age, we observe a decreasing trend in Internet use. Having achieved an education level higher than secondary also significantly increases the probability of Internet use. The same holds true when looking at the effect of parental education (especially fathers'). Self-employed, unemployed and inactive are significantly less likely to use the Internet compared to employed people. A differential emerges across macroareas in that respondents from Southern and North-eastern Italy have a lower probability of Internet use compared to their North-western counterparts. The coefficients on the size of residence area show surprisingly different signs than expected: compared to people in villages, those in metropolitan areas of any size are significantly less likely to use the Internet, while the opposite is true for respondents in rural

areas (although the matching of this variable to Trustlab's is not as straightforward as for the other covariates). Size of the household is not an important predictor of Internet use.

The panels in Table B3 show the results of the second-step equations for each specification of the selection equation highlighted above (Panel A to E). By estimating the non-selection hazard in the first-step, we can assess whether and to what extent the selection bias affects the correlations between the South macroarea and social preferences measured in Trustlab, conditional on baseline socio-demographic controls and personality traits. Sample selection does not emerge as a severe concern, because the non-selection hazard never gets close to significance in any of the specifications, with the exception of Column 6 in Panel D. The findings highlighted in the paper are fully robust to selection: Southerners are significantly less trustworthy than respondents from the rest of Italy (the coefficient on South is underestimated in the baseline regression of Table 7), while they are similar in terms of other social preferences. Estimation by maximum likelihood provides very similar results (available upon request).

Despite no evidence of selection induced by differences in Internet access across Italy, one may wonder whether the online nature of Trustlab experiments altered the elicitation of social preferences in a non-homogeneous way across the country. To address this concern we again exploit the eight round of the ESS because it provides a representative sample of the Italian population not recruited online. By taking advantage of a question about generalized trust available in both surveys, we are able to establish the extent of selection bias in the measurement of trust brought about by online as opposed to face-to-face sample recruitment. The question asks *"Generally speaking, would you say that most people can be trusted, or that you can't be too careful in dealing with people?"* and respondents answer on a 0-10 scale, where 0 means *"You can't be too careful"* and 10 means *"Most people can be trusted"*. Although a question identical in wording is asked in the EVS, the binary answer range does not allow a direct comparison with Trustlab.

Table B4 summarizes average trust levels by region in the two surveys. Across columns, we impose a restriction on the ESS sample to the age range of respondents in Trustlab (18

to 64 years - Columns 2 and 4), include the observations coming from the boost sample in Trustlab (Columns 6 and 8), use post-stratification weights from the ESS (Columns 3 and 4), use weights adjusting statistics in Trustlab to the regional sex-age composition in 2017 in Italy (Columns 7 and 8). Despite overall lower levels of trust in the ESS, the regional distributions appear similar. The intuition is confirmed in Table B5, which shows correlations between each ESS and Trustlab regional distribution of trust. Correlation coefficients are large and significant.

References

Bethlehem, J. (2010). Selection bias in web surveys. *International Statistical Review*, *78*(2), 161–188.

Heckman, J. J. (1979). Sample selection bias as a specification error. *Econometrica*, *47*(1), 153–161.

Tourangeau, R., Conrad, F. G., & Couper, M. P. (2013). *The science of web surveys.* Oxford: Oxford University Press.

TABLE B1: Use and frequency of Internet use in Italy by socio-demographic characteristics

Variable		Use of Internet		Frequency of Internet Use			
		Yes	No	Every day	Few times a week	Few times a month	Few times a year
Place of residence	North-West	69.1	29.8	50	15.7	2.5	0.9
	North-East	68	31.1	50.2	15.3	1.8	0.7
	Center	67.8	31.5	50.5	14.8	1.7	0.8
	South	59.1	40	41.7	14.6	2.1	0.7
	Islands	59.6	39	44.1	13	1.8	0.8
Gender	Male	69.5	29.6	50.8	15.6	2.1	0.9
	Female	61.3	37.7	44.5	14.2	1.9	0.7
Age	18-24	92.6	6	81.5	9.9	1.1	0.1
	25-34	89.8	8.7	72.9	15	1.5	0.4
	35-44	85.7	13.5	65.6	17.5	1.8	0.7
	45-54	77.6	21.6	55.6	18.4	2.5	1.1
	55-64	62.1	37.1	41.1	17.8	2.2	1.1
Education	Lower secondary school	63.9	35	42.7	17.7	2.4	1.1
	Higher secondary school	84.5	14.6	65.1	16.7	2.1	0.7
	Under/Post-graduate	91.6	7.4	79.8	10.8	0.7	0.3
Employment status	Employed	88.8	10.6	71.7	14.9	1.5	0.6
	Self-employed	78	20.9	56.8	17.8	2.3	1
	Unemployed	74.6	24	51.1	19.6	2.9	1.1
	Inactive	56	42.5	42.6	11.1	1.7	0.7
Size of residence municipality (number of citizens)	Up to 2,000	55.3	43.7	38.7	13.4	2.2	1
	From 2,001 to 10,000	63	36	44.8	14.7	2.7	0.8
	From 10,001 to 50,000	63.9	35.2	46.5	15	1.7	0.8
	From 50,001	65.9	33.2	48.7	14.7	1.7	0.8

Notes: statistics retrieved from the Istat data wharehouse, extracted from the *Multipurpose survey on households: aspects of daily life* (2017).

TABLE B2: Probability of Internet use across population strata, without and with variables excluded from the selection equation

	Use the Internet				
	(1)	(2)	(3)	(4)	(5)
Share of inhabitants served with speed in range 100–1000 Mbps		2.590***			
		(0.763)			
Share of households served with speed in range 100–1000 Mbps			2.943***		
			(0.778)		
Share of households not served by wireline network				-10.37***	
				(2.358)	
Share of households served by ADSL technology					10.08**
					(4.031)
Female respondent	0.235**	0.260**	0.262**	0.237**	0.244**
	(0.112)	(0.113)	(0.113)	(0.113)	(0.112)
Age 25-34	-0.690**	-0.703**	-0.699**	-0.628*	-0.648*
	(0.350)	(0.353)	(0.353)	(0.356)	(0.351)
Age 35-44	-0.806**	-0.860***	-0.863***	-0.813**	-0.795**
	(0.326)	(0.327)	(0.327)	(0.329)	(0.325)
Age 45-54	-0.920***	-0.960***	-0.962***	-0.901***	-0.883***
	(0.312)	(0.313)	(0.314)	(0.316)	(0.312)
Age 55-64	-1.187***	-1.198***	-1.197***	-1.153***	-1.148***
	(0.312)	(0.313)	(0.314)	(0.316)	(0.312)
People in household	0.0595	0.0600	0.0604	0.0644	0.0598
	(0.0455)	(0.0459)	(0.0460)	(0.0461)	(0.0457)
Some college/non tertiary	1.636***	1.604***	1.605***	1.686***	1.634***
	(0.317)	(0.310)	(0.310)	(0.321)	(0.318)
Tertiary education	1.404***	1.457***	1.464***	1.465***	1.398***
	(0.228)	(0.234)	(0.236)	(0.236)	(0.230)
Self-employed	-0.487***	-0.512***	-0.513***	-0.487***	-0.492***
	(0.169)	(0.170)	(0.170)	(0.170)	(0.169)
Unemployed	-0.345**	-0.346**	-0.346**	-0.351**	-0.339**
	(0.160)	(0.162)	(0.162)	(0.162)	(0.161)
Inactive	-0.681***	-0.740***	-0.747***	-0.712***	-0.692***
	(0.136)	(0.138)	(0.138)	(0.138)	(0.136)
Rural area	0.645***	0.700***	0.709***	0.724***	0.667***
	(0.189)	(0.190)	(0.191)	(0.195)	(0.191)
Small/medium metro. area	-0.649***	-0.713***	-0.718***	-0.688***	-0.655***
	(0.128)	(0.131)	(0.131)	(0.129)	(0.128)
Large metropolitan area	-0.491***	-0.605***	-0.627***	-0.594***	-0.547***
	(0.172)	(0.177)	(0.178)	(0.176)	(0.174)
Educated father	0.873***	0.864***	0.865***	0.899***	0.889***
	(0.178)	(0.180)	(0.180)	(0.183)	(0.180)
Educated father	0.717***	0.708***	0.709***	0.730***	0.718***
	(0.210)	(0.211)	(0.212)	(0.216)	(0.212)
North-East	-0.350**	-0.200	-0.175	-0.127	-0.191
	(0.156)	(0.164)	(0.165)	(0.166)	(0.170)
Center	0.00238	-0.0278	-0.00736	0.275	0.0266
	(0.160)	(0.161)	(0.161)	(0.173)	(0.161)
South	-0.501***	-0.551***	-0.505***	-0.265*	-0.496***
	(0.144)	(0.146)	(0.146)	(0.156)	(0.145)
Islands	-0.00658	-0.237	-0.205	0.131	-0.0692
	(0.190)	(0.201)	(0.197)	(0.194)	(0.192)
Observations	1,246	1,246	1,246	1,246	1,246
Pseudo R^2	0.388	0.397	0.399	0.403	0.393

Notes: *** p<0.01, ** p<0.05, * p<0.1.

TABLE B3: Second-step equations from first-step equations without and with excluded variables

	GTQ	Trust	Trustworthiness	Expected trustworthiness	Altruism	Cooperation	Conditional cooperation	Risk propensity
	(1)	(2)	(3)	(4)	(5)	(6)	(7)	(8)
A. Excluded variable: *None*								
South	-0.200	-0.0896	-0.973**	-0.143	-0.0386	-0.0506	-0.0132	0.202
	(0.166)	(0.242)	(0.430)	(0.456)	(0.190)	(0.236)	(0.0339)	(0.132)
Non-selection hazard	-0.523	0.344	0.432	0.749	0.265	-0.681	-0.0102	-0.329
	(0.404)	(0.590)	(1.049)	(1.111)	(0.464)	(0.574)	(0.0826)	(0.321)
B. Excluded variable: *Share of inhabitants served with speed in range 100–1000 Mbps*								
South	-0.228	-0.107	-0.975**	-0.179	-0.0211	-0.0382	-0.0106	0.197
	(0.165)	(0.241)	(0.428)	(0.454)	(0.189)	(0.235)	(0.0337)	(0.131)
Non-selection hazard	-0.298	0.528	0.479	1.138	0.120	-0.840	-0.0352	-0.307
	(0.393)	(0.574)	(1.020)	(1.080)	(0.451)	(0.558)	(0.0803)	(0.312)
C. Excluded variable: *Share of households served with speed in range 100–1000 Mbps*								
South	-0.232	-0.108	-0.972**	-0.187	-0.0202	-0.0355	-0.0107	0.197
	(0.165)	(0.241)	(0.427)	(0.454)	(0.189)	(0.235)	(0.0336)	(0.131)
Non-selection hazard	-0.265	0.540	0.454	1.225	0.113	-0.875	-0.0349	-0.310
	(0.391)	(0.570)	(1.013)	(1.073)	(0.448)	(0.554)	(0.0798)	(0.310)
D. Excluded variable: *Share of households not served by wireline network*								
South	-0.247	-0.114	-0.952**	-0.207	-0.0405	-0.0282	-0.0112	0.199
	(0.164)	(0.240)	(0.426)	(0.453)	(0.189)	(0.235)	(0.0336)	(0.131)
Non-selection hazard	-0.121	0.593	0.263	1.395	0.300	-0.932*	-0.0300	-0.324
	(0.375)	(0.547)	(0.973)	(1.029)	(0.430)	(0.531)	(0.0766)	(0.297)
E. Excluded variable: *Share of households served by ADSL technology*								
South	-0.215	-0.115	-0.971**	-0.147	-0.0458	-0.0357	-0.0134	0.201
	(0.166)	(0.242)	(0.429)	(0.455)	(0.190)	(0.236)	(0.0338)	(0.132)
Non-selection hazard	-0.388	0.560	0.414	0.783	0.325	-0.805	-0.00868	-0.326
	(0.389)	(0.567)	(1.008)	(1.068)	(0.446)	(0.552)	(0.0794)	(0.308)
Controls	Yes	Yes	Yes	Yes	Yes	Yes	Yes	Yes
PTs	Yes	Yes	Yes	Yes	Yes	Yes	Yes	Yes
Obs. selected	975	979	979	979	979	979	979	979

Notes: Heckman's efficient standard errors in parentheses. *** p<0.01, ** p<0.05, * p<0.1.

TABLE B4: Average levels of trust by region in the ESS-8 and in Trustlab

	European Social Survey				Trustlab			
	NW UR	NW R	W UR	W R	NW NB	NW B	W NB	W B
	(1)	(2)	(3)	(4)	(5)	(6)	(7)	(8)
Piemonte	4.46	4.52	4.45	4.53	5.39	5.09	5.48	5.32
Valle d'Aosta	5.52	5.58	5.53	5.59	8.00	8.00	8.00	8.00
Lombardia	4.85	5.02	4.83	5.03	5.27	5.15	5.48	5.39
Trentino-Alto Adige	5.06	4.83	5.11	4.93	5.45	5.53	6.02	5.92
Veneto	4.62	4.63	4.60	4.61	5.12	5.15	5.18	5.20
Friuli-Venezia Giulia	3.00	2.89	2.98	2.86	4.88	4.65	4.54	4.46
Liguria	5.10	4.92	5.08	4.88	5.43	5.25	5.65	5.46
Emilia-Romagna	4.89	4.94	4.91	4.96	4.86	4.56	5.14	4.97
Toscana	4.57	4.84	4.49	4.78	5.03	4.90	5.18	5.10
Umbria	5.43	5.33	5.44	5.35	4.63	5.36	5.45	5.77
Marche	4.24	4.08	4.26	4.06	4.39	4.62	4.13	4.31
Lazio	4.87	5.09	4.83	5.06	4.69	4.66	4.62	4.61
Abruzzo	4.93	5.23	4.94	5.20	4.65	4.65	5.25	5.08
Molise	-	-	-	-	5.67	4.50	5.63	4.66
Campania	4.52	4.68	4.48	4.64	4.74	4.63	5.03	4.90
Puglia	4.19	4.30	4.13	4.25	5.04	5.03	5.10	5.10
Basilicata	3.81	4.00	3.74	3.93	3.50	3.50	3.34	3.34
Calabria	4.38	4.46	4.35	4.44	3.96	4.05	4.52	4.44
Sicilia	3.95	4.13	3.87	4.02	4.96	4.83	4.70	4.66
Sardegna	5.33	5.14	5.37	5.17	5.04	4.44	5.28	4.95

UW: unweighted; W: weighted; UR: without age restriction (18-64); R: with age restriction (18-64); UB: without boost; B: with boost.

TABLE B5: Correlation matrix of the regional distributions of trust in the ESS-8 and in Trustlab

	ESS (UW; UR)	ESS (UW; R)	ESS (W; UR)	ESS (W; R)
Trustlab (UW; NB)	0.4637**	0.4292*	0.4678**	0.4391*
Trustlab (UW; B)	0.5077**	0.4699**	0.5108**	0.4792**
Trustlab (W; NB)	0.6653***	0.6257***	0.6706***	0.6408***
Trustlab (W; B)	0.6609***	0.6192***	0.6654***	0.6335***

UW: unweighted; W: weighted; UR: without age restriction (18-64); R: with age restriction (18-64); UB: without boost; B: with boost.

Appendix C

Supplementary Materials

A. Experimental instructions

Welcome!

Our research team (including researchers from Sciences Po Paris, Brown University and Kiel University) invites you to participate in a quick online study on decision-making.

Study information

The aim of this study is to learn more about how we as human beings behave – how do we make decisions? How do we interact with one another when faced with different choices? How do we feel about the people and institutions around us?

To find this out, you will be participating in different tasks. In the first part, you will participate in four simple tasks, in anonymous interaction with one or more other people. In the second part, you are going to sort different sets of words. In the third part, we ask you to answer a few questions about yourself and your opinions.

The whole study should take you about 30 minutes. Note that you should complete this study in one sitting, without any extensive period of inactivity. For best results, minimize distractions and close other programs.

You can participate in the study via your laptop computer or tablet (we support recent iPads). If you are having trouble accessing the platform, we advise you to switch to Google Chrome. If problems persist, please contact GMI, specifying your device model and browser.

Payment

By participating in the study's tasks, you can earn up to €40.

This amount will depend on the decisions you make together with the other participants during the study's tasks. At the end of the study, one of the several tasks you have completed will be randomly selected. **The amount of money you will receive will correspond to your earnings in this selected task. Your decisions will also**

affect the earnings that other people will receive!

You will receive your money at the end of the study via Paypal. Your payment will be processed after your decisions and those of other participants are collected. Because other participants may not be online at the same time as you, the calculation of your earnings may take up to 48 hours.

Data protection

The data gathered in this study is subject to national privacy protocols. We will use it for research purposes only.

Section One: Tasks

We will start by giving you four tasks. Note that each task may include several different decisions. This is the part of the study that will allow you to earn additional money. Each of these decisions may determine your final payments.

At the beginning of each task, you may be grouped with other study participants. **All participants in this study are from Italy like you**.

In each task, the other participants you are grouped with will be different: the same person will never be in your group more than once.

How will your earnings be calculated?

Your earnings in each task will depend on your and the other participants' decisions.

At the end of the study, one of the four tasks you have completed will be randomly selected. The amount of money you will receive will correspond to your earnings in one of the decisions in this selected task.

In short, each task may determine your final payoff!

A.1. Trust game

Task One: Introduction

In the first task, **two people participate**: Participant A and Participant B. As mentioned before, this other participant also lives in **Italy**. **These are the rules of the task**:

- At the beginning of the task, both participants receive €10.
- Participant A has the option to transfer none, part or all of his or her €10 to Participant B.
- Whatever amount Participant A sends is **multiplied by 3**.
- Participant B, after receiving the transfer of Participant A, has to decide how much money, if any, he or she wants to send back to Participant A.

You are asked to make decisions in both role A and B. Which role you will be assigned to for payment will be determined randomly.
In either case, your interaction will be with a person who gets randomly assigned to the other role.

Task One: Test Simulation

This is not the real task yet, but a simulation to help you understand the rules better.
You can use the test screen below to experiment with the different choices of the two participants. Between each test, click the *'reset to zero'* button below to reset the calculator.
Whenever you are ready to proceed to the real task, click *'Next'*.

Task One: Real Task (Participant A)

Now the real task 1 starts. Once you have made your decision and clicked the *'Next'* button, you cannot return to this screen.
Suppose you are selected to be in the role of **Participant A**.
You have €10 in your possession. **How much (if any) do you want to send to Participant B**?

Please enter a number from 0 to 10.

Task One: Real Task (Participant B)

Now, suppose you are selected to be in the role of **Participant B**.

On this screen you will make the decisions that will count if you are selected for that role. Once you have made your decision and clicked the "Next" button, you cannot return to this screen. As always, your initial endowment is €10.

Remember that Participant A also starts with an endowment of €10.

If Participant A sends you any of the amounts listed in the table below, **how much money (if any) do you want to send back to Participant A**?

All of your choices below can impact how much money you and the other participant will receive at the end of the study.

1. If Participant A sends you €0, your total endowment is now €10. How much will you send back to Participant A.
2. If Participant A sends you €1, your total endowment is now €13. How much will you send back to Participant A.
3. If Participant A sends you €2, your total endowment is now €16. How much will you send back to Participant A.
4. If Participant A sends you €3, your total endowment is now €19. How much will you send back to Participant A.
5. If Participant A sends you €4, your total endowment is now €22. How much will you send back to Participant A.
6. If Participant A sends you €5, your total endowment is now €25. How much will you send back to Participant A.
7. If Participant A sends you €6, your total endowment is now €28. How much will you send back to Participant A.

8. If Participant A sends you €7, your total endowment is now €31. How much will you send back to Participant A.

9. If Participant A sends you €8, your total endowment is now €34. How much will you send back to Participant A.

10. If Participant A sends you €9, your total endowment is now €37. How much will you send back to Participant A.

11. If Participant A sends you €10, your total endowment is now €40. How much will you send back to Participant A.

Task One: Expectations

You have just had made decisions as Participant A and Participant B. The following question is about your expectations of other people's decisions. You are not actually deciding as Participant A or Participant B, and this decision will not affect your earnings. We want you to imagine the following scenario:

Imagine you sent €5, so Participant B receives €15, making his or her total budget €25. Participant B has no information about your identity. What amount would you expect Participant B to return to you?

Please enter a number from 0 to 25.

Thank you very much for entering your choice.

We have recorded your decision. Now, please proceed to the second task.

A.2. Public goods game

Task Two: Introduction

In the second task, there are groups of **4 participants** (yourself and 3 other people). Remember, the participants in this group are different from the person you interacted with in the previous task. However, they all live in Italy.

These are the rules:

- At the beginning, each group member has €10.
- Every group member has to choose how much of this €10 he or she wants to keep and how much he or she wants to transfer into a **joint project**.
- The total amount transferred to the joint project is **multiplied by 1.6**.
- At the end, the money in the joint project will be re-divided and **split equally between all 4 group members** (including yourself).

Task Two: Test Simulation This is not the real task yet, but a simulation to help you understand the rules better. You can use the test screen below to experiment with the different choices of the four participants.
Whenever you are ready to proceed to the real task, click *'Next'*.

Task Two: Real Task
Now the real task starts. Once you have made your decision and clicked the *'Next'* button, you cannot return to this screen.
You have €10 in your possession. You may choose to keep this money, or choose to invest some (or all) of it in the joint project.
How much (if any) do you want to transfer to the project?

Task Two: Real Task (continued)
Now imagine that this time, you find out how much money the other three members of your group are investing in the joint project.
All of your choices below can impact how much money you will receive at the end of the study.
Please indicate how much (if any) you would like to transfer to the joint project:

1. if on average, each of the other group members contributes €0.
2. if on average, each of the other group members contributes €1.
3. if on average, each of the other group members contributes €2.

4. if on average, each of the other group members contributes €3.
5. if on average, each of the other group members contributes €4.
6. if on average, each of the other group members contributes €5.
7. if on average, each of the other group members contributes €6.
8. if on average, each of the other group members contributes €7.
9. if on average, each of the other group members contributes €8.
10. if on average, each of the other group members contributes €9.
11. if on average, each of the other group members contributes €10.

Thank you very much for entering your choice.
We have recorded your decision. Again, your payoff will depend on the actions of the other participants. Now, please proceed to the third task.

A.3. Dictator game

Task Three: Introduction The third task involves **two participants** -– Participant A and Participant B.
Remember, the other participant is different from the ones you interacted with in the previous two tasks. However, he or she also lives in Italy.
These are the rules:

- At the beginning, Participant A receives €10.
- Participant B does not receive any money — he or she has €0.
- Participant A must now decide if he or she wants to transfer any of his or her €10 to Participant B.
- This transfer is not multiplied by any number and Participant B cannot transfer any amount back to Participant A.

Your role (Participant A or Participant B) will be determined later. We ask you to make a choice as A in case this is your role. B has no decision to make. Remember that someone will be assigned to role B and that person's payment will be affected by your decision as A.

Because this task is simple, there will be no simulator to test out different choices.

Task Three: Real Decision

This is the real third task. Once you have made your decision and clicked the "Next" button, you cannot return to this screen.

Suppose that you are selected to be in the role of Participant A.

You have €10 in your possession. **How much (if any) do you want to transfer to Participant B**?

Thank you very much for entering your choice.

We have recorded your decision. Now, please proceed to the fourth task.

A.4. Lottery choice

Task Four: Introduction

In this task you have the option to choose from six different gambles. In each gamble, you can win one out of two amounts.

You must select **one and only one** of these gambles.

Each gamble has two possible outcomes: outcome A and outcome B. Only one of these outcomes will occur.

The gamble works as a random draw, comparable to a coin toss. As in a coin toss, each possible outcome has a 50% chance of occurring.

Your compensation for this part of the study will be determined by:

1. **Which of the six gambles you select**. This is your choice.
2. **Which of the two possible outcomes occur**. This is determined by chance. The random draw is conducted by our computer. Either outcome has the same

probability of occurring.

The gamble selection table below shows your possible options. You will be asked to choose one of these gambles.

Gamble	Outcome	Payoff	Probabilities	Choice
1	A	8	50%	
	B	8	50%	
2	A	7	50%	
	B	10	50%	
3	A	6	50%	
	B	12	50%	
4	A	5	50%	
	B	14	50%	
5	A	4	50%	
	B	16	50%	
6	A	1	50%	
	B	19	50%	

Examples: For instance, if you choose Gamble 2, you will earn **€€7** if outcome A occurs, or **€10** if outcome B occurs.
If you choose Gamble 5, you will earn **€4** if outcome A occurs, or **€16** if outcome B occurs.
If you choose Gamble 1, you will earn **€8**, regardless of which outcome occurs.

Task Four: Real Task Now the real task four starts. Once you have made your decision and clicked the "Next" button, you cannot return to this screen.
These are the six gambles from which you can choose. If this task is chosen for payment, then your earnings will depend on the gamble you choose and the outcome of the gamble. Please select the gamble of your choice.

Thank you very much for your participation in this study! We have recorded your decisions. Your final earnings will depend on the decisions of the other participants that you interact with in this study.
Because the other participants may not be online at the same moment, your earnings will

Gamble	Outcome	Payoff	Probabilities	Choice
1	A	8	50%	⬭
	B	8	50%	
2	A	7	50%	⬭
	B	10	50%	
3	A	6	50%	⬭
	B	12	50%	
4	A	5	50%	⬭
	B	14	50%	
5	A	4	50%	⬭
	B	16	50%	
6	A	1	50%	⬭
	B	19	50%	

be calculated once you are matched with another participant or a group of participants. It may take up to 48 hours to calculate your earnings.
You will be able to check your final earnings using the following link (the access link to view your earnings will be available for up to 5 days after you close this screen. If you do not navigate to the payoff screen, your earnings will be transferred to you automatically):

[LINK]

Please store this link somewhere on your computer. Once you click *'Next'*, you will not be able to return to this screen.

B. Questionnaire (extract)

Original questionnaire

We will now ask you questions about your personal situation. Remember, all data is anonymous and will not be shared with a third party.

Gender

What is your gender?

- Male
- Female
- Other

Age

What is your date of birth?

Household size

How many people live in your household (including yourself?)

- 1 adults and children
- ...
- 10 adults and children
- more than 10 adults and children

Education

What is the highest level of education you have completed?

- Less than high school
- High school
- Some college
- Diploma, trades certificate or other post school qualification other than university
- Undergraduate degree (e.g. BA, BS)
- Post-graduate degree

Mother's education

What is the highest level of education that your mother completed?

- Less than high school
- High school
- Some college
- Diploma, trades certificate or other post school qualification other than university
- Undergraduate degree (e.g. BA, BS)
- Post-graduate degree

Father's education

What is the highest level of education that your father completed?

- Less than high school
- High school
- Some college

- Diploma, trades certificate or other post school qualification other than university
- Undergraduate degree (e.g. BA, BS)
- Post-graduate degree

Income

In the last 12 months, what was your total income, the income that you received as an individual, before taxes have been deducted? (Income can come from salaries and wages, profit from self-employment, interest, rent, pension, social insurance payments and other benefits, among others)

Employment status

Which of these best describes your situation?

- Employee
- Employer / self-employed
- Unemployed
- Outside the labour force (e.g. homemaker, student, retired, unable to work)

Marital status

What is your marital status at present?

- Single
- Married cohabitating with spouse
- Married non-cohabitating with spouse (actually separated)
- Legally separated
- Divorced
- Widowed

Size of area of residence

Do you live in a?

- Rural area
- Village (less than 50,000 inhabitants)
- Town (50,000 to 200,000 inhabitants)
- Small metropolitan area (200,000 to 500,000 inhabitants)

- Medium-sized metropolitan area (500,000 to 1.5 million inhabitants)
- Large metropolitan area (more than 1.5 million inhabitants)

Personality traits — 15-item Big Five Inventory (adapted from Ubbiali, Chiorri, Hampton & Donati, 2013)

To what extent do you agree with the following statements?

1. I see myself as someone who is sometimes somewhat rude to others.
2. I see myself as someone who has a forgiving nature.
3. I see myself as someone who is considerate and kind to others.
4. I see myself as someone who tends to be lazy.
5. I see myself as someone who does a thorough job.
6. I see myself as someone who does things effectively and efficiently.
7. I see myself as someone who is relaxed, handles stress well.
8. I see myself as someone who gets nervous easily.
9. I see myself as someone who worries a lot.
10. I see myself as someone who is reserved.
11. I see myself as someone who is outgoing, sociable.
12. I see myself as someone who is communicative, talkative.
13. I see myself as someone who values artistic experiences.
14. I see myself as someone who has an active imagination.
15. I see myself as someone who is original, comes up with new ideas.

- Disagree strongly
- Disagree a little
- Neither agree nor disagree
- Agree a little
- Agree strongly

Municipality of residence

In what municipality do you currently live?

Generalized trust question

On a scale from zero to ten, where zero is not at all and ten is completely, in general how

much do you trust most people?

- Not at all
- ...
- Completely

Frequency of voluntary works

How often do you participate in voluntary activities to help people other than your direct relatives, friends or colleagues?

- Daily
- Several days a week
- Once a week
- Less than once a week
- Never

Frequency of encounters with friends

How often do you get together with friends?

- Daily
- Several days a week
- Once a week
- Less than once a week
- Never

Connectedness with neighbours

How strongly do you feel connected to other people in your neighborhood?

- Not at all
- ...
- Very connected

Voted in elections

Did you vote in the last general elections?

- Yes
- No

- I couldn't vote

Follow-up questionnaire

Municipality of birth

In what municipality were you born?

Municipality of childhood

Where did you spend most of your childhood until age 16?

- Municipality of birth
- Current municipality of residence
- Other municipality. Which?

Mother's place of birth

In which province was your mother born?

Father's place of birth

In which province was your father born?

Importance of family

Indicate how important is family in your life. Would you say it is:

- Not at all important
- Not very important
- Rather important
- Very important

Children's responsibilities towards parents

With which of these two statements do you tend to agree?

A. One does not have the duty to respect and love parents who have not earned it by their behavior and attitudes.

B. Regardless of what the qualities and faults of one's parents are, one must always

love and respect them.

Parents' responsibilities towards children Which of the following statements best describes your views about parents' responsibilities to their children?

A. Parents have a life of their own and should not be asked to sacrifice their own well-being for the sake of their children.

B. A Parent's duty is to do their best for his or her children even at the expense of their own well-being.

Social risk vignette

For personal reasons, you have to travel to a big city. From the airport you can choose between two taxi companies to reach your final destination for which you don't know the exact route. Company A charges you a fixed price of $12. Company B charges you according to the taxi-meter. If the driver takes the direct route, it costs you $8. However, 1 out of 5 drivers take detours to make more money and the fare is then $16. Which company would you choose?

- Company A
- Company B

Natural risk vignette

For personal reasons, you have to travel to a big city. From the airport you can choose between two taxi companies to reach your final destination for which you don't know the exact route. Company C charges you a fixed price of $12. Company D charges you according to the taxi meter. If the weather is fine, it costs you $8. However, 1 out of 5 times, due to bad weather conditions the ride takes longer and the fare is then $16. Which company would you choose?

- Company C
- Company D

References

Ubbiali, A., Chiorri, C., Hampton, P., & Donati, D. (2013). Italian big five inventory. psychometric properties of the italian adaptation of the big five inventory (BFI). *Bollettino di Psicologia Applicata*, *59*(266), 37–48.
